# Twenty Years of Attachment Research With the Friends and Family Interview: A Systematic Review and Meta‐Analysis

**DOI:** 10.1002/cpp.70203

**Published:** 2026-01-16

**Authors:** Stefania Muzi, Howard Steele, Cecilia Serena Pace

**Affiliations:** ^1^ Department of Educational Sciences (DISFOR) University of Genoa Italy; ^2^ Psychology Department the New School for Social Research New York New York City USA

**Keywords:** attachment, childhood, FFI, Friends and Family Interview, meta‐analysis, systematic review

## Abstract

**Objective:**

This systematic review and meta‐analysis assessed the Friends and Family Interview (FFI) as a tool for measuring attachment in middle childhood and adolescence.

**Methods:**

A comprehensive search was conducted following PRISMA guidelines across Scopus, Web of Sciences, PsycINFO and PubMed, covering literature from 2004 to 2024. Out of 3000 screened records, 52 studies were selected for narrative synthesis and 32 for meta‐analysis. Data extraction used a structured coding protocol and quality was assessed with the Newcastle‐Ottawa Scale.

**Results:**

The meta‐analysis included 2867 participants, revealing significant study heterogeneity. At‐risk samples (*k* = 13, *n* = 598) had a higher prevalence of insecure attachment compared to community samples (*k* = 11, *n* = 1080). Clinical samples (three, all with anxiety disorders) mostly showed secure classifications. Cultural differences in attachment were observed between European and Northern American or Latin samples. Attachment disorganization scores correlated positively and significantly with internalizing and thought symptoms, while scores for Security, Insecure‐Dismissing, Coherence, Secure Base/Safe Haven mother and father scores were associated to verbal IQ, with moderating effects of (older) age, (female) gender and sample risk.

**Conclusions:**

The FFI shows potential as an attachment assessment tool, but issues like study heterogeneity and cultural bias require further research to improve its applicability across diverse populations.

## Summary

- The Friends and Family Interview (FFI) is a promising tool for assessing attachment in middle childhood and adolescence, demonstrating good psychometric properties.
- Research indicates that at‐risk samples show a higher prevalence of insecure attachment compared to community samples, highlighting the importance of targeted interventions.
- Cultural differences in attachment patterns have been observed, with a need for more diverse research to enhance the applicability of the FFI across various populations.
- FFI scores showed associations with internalizing and thought problems and verbal IQ only partially aligning with results obtained with other methods.
- Training in administering and coding the FFI is essential, as it is a paid resource; practitioners should weigh the benefits of this investment against their specific needs.

## 1 Introduction

Attachment scholars and practitioners worldwide agree on the potential utility of assessing attachment throughout the lifespan using empirically validated methods (Rodríguez 2020; van Hoof et al. 2015), although the most extensively validated and widely used methods concern infancy (Strange Situation Procedure [SSP], Ainsworth et al. 1978) or adulthood (Adult Attachment Interview [AAI] George et al. 1985; and the companion multidimensional scoring system, Main et al. 2003; Main et al. 2008). The Strange Situation has been the recent focus of a meta‐analysis on the first ‘20,000 Strange Situations’ (Madigan et al. 2023), while the AAI has been the focus of a recent meta‐analysis on ‘the first 26,000 AAIs’ (Bakermans‐Kranenburg et al. 2025). This highlights the need for research informing us about the psychometric characteristics, psychopathological correlates, strengths and limitations of other attachment research methods.

Such research is particularly crucial when assessments occur in late childhood and adolescence—dynamic periods of significant developmental changes that affect the attachment system (and have only become a central focus of study in the last 20 years) (Allen and Tan 2016; Bosmans and Kerns 2015). When available to provide care, parents remain the preferred attachment figures in middle childhood and adolescence, fostering healthy development and offering protection (through supervision and support) against risk associated with exclusive reliance on peer relationships at this age (Kobak et al. 2007; Moretti and Peled 2004). At the same time, from middle childhood, peers, such as friends, siblings and eventual romantic partners become increasingly significant in the attachment hierarchy (Allen and Tan 2016). Indeed, exploration behaviours increase and behaviours aimed at seeking closeness to parents gradually decrease, being redirected toward peers especially when attachment feelings are activated by ordinary difficulties in the socio‐relational context (e.g., poor grades at school, conflicts with parents or other friends). In contrast, when attachment feelings are activated by more serious and harmful situations (e.g., an accident involving physical damage to the self or others), proximity seeking behaviours remain directed towards parents (Allen and Tan 2016; Moretti and Peled 2004). Simultaneously, the maturation of metacognitive skills enables children to handle abstract representations of people and relationships (Steele and Steele 2005). This development allows them to restructure their understanding of relationships with attachment figures—beginning with their parents—toward greater complexity and nuanced completeness. However, attachment insecurity can significantly impact the adaptive functioning and healthy development of children and adolescents (Allen and Tan 2016), highlighting the importance of conducting an accurate evaluation of attachment during these developmental stages.

The recent AAI meta‐analysis notes that dismissing tendencies may be typical in adolescence and its exclusive focus on parental relationships may lead to overestimation of dismissing (Ds) classifications in this age group (Bakermans‐Kranenburg et al. 2025). For this reason, research over the last 20 years has involved attachment researchers' new efforts to explore psychometric and clinical properties of potential alternative interviews able to capture the contribution or multiple specific relationships (best friend, favourite teacher, sibling, as well as relationship to mother and to father) to general attachment, uniquely addressed by the Friends and Family Interview (Steele and Steele 2005).

### 1.1 The Friends and Family Interview: Commonalities and Differences With Other Interviews

The Friends and Family Interview (Steele and Steele 2005; Steele et al. 2009) is a semi‐structured interview tailored for middle childhood and adolescence, which includes 27 questions administered in‐person or over Zoom, in one single session lasting around 45–60 min. Originally used intended for the age range 10–15, it has been later applied from ages 7 to 19 (Psouni and Apetroaia 2014; Muzi and Pace 2022; Terrone et al. 2021). However, psychometric research supporting the various applications of the FFI, especially those related to psychometrics, is limited compared to the more validated AAI (Bakermans‐Kranenburg et al. 2025). Unlike the AAI and the Child Attachment Interview or CAI (Shmueli‐Goetz et al. 2008), the FFI questions inquire about a wide array of adolescent life areas beyond relationships with parents, specifically self‐representation and relationships with peers ‐both within intimate relationships with a best friend and less intimate in the school context‐, with a favourite teacher and siblings. The FFI questions thoroughly explore the adolescents' viewpoint on intricate and sometimes conflicting emotions within their closest relationships, explicitly asking about strategies employed to deal with distressful or new and challenging situations. Participants are encouraged to substantiate their evaluations with personal episodic details and to reflect on others' points of view concerning them.

The interview is video or audio‐recorded to be transcribed and/or rewatched to assign ratings according to a manualized coding system (Steele et al. 2009), informed by yet distinct from the AAI coding system (Main et al. 2008). Specifically, within the psycholinguistic approach, scores from 1 (no evidence) to 4 (marked evidence) are assigned to several scales covering eight domains of functioning, i.e., *1. Coherence, 2. Reflective Functioning*, *3. Parental availability as Secure‐base/Safe‐haven [SB/SH]*, *4. Self‐regard (which includes also school and social competence)*, *5. Best Friend's relationship*, *6. Sibling(s) relationship(s)*, *7. Affective regulation strategies*, *8. Differentiation of Parental Representations*. Following the guidelines, a rater can evaluate combinations of scores in these domains to assign a final 1–4 score on four global rating scales, one indicating the prevalence of attachment security, i.e., *Secure‐autonomous* [S] and three insecure patterns, i.e., *Insecure‐Dismissing* [Ds], *Insecure‐Preoccupied* [P] or *Disorganized‐Disoriented* [D] (see below for a detailed description of these patterns or classifications). The highest of these dimensional scores for the patterns corresponds to the best‐fitting attachment category on a two‐way system (Secure‐Insecure) and three‐way (S, Ds and P) or four‐way system (S, Ds, P and D). Additionally, an evaluator could optionally perform video coding on nonverbal signals to code two additional nonverbal scales, *Fear/Distress and Frustration/Anger*, that have not yet been reported in literature as all published reports including the FFI have been based on study and rating of the full verbal transcript—an approach scoring modelled after the close attention paid to spoken language, prompted and spontaneous, as is vital to the AAI scoring system (Main et al. 2008).

Like the AAI and CAI, this FFI psycholinguistic‐informed coding primarily relies on scores in the domain of *Narrative coherence*, i.e., adherence to Grice's maxims of conversation (Grice 1975). This includes truthful adherence between descriptive content and episodic memories supporting statements and the ability to succinctly remain focused on the topic for at least most of the interview (including the narration of painful and unfavourable experiences in the past) and present or current conversational collaboration with the interviewer. Additionally, conventional approaches to scoring FFIs (Muzi et al. 2022; Kerns et al. 2022; Psouni and Apetroaia 2014; Psouni et al. 2020) support the importance of considering high scores in Secure‐Base/Safe‐Haven scales toward mother and father to assign higher scores for coherent and higher scores on the scale measuring evidence for the Secure‐autonomous overall pattern.

Relying primarily, but not only, on these above mentioned scores, usually the raters attribute a *Secure* or *Secure‐Autonomous* classification (i.e., the highest score in the secure‐autonomous pattern scale) when the interviewee demonstrates adaptability, comfort in discussing attachment experiences and depending on or missing others and the capacity to seek help when upset or facing new experiences, especially relying on parents' availability. Individuals whose interviews are rated high for *Security* show in language their valuing of attachment relationships with infrequent or absent violations of Grice's maxims. Differently, interviews deemed *Insecure* display in their language marked reliance on defensive affective regulation strategies, low narrative coherence and minimal evidence of reflective functioning skills and the respondent recalls none of only partially supportive memories of relying on parents when distressed, resulting in low scores for Safe Haven or Secure Base re mother or father. Specifically, an *Insecure‐Dismissing* (Ds) classification is assigned when narratives exhibit defensive affective regulation strategies such as *Idealization* (i.e., a discrepancy between positive descriptions of the parents and lack of memories in support of them, resulting in violations of Grice's maxims of Quality or Truthfulness and Economy, i.e., recalling and saying too little) and *Derogation* of parents (i.e., devaluing of attachment experiences), with minimal recognition of distressing emotions. An *Insecure‐Preoccupied* (P) classification is applied when there are elevated scores given for defensive emotion regulation strategies within interpersonal relationships such as involving *Anger* or *Role‐reversal* towards parents, which interfere with the interviewee's to maintain a focus on questions asked, with a resulting deficit in the sense of collaboration with the interviewer, often resulting in speech that is excessively long, verbose without a clear or balanced focus. Finally, an *Insecure‐Disorganized* (D) classification is used when there are disruptions in speech that suggests a monitoring of what is reasonable, especially when the respondent is discussing potentially traumatic experiences, accompanied by fear, lapses in coherence, bizarre content and conflicting strategies (down‐regulating dismissal at times, e.g., re father, and up‐egulating preoccupation at other times, e.g., re mother) in attachment narratives (Steele et al. 2009). Notably, unlike the AAI where the dimensional and categorical coding procedure of (unresolved) disorganization versus losses and abuses is complex and measured through multiple indicators (e.g., lapses in reasoning and monitoring re loss/trauma), the FFI psycholinguistic‐based coding system of the disorganized pattern has been noted as potentially less sophisticated especially because the FFI does not explicitly inquire about losses and potentially traumatic experiences through specific questions (Pace et al. 2020). Instead, disorganized responses to the FFI are indexed by signs of fear or extreme distress in response to the question ‘what do you do when you are emotionally hurt or upset’ with a focus on the video‐filmed response that allows attention to non‐verbal cues from the interviewee.

### 1.2 Relevance of a Systematic Review on the Friends and Family Interview

Given the above, the FFI can be a promising age‐adapted interview to assess attachment during middle childhood and adolescence. However, Pace (2014) observed that the FFI has been used almost exclusively on at‐risk populations of adopted children, whilst fewer studies provided FFI data from further types of adolescent populations such as those being low‐risk with typical functioning, at‐risk due to other conditions (i.e., out‐of‐home childcare, physical disease and dysfunctional parents) and clinical samples with psychiatric diagnoses. Although reviews of the AAI (Bakermans‐Kranenburg et al. 2025) and the CAI (Privizzini 2017; Gastelle and Kerns 2022) have synthesized data in this regard, a similar synthesis of FFI literature is lacking. Considering that more than ten years have passed since the Pace (2014) observation, a systematic review of the FFI literature should provide an updated, comprehensive picture of the current uses of the FFI and enable comparison of FFI results with those obtained using other attachment interviews.

Furthermore, the FFI literature reports conflicting results regarding the possible existence of age, gender (e.g., Decarli et al. 2022; Schmidt et al. 2022) or cultural (Barcons et al. 2012; Esbjørn et al. 2015) differences in attachment, fueling open debates in the attachment literature (e.g., Bakermans‐Kranenburg et al. 2025; Gloger‐Tippelt and Kappler 2016; van Ijzendoorn and Sagi‐Schwartz 2008). Moreover, studies employing the FFI typically use single convenience samples composed of only children or adolescents, rather than large samples that are age‐representative. As sample size and representativeness can affect the results of social research (Borenstein et al. 2021; Guyatt et al. 2008), it is important to evaluate the quality of the evidence provided by FFI studies, as poor study quality (Guyatt et al. 2008) can affect and potentially distort the results. Thus, reporting and exploring the quality of the literature with the FFI can help readers to balance their interpretation of research results.

Additionally, the cited review by Gastelle and Kerns et al. (2022) reviewed psychometric evidence concerning the FFI but with selection criteria including only studies involving samples in middle childhood and early adolescence, also highlighting little research after 2018, potentially discouraging scholars and practitioners from considering this interview for their research or clinical use in older participants. In this regard, there have been recent efforts to fill this gap in the psychometric evidence concerning the FFI (e.g., Pace et al. 2020, 2023; Psouni et al. 2020), with encouraging results suggesting its relevance to mid‐adolescence and older youth. At the same time, attention should be focused on the quality and coverage of existing FFI knowledge and what gaps still require investigation. In a similar vein, in line with a primary goal of attachment research that has consistently explored the impact of attachment on individual functioning and development, this paper aims to provide a comprehensive summary of what psychological variables have been investigated in FFI studies that can illuminate established findings and contribute to new directions for future FFI research.

Twenty years after the FFI was first introduced to the field (Steele and Steele 2005), a systematic review and meta‐analysis focused on the FFI could help substantiate existing knowledge on psychometric properties, current trends and uses and the clinical utility of the interview. Merging samples in different conditions could enhance solidity or contrast results obtained by single studies, providing information about the ability of the FFI to yield results convergent with existing literature and with other reliable attachment interviews (e.g., AAI and CAI) in terms of attachment category distribution, differences in attachment according to the nature of the sample (e.g., van den Dries et al. 2009), demographics or country (Bakermans‐Kranenburg et al. 2025; van Ijzendoorn and Sagi‐Schwartz 2008) and relationships with other variables found in the broader attachment literature, e.g., emotional‐behavioural problems, cognitive skills (Eilert and Buchheim 2023; Madigan et al. 2016), accounting for potential confounds in each prior study.

### 1.3 Research Questions

With these premises, the current systematic review and meta‐analysis aim to answer the following research questions [RQ]:

1. What is the aggregate distribution of attachment categories and/or pooled means on the scales in the FFI in community, at‐risk and clinical samples?
2. Are participants' age and gender and quality score moderators of RQ1 results?
3. Are there differences in category distribution and/or means based on participants' type of sample (community or at‐risk) and cultural background?
4. a. What are the main trends and gaps in FFI research concerning psychometric properties and relationships with other constructs (e.g., emotion‐behaviour problems, verbal IQ)? Are relationships between attachment and other constructs identifiable on a meta‐analytic level?

## 2 Method

This systematic review was conducted following the latest PRISMA guidelines (Page et al. 2021) and is registered on PROSPERO, protocol n. CRD4202342908.

### 2.1 Search Strategy

After ensuring the absence of overlapping systematic reviews by browsing PROSPERO and registering the protocol, a comprehensive search strategy (reported in Appendix S1) was used across databases Scopus, Web of Sciences (all databases), PsycINFO, PsycArticles, Behavioural Science Collection, Pubmed (including Medline) and Google Scholar. The search was applied from 2004, as 2005 was the year of the official release of the interview but preliminary contributions could have been published earlier. No other filters beyond the year of publication were applied during the search and MeSH terms were also considered.

The first search occurred on 04/05/2023, followed by three search updates to include contributions published after that date, on 08/03/2024, 20/05/2024 and 08/10/2024, with additional exclusion criteria in the screening process of being a ‘duplicate contribution’.

After the full‐text screening phase, the second author—who is an FFI author with extensive knowledge of unpublished literature—checked the existence of grey literature (unpublished dissertations and conference proceedings). This was followed by a second search for grey literature, which the first author conducted on the first 200 records of specific databases to search for unpublished contributions, e.g., OATD, SRNN and PsyArxiv. No additional contributions that had not been previously found and screened emerged.

### 2.2 Eligibility Criteria

Further screening process occurred to select contributions based on inclusion criteria of:

1 *Pertinence of the construct*, i.e., employed the Friends and Family Interview.
2 *Presence of original data*: only empirical studies, both with a quantitative or qualitative approach including also single case studies, that reported original data of at least one case (i.e., at least one attachment category or one FFI patterns or overall coherence or SB/SH scales mean or results from a qualitative analysis or results concerning psychometric characteristics or relationships with other constructs). Contributions included scientific journal articles, dissertations, books or conference proceedings.
3 *Language*: Only contributions in English. No restrictions related to participants' demographics were applied to the meta‐analytic research questions. Particularly, age restrictions were not applied because, although the FFI was originally developed for an age range between 10 and 17 years, the authors were aware that some studies used it with younger or older participants.

And exclusion criteria:

4 *Theoretical or non‐original contributions*: Contributions were excluded if the title, abstract or keywords indicated a theoretical nature, such as theoretical articles, commentaries and letters to the editor. Additionally, non‐original contributions, such as narrative reviews, were also excluded.

Additional exclusion criteria only for the meta‐analysis, after finishing the study selection:

5 *Non‐original data*: Studies involving the same participants as other included studies, i.e., duplicate samples, were excluded if they reported a smaller sample size or provided less empirical information. When the study was longitudinal, only the first data collection was considered.
6 *Absence of original data concerning at least one FFI category or pattern*, *overall coherence or SB/SH scale mean*, in at least one case.

### 2.3 Study Selection

**FIGURE 1:**
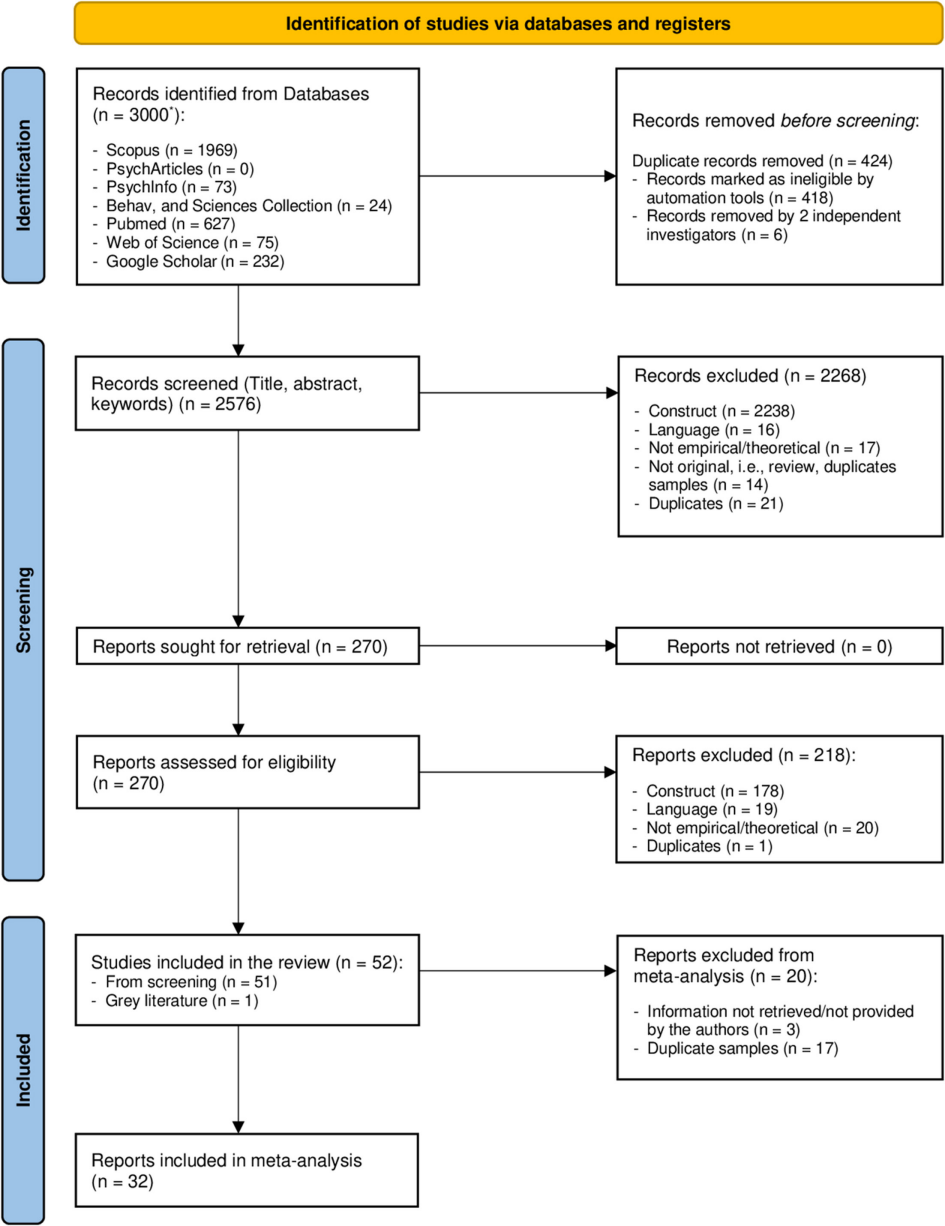
PRISMA 2020 flow diagram for new systematic reviews which included searches of databases and registers only.

Details of the entire study selection process are graphically reported in the flowchart in Figure 1 shown below.

Figure 1 reveals that, overall, the search strategy returned to 3000 records. First, two independent investigators (trained graduate students in clinical psychology supervised by the first author) removed duplicates through free Zotero software, reaching a 99.36% agreement on removals (Cohen's *k* = 0.97). After that, they proceed to a first screening by applying the inclusion and exclusion criteria stated above to the title, abstract and/or keywords of contributions, reaching 98.17% of agreement (*k* = 0.91) and identifying 21 additional duplicates. After this first screening, all authors proceeded to retrieve full texts of the 270 contributions selected, being able to retrieve all of them. The same students independently screened full texts according to selection criteria, identifying another duplicate which was removed and reaching 97.86% agreement on eligible contributions, *k* = 0.92, SE = 0.033, 95% Confidence Intervals [95% CI] 0.855–0.983. Fifty‐two contributions were selected as eligible after this second screening phase plus inquiring the expert for grey literature retrieval. Disagreements in both screening phases were resolved by consensus upon the judgement of the first author as a third independent investigator.

When eligible full texts did not contain the necessary information to answer research questions, the first author and another graduate student contacted the corresponding authors twice asking for missing and/or unpublished data, from July to December 2023 for the contributions included in the first search and from March till October 2024 concerning contributions included with the search updates. When the corresponding author was unreachable (e.g., due to an inactive email address or retirement), the other authors were contacted. Most authors provided data after the second e‐mail, so retrieving missing data lasted more than 10 months. Finally, 52 contributions were included for the narrative synthesis of which 32 were also in the meta‐analysis (see Figure 1).

### 2.4 Data Extraction

An ad hoc coding protocol was developed to extract and encode the necessary information for review purposes. The extracted information and eventual re‐coding for meta‐analyses were:

a ***Contribution metadata and characteristics***, such as the author(s), year of publication, country, design (i.e., cross‐sectional, case–control or longitudinal) and score received in the quality assessment (see below).
b ***Participants' characteristics***, including:

- sample *size*,
- *type* of sample, coded in groups as community = 1 (i.e., typical functioning, without diagnoses of mental or physical illness, nor factors of vulnerability as described in the at‐risk samples), at‐risk = 2 (i.e., at increased risk of developing a mental disorder due to low socioeconomic status, adverse childhood experiences, having parents who are incarcerated or have diagnoses or due to biographical risk factors such as being adopted, in foster care, institutionalized or having neurodevelopmental disorders and chronic physical illnesses) or clinical = 3 (i.e., with psychiatric diagnoses).
  - *gender* (coded as the percentage of males).
  - *age* (coded in year: range, mean and standard deviation).
  - *cultural background*, coded based on the nationality of the participants in regions as defined by United Nations (2024), i.e., Western European = 1, Eastern European = 2, North American = 3, Latin American and Caribbean States = 4, Asia‐Pacific = 5, Africa = 6.

c ***FFI outcomes***, specifically:
  - *percentage* of participants classified as Secure (= 1) or Insecure (= 0) with the two‐way system or F/S (= 1), Ds (= 2), P (= 3) and D (= 4) with the four‐way system in the FFI.
  - *means and standard deviations* in any available FFI scale.
  - *Pearson's correlation coefficient* or other values indicating links between FFI scales and other variables assessed in at least 2 samples from different studies for RQ4.
d ***Results for narrative synthesis***, including qualitative, psychometrics or relationships with other constructs.

### 2.5 Quality Assessment

The first author and one graduate student performed the quality assessment applying the *Newcastle‐Ottawa Quality Assessment Scale* [NOS], modified for non‐randomized studies (Wells et al. 2016). This scale evaluates three domains: *Selection*, *Comparability* and *Outcome*. In these areas, evaluators assigned up to 5, 2 and 3 stars respectively, based on specific criteria. These criteria include the sample's representativeness, selection methods, ascertainment of exposure and demonstration of originality of the outcome of interest (for *Selection*); control of demographics and additional other confounding variables (for *Comparability*); Outcome assessment and follow‐up appropriateness if applicable (for *Outcome*). The total score, ranging from 0 to 10, was calculated by summing the scores in each domain. A score of 7 or above indicated high quality, 4 to 6 indicated moderate quality and 3 or below suggested a high risk of bias.

### 2.6 Analytic Plan

For systematic review purposes, a narrative description of all contributions was provided with the support of tables and narrative synthesis was provided in response to RQ5.

For the remaining research questions, a meta‐analysis approach was used when feasible, i.e., with at least one observation from at least two samples from two different studies (Valentine et al. 2010). In the case of community adolescents screened at‐risk or not through questionnaires (e.g., Pace et al. 2021; Schmidt et al. 2022) data were merged and considered a unique community sample as a formal diagnosis was not established. Only data about attachment patterns, overall coherence and Secure Base/Safe Haven scales were meta‐analysed due to the relevance of these scales to assign the final attachment category that aligns with the broader attachment literature involving the AAI, CAI and FFI reported in the introduction.

Analyses were performed through R software packages ‘metafor’ (Viechtbauer 2010) and ‘esc’ (Lüdecke 2019), according to indications of Harrer et al. (2021), reporting 95% Confidence Intervals [95% CI].

To answer RQ1 (aggregate distribution of attachment categories and/or pooled means in community, at‐risk and clinical samples), pooled percentage distributions and weighted mean scores in FFI scales for patterns, overall coherence and SB/SH parents were calculated separately in community, at‐risk or clinical samples. The weighted pooled data of the community group was used as control group data to compute Effect Sizes [ES] for RQ2 (moderation of age, gender and study quality) and RQ3 (attachment differences according to sample type and cultural background). For the meta‐regression, the weighted means in each group were used separately as the outcome, to reduce the sample type as a source of heterogeneity if previously detected. To preliminary explore heterogeneity, a random‐effect model to account for expected variability across studies (Cooper and Hedges 1994; Rosenthal 1995) was employed, estimating tau (*τ* ^2^) for between‐study variance through the Restricted Maximum Likelihood (REML) estimator method on weighted means and Higgins and Thompson's *I* ^2^ to account heterogeneity due to sampling errors. *Q*test was also performed to explore the necessity of robust method employment. Eggers' *z* test and funnel plots were inspected to explore the existence of publication biases. In case of significance of these analyses, ES were adjusted with a trim‐and‐fill method.

To answer RQ2, meta‐regression techniques were used separately in each sub‐group of community, at‐risk or clinical participants, reporting Q test results.

To answer RQ3, Odds Ratio [OR], Risk Ratio [RR] and Risk Difference [RD] were calculated, comparing community vs. at‐risk, community vs. clinical and at‐risk vs. clinical group on the possibility of being classified as secure vs. insecure, or S vs. Ds, or vs. P, or vs. D and between diverse insecure classifications. Concerning OR, comparisons were considered statistically significant when the 95% CI contained|1|. RR was considered in values lower or greater than 1 statistically significant with *p* < 0.05. Further, Hedges'*g* was computed as Standardized Mean Difference (SMD) on continuous FFI scores. Hedges'*g* was preferred because small samples and independence of groups were expected (Borenstein et al. 2021). Similarly to Cohen's *d*, Hedges' *g* effect in terms of group differences can be interpreted as small (0.2), medium (0.5) or high (0.8). The same approach was used on pooled percentage distribution and weighted average in available FFI scales were calculated for the Northern American cultural background considered as control given the FFI release study is from the United States of America (Steele and Steele 2005) and ORs, SMD and Hedge's g were calculated by comparing each other cultural background with the former.

To answer RQ4 (relationships with other constructs), first, effect sizes [ES] as standardized Pearson's correlation coefficients were calculated for all available samples and separately for each psychopathological syndrome (e.g., internalizing, externalizing) and verbal‐IQ. ES were calculated by first converting Pearson's r to Fisher's z and then backtransformed to standardized r values. For each effect size, 95% CI were computed (Rosenthal 1995) and the pooled ES was considered statistically significant if the CI did not contain 0, with strengths: |0.15| as small, > |0.25|medium, ≥ |0.35|as large (Gignac and Szodorai 2016). Moderators were explored meta‐regression techniques with robust method, considering sample risk status (0 = community, 1 = at‐risk, 2 = clinical), gender (% males), age and study quality as potential moderators of relationships detected.

## 3 Results

### 3.1 Study Characteristics

Table 1 details all studies included in the systematic review and meta‐analysis.

**TABLE 1:** Main characteristics of the included studies in the systematic review^a^ (*N* = 52) and meta‐analysis (*N* = 32).

Study	Country	Design	N	Sample features					Quality
				Type	Gender	Age		Cultural background^b^	
					% Males	Range	M (SD)		
Barcons et al. (2012) Abrines et al. (2012) Barcons et al. (2014)^a^	Spain	Cross‐sectional	116	At‐risk (adopted)	46.60	8–11	8.92 (1.08)	WE	7
Bastianoni et al. (2020)	Italy	Cross‐sectional	13	At‐risk (adopted)	54	12–17	14 (1.98)	WE	7
Breinholst et al. (2018)	Denmark	Case–control	222	Sample 1, 111 community Sample 2, 111 clinical (anxiety)	47.70 47.70	7–13 7–13	9.75 (1.47) 9.75 (1.47)	WE	6
Carone et al. (2023)	Italy	Cross‐sectional	76	Community Sample 1, *n* = 55 with gay/lesbian parents	54.54	6–11	8.74 (2.12)	WE	8
				Sample 2, *n* = 21 with heterosexual parents	42.86	6–11	8.49 (2.29)		
Decarli et al. (2022)	Luxembourg	Cross‐sectional	42	Community	51	11–17	14.2 (1.83)	WE	7
Esbjørn et al. (2015)	United States of America (USA)	Longitudinal	34	At‐risk (low‐income ethnic minority)	55.90	10–12	10.45(0.60)	NA	7
Escobar et al. (2013)	Spain	Cross‐sectional	40	At‐risk (adopted)	55	11–16	12.63 (1.28)	WE	7
Escobar et al. (2014)	Chile	Case–control	50	Sample 1, *n* = 25 At‐risk (adopted)	54.9	11–18	12.78 (1.65)	LA	6
				Sample 2, *n* = 25 Community	56	11–18	12.96 (1.79)		
Frazier and Knapp (2020)^a^	USA	Longitudinal, case–control	50	At‐risk (foster care), Sample 1, *n* = 25 benefiting from equine‐assisted psychotherapy (EPA) Sample 2, *n* = 25 no‐intervention.	—	13–18	—	WE	NR
Groza (2016)	Romania	Cross‐sectional	63	At‐risk (adopted)	35	11–16	12.97 (2.07)	EA	7
Hillman et al. (2024) Hillman et al. (2023)^a^	United Kingdom (UK)	Longitudinal	70	At‐risk (adopted, merged sample of 35 early adopted, 35 late adopted)	48.55	12–15.75	13.59 (0.90)	WE	7
Kerns et al. (2015)	USA	Cross‐sectional	107	Community	35.5	10–15	11.99 (1.04)	NA	6
Kerns et al. (2022) Gastelle (2018)^a^ Obeldobel and Kerns (2020)^a^ Koehn and Kerns (2018)^a^	USA	Cross‐sectional	92	Community	63	10–14	11.91 (1.25)	NA	6
McConnachie et al. (2020)	UK	Case–control	97	Community Sample 1, *n* = 30 with gay parents	76.7	10–14	11.48 (1.06)	NA	7
				Sample 2, *n* = 29 with lesbian parents	37.9	10–14	11.83 (1.42)		
				Sample 3, *n* = 38 with heterosexual parents	52.6	10–14	11.13 (1.03)		
Muntean and Ungureanu (2012)	Romania	Case–control	40	At‐risk (adopted)	37.5	11–16	13.10 (1.70)	EA	0
Muzi and Pace (2023) Muzi and Pace (2020, 2022)^a^ Muzi et al. (2022)^a^	Italy	Case–control, cross‐sectional	46	At‐risk (residential care)	59.6	11–19	15.66 (2.09)	WE	6
Muzi et al. (2024)	Italy	Cross‐sectional	36	At‐risk (residential care)	41.7	14–19	16.5 (2.13)	WE	8
Ongari et al. (2013)	Italy	Cross‐sectional	4	At‐risk (adopted)	100	12–14	13.60 (1.05)	WE	1
Pace et al. (2020) Muzi and Pace et al. (2022, 2023)^a^ Muzi et al. (2022)^a^	Italy	Cross‐sectional	110	Community	50	11–17	14.22 (1.84)	WE	7
Pace et al. (2021) Pace et al. (2020)	Italy	Cross‐sectional, case–control	112	Community Sample 1, *n* = 56 no risk Sample 2, *n* = 56 risk of binge eating	0	14–18	16.40 (1.30)	WE	7
Pace et al. (2022) Pace et al. (2015, 2016, 2018, 2019, 2023)^a^ Muzi and Pace (2022, 2023)^a^	Italy	Longitudinal, cross‐sectional	79	At‐risk (adopted)	51.9	11–18	14.03 (2.00)	WE	7
Pace et al. (2023) Muzi et al. (2021, 2022)^a^	Italy	Longitudinal	102	Community	45.1	12–17	14.93 (1.63)	WE	7
Pandya (2023)	India	Longitudinal, case–control	86	At‐risk (adopted) Sample 1, *n* = 43 no intervention	41.86	11–15	13.23 (1.67)	AS	7
				Sample 2, *n* = 43 intervention	44.19	11–15	13.36 (1.34)		
Peñarrubia et al. (2023)	Spain	Case–control	67	Sample 1, *n* = 29 at risk (adopted)	75.9	8–13	10.36 (1.27)	WE	7
				Sample 2, *n* = 38 Community	55.3	8–13	11.04 (1.36)		
Pierrehumbert et al. (2011)	Romania	Cross‐sectional	32	At‐risk (adopted)	38	11–16	12.84 (1.61)	EA	5
Psouni and Apetroaia (2014)	Sweden	Cross‐sectional	120	Community	44	8–12	10.09 (0.94)	WE	5
Psouni et al. (2020)	Sweden, Denmark	Cross‐sectional	341	Community	46	8–12	10.30 (1.36)	WE	6
Schmidt et al. (2022)	UK	Cross‐sectional	90	At‐risk (first admission to a community clinic for anxiety or depressive symptoms)	47	9–17	13.04 (2.72)	WE	7
Slutsky et al. (2016)	UK	Cross‐sectional	19	At‐risk (single mothers)	21	12–19	14.18 (2.20)	WE	7
Steele and Steele (2005)	USA	Longitudinal	55	Community	49	11–12	11.60 (3.80)	NA	5
Stievenart et al. (2012)	Belgium, Romania	Cross‐sectional	78	Community Sample 1, *n* = 35 Belgian	56.3			WE	NR
				Sample 2, *n* = 43 Romanian					
Tamari et al. (2024)	UK	Longitudinal	108	Community	50	12–13	12.90 (0.24)	WE	7
Terrone et al. (2021)	Italy	Cross‐sectional	178	Community	41	16–22	17.51 (0.82)	WE	6
Walczak et al. (2017)	Denmark	Case–control	69	Clinical (anxiety) Sample 1, *n* = 32 responders to treatment	43.8	7–13	9.78 (1.66)	WE	6
				Sample 2, *n* = 37 non responders	37.8	7–13	9.89 (1.71)		
Zadeh et al. (2017)^a^	UK	Cross‐sectional	19	At‐risk (single mother)	—	7–13	—	WE	NR

*Note:* The first study reported was included in both systematic review and meta‐analysis.

Abbreviations: — = Information not retrieved; AF = Africa; AS = Asia‐Pacific; EA = Eastern‐European; LA = Latin American and Caribbean States; NA = North‐American; NR = not rated in the case of exclusive inclusion in the systematic review; WE = Western‐European.

^a^ Studies with duplicate samples only included in the systematic review.

^b^ According to United Nations Regions.

This review includes data from 2867 participants aged 6 up to 22, with a weighted average age of 12.25 years old (SD_w_ = 1.89) and a weighted average percentage of males being 43.43%. The majority (*n* = 1796, 62.60%; age M_w_ = 12.31_,_ SD_w_ = 1.33, 39.52%_w_ males) came from 18 community samples, followed by 891 (31.10%; age M_w =_ 12.66_,_ SD_w_ = 3.08, 47.70%_w_ males) participants in 19 at‐risk samples (mostly in a condition of adoption, 11/19 as 57.90%)—and only 180 (6.30%; age M_w =_ 9.78_,_ SD_w_ = 1.55, 44.97%_w_ males) participants in three clinical samples from two studies have a clinical diagnosis of anxiety in all cases.

Most studies involved participants with a Western‐European cultural background (*n* = 39, 75%), followed by eight North American studies (15.40%), three Eastern European (5.80) and only one in Latin America (1.90%) and Asia (1.90%) respectively.

### 3.2 Quality Assessment Results

**FIGURE 2:**
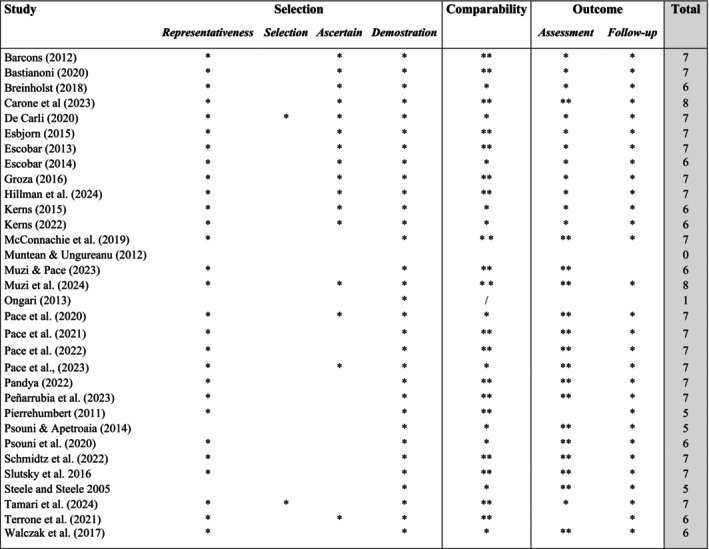
Results of the quality assessment with the Newcastle Ottawa Scale (NOS) for the studies included in the meta‐analysis.

The average score in the NOS scales for the 32 studies included in the meta‐analysis was 6.22, with a range of 0 to 8 and a mode of 7, indicating generally good quality of the studies included and low risk of biases. Specifically, most studies (*n* = 19; 59.37%) showed high quality receiving 7 or more, 11 (34.37%) studies had scores between 4 and 6 indicating moderate quality and only two studies scored 0 and 1 (5.93%) indicating high risk of biases. Figure 2 provides details about NOS evaluation results.

### 3.3 RQ1: Aggregate Distributions of Attachment Categories and Means Across Samples

Table 2 details distributions in the studies according to the type of sample and aggregate ones. Aggregate distributions were: 59% Secure (S) and 41% Insecure (of which 30% Ds, 9% P and 2% D) in the community samples (*k* = 11, *n* = 1080), 44% Secure (S) and 56% Insecure (36% Ds, 15% P and 5% D) in at‐risk samples (*k* = 13, *n* = 598) and 55% Secure (S) and 45% Insecure (44% Ds, 1% P, no D) in clinical samples with a diagnosis of anxiety disorder (*k* = 3, *n* = 175).

**TABLE 2:** Pooled categories rate and means in FFI patterns and overall coherence and secure base/safe haven scales.

		Patterns																		Domains							
		2‐way						3‐ or 4‐way												Coherence		Secure base/safe haven					
		Secure or F/S				Insecure		Ds				P/E				D				Overall		Parents^b^		Mother		Father	
Community samples	N	*n* ^a^	%	M	SD	*n* ^a^	%	*n* ^a^	%	M	SD	*n* ^a^	%	M	SD	*n* ^a^	%	M	SD	M	SD	M	SD	M	SD	M	SD
Breinholst et al. (2018)	*111*	*53*	48	—	—	*58*	52	*48*	43	—	—	*7*	7	—	—	*0*	0	—	—	—	—	—	—	—	—	—	—
Carone et al. (2023)_S1_	*55*	*30*	55	2.40	1.06	*25*	45	*16*	29	2.09	1.00	*9*	16	1.19	0.53	—	—	—	—	—	—	2.64	0.65	—	—	—	—
Carone et al. (2023)_S2_	*22*	*12*	55	2.45	1.19	*10*	45	*6*	28	2.21	1.23	*4*	18	1.17	0.46	—	—	—	—	—	—	2.71	0.78	—	—	—	—
Decarli et al. (2022)	*42*	*11*	27	2.20	1.10	*31*	74	*12*	29	2.78	0.90	*12*	9	2.78	1.05	*12*	29	2.18	1.29	—	—	—	—	—	—	—	—
Escobar et al. (2014)	*25*	*18*	72	1.56	2.33	*7*	28	*5*	20	—	—	*2*	8	—	—	—	—	—	—	—	—	—	—	—	—	—	—
Kerns et al. (2015)	*107*	—	—	—	—	—	—	—	—	—	—	—	—	—	—	—	—	—	—	2.68	0.73	2.30	0.83	2.37	0.81	2.23	0.81
Kerns et al. (2022)	*92*	—	—	—	—	—	—	—	—	—	—	—	—	—	—	—	—	—	—	2.48	0.73	2.14	0.71	2.22	0.74	2.07	0.69
McConnachie et al. (2020)_S1_	*30*	—	—	1.85	0.68	—	—	—	—	2.57	0.82	—	—	1.35	0.62	—	—	1.53	0.84	—	—	—	—	—	—	—	—
McConnachie et al. (2020)_S2_	*29*	—	—	2.02	0.60	—	—	—	—	2.05	0.76	—	—	1.95	0.77	—	—	1.72	0.81	—	—	—	—	—	—	—	—
McConnachie et al. (2020)_S3_	*38*	—	—	1.63	0.59	—	—	—	—	2.05	0.85	—	—	1.87	0.82			2.15	0.91	—	—	—	—	—	—	—	—
Pace et al. (2020)	*110*	*74*	67	2.85	0.80	*36*	33	*25*	23	1.80	0.82	*8*	7	1.42	0.58	*3*	3	1.14	0.39	3.03	0.58	2.50	0.78	2.68	0.81	2.32	0.76
Pace et al. (2021)	*112*	*69*	62	3.04	2.60	*43*	38	*20*	18	2.85	2.78	*22*	20	2.21	2.79	*1*	1	1.91	0.81	2.81	0.67	2.33	0.95	2.47	0.89	2.19	1.01
Pace et al. (2023)	*102*	*83*	81	3.22	0.77	*19*	19	*17*	17	1.59	0.76	*1*	1	1.17	0.35	*1*	1	1.05	0.24	3.39	0.58	2.87	0.85	2.92	0.82	2.82	0.88
Peñarrubia et al. (2023)	*38*	*31*	82	2.97	0.68	*7*	18	*3*	8	1.63	0.82	*2*	5	1.45	0.69	*2*	5	1.13	0.58	2.92	0.71	2.44	0.91	2.74	1.01	2.13	0.81
Psouni and Apetroaia (2014)	*120*	*63*	53	—	—	*57*	48	*46*	38	—	—	*8*	7	—	—	*3*	3	—	—	—	—	—	—	—	—	—	—
Psouni et al. (2020)	*341*	*192*	56	2.46	0.53	*149*	44	*125*	37	2.28	1.28	*17*	5	1.24	0.35	*7*	2	1.09	0.25	2.58	0.49	2.21	0.75	2.45	0.75	1.97	0.81
Steele and Steele (2005)	*55*	—	—	—	—	—	—	—	—	—	—	—	—	—	—	—	—	—	—	2.14	0.70	—	—	—	—	—	—
Tamari et al. (2024)	*108*	—	—	—	—	—	—	—	—	—	—	—	—	—	—	—	—	—	—	2.40	0.87	2.56	0.93	2.69	1.02	2.42	0.84
Terrone et al. (2021)	*178*	—	—	2.74	0.81	—	—	—	—	1.95	0.88	—	—	1.45	0.67	—	—	1.26	0.63	2.74	0.76	2.35	0.78	2.52	0.83	2.17	0.73
**Pooled weighted total**		*636*	**59**	**2.43**	**0.94**	*442*	**41**	*323*	**30**	**2.11**	**1.13**	*92*	**9**	**1.53**	**0.76**	*29*	**2**	**1.45**	**0.51**	**2.70**	**0.65**	**2.44**	**0.80**	**2.55**	**0.82**	**2.25**	**0.81**
**At‐risk samples**	***N***	***n***	**%**	**M**	**SD**	***n*** ^***a***^	***%***	***n***	**%**	**M**	**SD**	***n***	**%**	**M**	**SD**	***n***	**%**	**M**	**SD**	**M**	**SD**	**M**	**SD**	**M**	**SD**	**M**	**SD**
Barcons et al. (2012)	*116*	*70*	60	—	—	*46*	40	30	26	—	—	*14*	12	—	—	*2*	2	—	—	2.88	0.79	2.40	0.71	—	—	—	—
Bastianoni et al. (2020)	*13*	*7*	54	2.58	1.00	*6*	46	4	31	1.69	0.85	*1*	8	1.50	1.50	*1*	7	1.38	0.87	—	—	—	—	—	—	—	—
Esbjørn et al. (2015)	*34*	*9*	27	1.97	0.97	*25*	74	17	50	3.21	0.96	*2*	6	1.62	0.92	*6*	18	1.68	0.94	—	—	—	—	—	—	—	—
Escobar et al. (2013)	*44*	*22*	50	1.50	1.31	*22*	50	16	37	—	—	*6*	13	—	—	*0*	0	—	—	—	—	—	—	—	—	—	—
Escobar et al. (2014)	*25*	*8*	32	—	—	*17*	68	13	52	—	—	*4*	16	—	—	—	—	—	—	—	—	—	—	—	—	—	—
Groza (2016)	*63*	*34*	54	—	—	*29*	46	—	—	—	—	—	—	1.29	0.71	—	—	—	—	—	—	—	—	—	—	—	—
Hillman et al. (2024)	*70*	—	—	2.55	1.19	—	—	—	—	2.09	1.16	—	—	1.76	0.83	—	—	1.64	0.97	2.49	1.02	—	—	—	—	—	—
Muntean and Ungureanu (2012)	*40*	—	—	—	—	—	—	—	—	—	—	—	—	1.17	0.48	—	—	—	—	—	—	—	—	—	—	—	—
Muzi and Pace et al. (2023)	*46*	*9*	20	1.70	0.74	*37*	80	18	39	2.36	0.87	*10*	22	1.00	0	*9*	20	1.79	0.83	2.34	0.44	1.57	0.67	1.60	0.70	1.54	0.65
Muzi et al. (2024)	*36*	*16*	44	2.29	0.95	*20*	56	16	44	2.07	0.96	*2*	6	1.49	0.67	*2*	6	1.22	0.48	—	—	—	—	—	—	—	—
Ongari et al. (2013)	*4*	*1*	25	2.00	1.41	*3*	75	21	50	2.75	0.86	*0*	0	2.44	0.47	*1*	25	1.75	0.96	2.50	1.00	—	—	—	—	—	—
Pace et al. (2022)	*79*	*51*	65	2.68	0.78	*28*	35	21	26	2.03	0.41	*4*	5	2.46	0.39	*3*	4	1.27	0.55	2.86	0.57	2.48	0.67	2.53	0.84	2.44	0.85
Pandya (2023)_S1_	*43*	—	—	1.23	0.13	—	—	—	—	2.32	0.32	—	—	1.50	0.73	—	—	2.38	0.45	—	—	1.78	0.84	—	—	—	—
Pandya (2023)_S2_	*43*	—	—	1.25	0.13	—	—	—	—	2.34	1.08	—	—	1.56	0.84	—	—	2.40	0.43	—	—	1.75	0.44	—	—	—	—
Peñarrubia et al. (2023)	*29*	*12*	41	2.33	0.71	*17*	59	10	35	2.07	0.98	*4*	14	2.70	0.96	*3*	10	1.40	0.77	2.33	0.71	2.04	0.43	2.17	0.93	1.90	0.72
Pierrehumbert et al. (2011)	*32*	—	—	2.53	1.24	—	—	—	—	2.00	0.99	—	—	—	—	—	—	1.25	0.57	3.47	0.61	3.39	0.82	3.53	0.70	3.24	0.75
Schmidt et al. (2022)	*90*	*9*	10	1.40	0.79	*81*	90	42	47	2.66	0.85	*35*	39	—	—	*4*	4	—	—	—	—	1.50	0.72	1.67	0.69	1.33	0.60
Slutsky et al. (2016)	*19*	*12*	63	—	—	*7*	37	5	26	—	—	*2*	11	1.50	1.50	*0*	0	1.28	0.56	—	—	—	—	—	—	—	—
**Pooled weighted total**		*260*	**44**	**1.93**	**0.81**	*338*	**56**	*194*	**36**	**2.30**	**0.88**	*84*	**15**	**1.73**	**0.73**	*31*	**5**	**1.58**	**0.66**	**2.70**	**0.72**	**2.04**	**0.67**	**2.21**	**0.76**	**1.94**	**0.71**
**Clinical samples**	***N***	***n***	***%***	**M**	**SD**	***n*** ^***a***^	***%***	***n***	***%***	**M**	**SD**	***n***	***%***	**M**	**SD**	***n***	**%**	**M**	**SD**	**M**	**SD**	**M**	**SD**	**M**	**SD**	**M**	**SD**
Breinholst et al. (2018)	*111*	*60*	54	—	—	*51*	46	*49*	44	—	—	*1*	1,1	—	—	*0*	0	—	—	—	—	—	—	—	—	—	—
Walczak et al. (2017)_S1_	*32*	*18*	56	2.59	1.10	*14*	44	*14*	44	2.72	1.11	*0*	0	1.16	0.37	*0*	0	1.16	0.37	2.41	0.56	—	—	—	—	—	—
Walczak et al. (2017)_S2_	*32*	*18*	57	2.57	1.17	*14*	43	*14*	43	2.76	1.19	*0*	0	1.05	0.23	*0*	0	1.08	0.28	2.46	0.65	—	—	—	—	—	—
**Pooled weighted total**		*96*	**55**	**2.58**	**1.13**	*79*	**45**	*77*	**44**	**2.74**	**1.15**	*1*	**1**	**1.11**	**0.30**	*0*	**0**	**1.12**	**0.32**	**2.44**	**0.61**	—	—	—	—	—	—

^a^ Frequency within the category.

^b^ Average mean of mother and father scores.

As expected, community participants obtained mostly secure classifications and insecure ones prevailed in at‐risk samples. Contrary to expectations, the few clinical participants with a diagnosis of anxiety displayed normative levels of secure classifications.

#### 3.3.1 Computation of Pooled Weighted Means as ES

**FIGURE 3:**
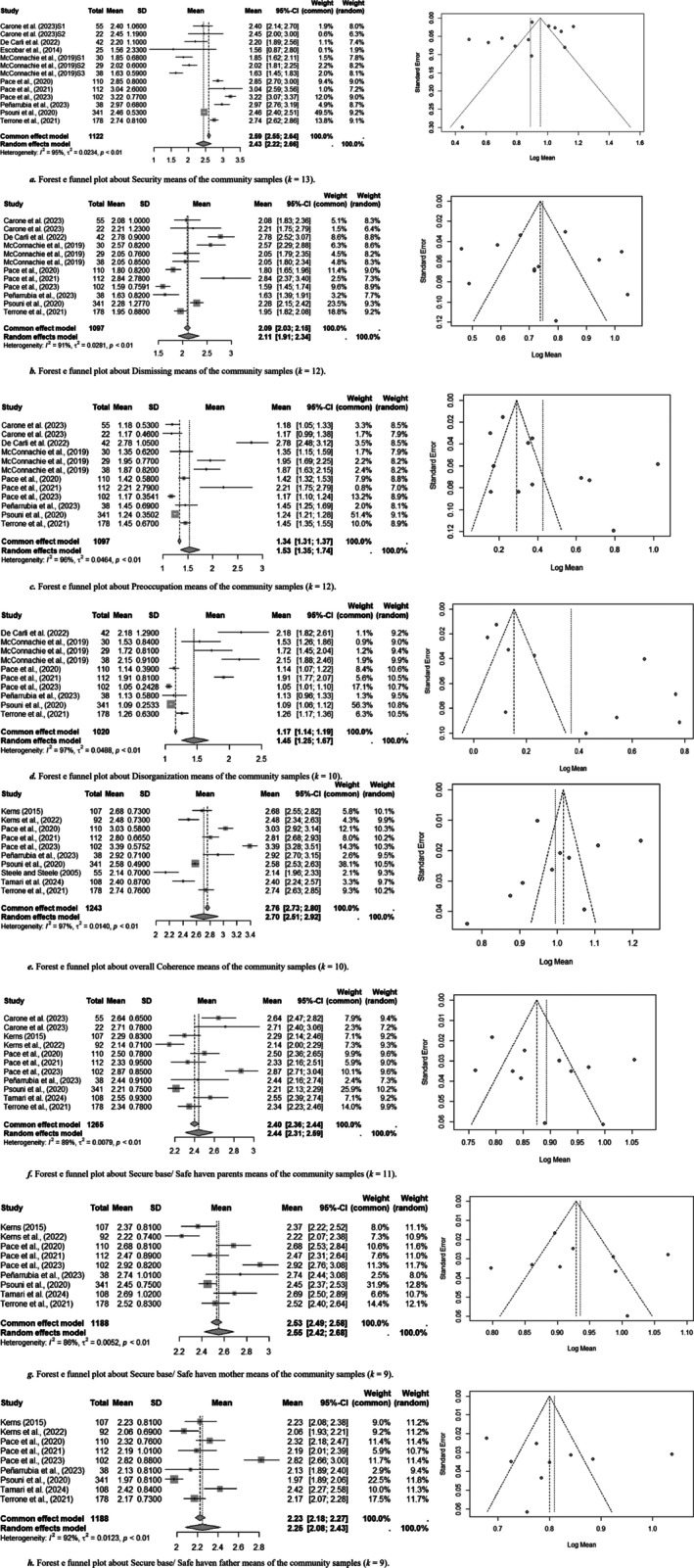
Computation on weighted means in the community subgroups, with forest and funnel plots for each scale. (a) Forest e funnel plot about Security means of the community samples (k‐13). (b) Forest e funnel plot about Dismissing means of the community samples (*k* = 12). (c) Forest e funnel plot about Preoccupation means of the community samples (*k* = 12). (d) Forest e funnel plot about Disorganization means of the community samples (*k* = 10). (e) Forest e funnel plot about overall Coherence means of the community samples (*k* = 10). (f) Forest e funnel plot about Secure base/Safe Haven parents means of the community samples (*k* = 11). (g) Forest e funnel plot about Secure base/Safe Haven mother means of the community samples (*k* = 9). (h) Forest e funnel plot about Secure base/Safe Haven father means of the community samples (*k* = 9).

**FIGURE 4:**
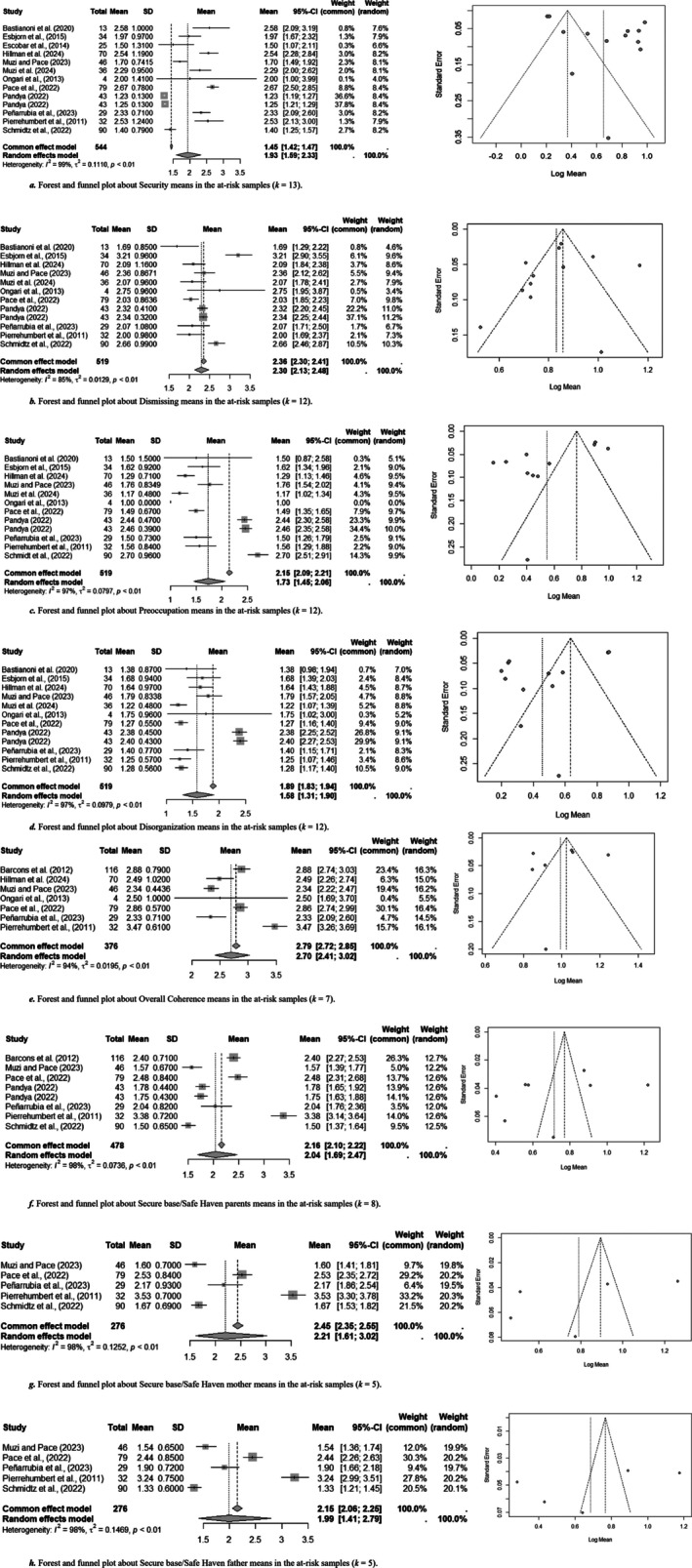
Computation on weighted means in nthe at‐risk samples, with forest and funnel plots for each scale. (a) Forest e funnel plot about Security means in the at‐risk samples (*k* = 13). (b) Forest e funnel plot about Dismissing meands in the at‐risk samples (*k* = 12). (c) Forest e funnel plot about Preoccupation means in the at‐risk samples (*k* = 12). (d) Forest e funnel plot about Disorganization means in the at‐risk samples (*k* = 12). (e) Forest e funnel plot about Overall Coherence means in the at‐risk samples (*k* = 7). (f) Forest e funnel plot about Secure base/Safe Haven parents means in the at‐risk samples (*k* = 8). (f) Forest e funnel plot about Secure base/Safe Haven mother means in the at‐risk samples (*k* = 5). (f) Forest e funnel plot about Secure base/Safe Haven father means in the at‐risk samples (*k* = 5).

Table 2 also reports on the weighted averages and SD obtained by pooling means of different samples within the same group. Table 3 reports results of heterogeneity and moderators for all research questions and forest and funnel plots with number of samples (*k*), pooled sample sizes and heterogeneity values for all FFI scales are reported in Figure 3 for community group and Figure 4 for at‐risk ones. Overall, there was significant heterogeneity for all mean results in both groups, suggesting caution in interpreting results.

**TABLE 3:** Synthesis of effect sizes heterogeneity and moderators for the meta‐analysis on the Friends and Family Interview.

Objective	Sample type (weighted means)		Relationships (standardized *r*) with						
			Psychopathological symptoms						Verbal IQ
FFI scale	Community	At‐risk	Total	Internal	External	Attentional	Social	Thought	
Secure									
Heterogeneity									
*τ* ^*2*^	0.02**	0.11**	0	0.01	0	0	0	0	0.05
*I* ^*2*^	95%	99%	0%	26%	0%	0%	0%	0%	76%
*Q*	—	—	3.30	8.10	6.40	3.30	4.6	5.00	24.3**
Publication bias (Eggers' test z)	—	—	0.42	−2.44*	−1.23	−0.43	−0.94	−1.93	−0.78
Moderators									
Age	n.s.	n.s.	n.s.	n.s.	n.s.	n.s.	n.s.	n.s.	−0.17***
Gender (male %)	n.s.	n.s.	n.s.	n.s.	n.s.	n.s.	n.s.	n.s.	−0.04**
Study quality	n.s.	n.s.	n.s.	n.s.	n.s.	n.s.	n.s.	n.s.	n.s.
Sample type^a^	n.s.	—	n.s.	n.s.	n.s.	n.s.	n.s.	n.s.	0.59**
Dismissing									
Heterogeneity									
*τ* ^*2*^	0.03**	0.01**	0.13	0.01	0.26	0	0.01	0.13	0.01
*I* ^*2*^	91%	85%	90%	45%	95%	0%	51%	90%	25%
*Q*	—	—	74.90**	11.30	143.70**	6.20	12.20	75.20	7.70
Publication bias (Eggers' test z)	—	—	1.11	−0.33	1.65	−0.40	0.04	1.13	0.56
Moderators									
Age	n.s.	n.s.	n.s.	n.s.	n.s.	n.s.	n.s.	n.s.	n.s.
Gender (male %)	n.s.	n.s.	n.s.	n.s.	n.s.	n.s.	n.s.	n.s.	n.s.
Study quality	n.s.	n.s.	n.s.	n.s.	n.s.	n.s.	n.s.	n.s.	n.s.
Sample type^a^	n.s.	—	n.s.	n.s.	1.11**	n.s.	n.s.	n.s.	n.s.
Preoccupied									
Heterogeneity									
*τ* ^*2*^	0.05**	0.08**	0.01	0.03	0.01	0.01	0	0.01	0.01
*I* ^*2*^	96%	97%	11%	66%	34%	4%	0%	46%	34%
*Q*	—	—	6.80	16.10	9.00	5.70	4.60	11.20	8.60
Publication bias (Eggers' test z)	—	—	0.77	0.74	−0.87	1.70	0.96	0.33	0.63
Moderators									
Age	—	—	n.s.	n.s.	n.s.	n.s.	n.s.	n.s.	n.s.
Gender (male %)	n.s.	n.s.	n.s.	n.s.	n.s.	n.s.	n.s.	n.s.	n.s.
Study quality	—	—	n.s.	n.s.	n.s.	n.s.	n.s.	n.s.	n.s.
Sample type^a^	n.s.	—	n.s.	n.s.	n.s.	0.28	n.s.	n.s.	n.s.
Disorganized									
Heterogeneity									
*τ* ^*2*^	0.05**	0.10**	0	0	0	0	0.01	0	0.03
*I* ^*2*^	97%	97%	0%	0%	0%	0%	26.3%	0%	60%
*Q*	—	—	1.30	3.60	1.70	1.70	6.40	0.50	13.3*
Publication bias (Eggers' test z)	—	—	0.74	0.66	0.81	−0.19	0.11	0.18	0.14
Moderators									
Age	n.s.	n.s.	n.s.	n.s.	n.s.	n.s.	n.s.	n.s.	n.s.
Gender (male %)	n.s.	n.s.	n.s.	n.s.	n.s.	n.s.	n.s.	n.s.	n.s.
Study quality	n.s.	n.s.	n.s.	n.s.	n.s.	n.s.	n.s.	n.s.	n.s.
Sample type^a^	n.s.	—	n.s.	n.s.	n.s.	n.s.	n.s.	n.s.	−0.36*
Overall coherence									
Heterogeneity									
*τ* ^*2*^	0.01**	0.02**	0.01	0.01	0.01	0	0	0	0.02
*I* ^*2*^	97%	94%	4.5%	36%	21%	0%	0%	0%	60%
*Q*	—	—	6.00	9.50	8.4	3.8	5.4	4.7	15.4*
Publication bias (Eggers' test z)	—	—	−1.24	−2.12*	−0.92	−0.16	−0.93	−1.97	−0.20
Moderators									
Age	n.s.	n.s.	n.s.	n.s.	n.s.	n.s.	n.s.	n.s.	n.s.
Gender (male %)	n.s.	n.s.	n.s.	n.s.	n.s.	n.s.	n.s.	n.s.	−0.03*
Study quality	n.s.	n.s.	n.s.	n.s.	n.s.	n.s.	n.s.	n.s.	n.s.
Sample type^a^	n.s.	—	n.s.	n.s.	n.s.	n.s.	n.s.	n.s.	0.51**
SB/SH Mother									
Heterogeneity									
*τ* ^*2*^	0.01**	0.12**	0	0	0.01	0	0.01	0.01	0.03
*I* ^*2*^	86%	98%	0%	0%	26.5%	0%	32.5%	18%	59%
*Q*			4.50	3.30	7.80	3.1	8.00	6.70	14.1*
Publication bias (Eggers' test z)	—	—	−0.78	−1.38	−1.12	0.47	−0.62	−1.04	−0.55
Moderators									
Age	n.s.	n.s.	n.s.	n.s.	n.s.	n.s.	n.s.	n.s.	n.s.
Gender (male %)	n.s.	n.s.	n.s.	n.s.	n.s.	n.s.	n.s.	n.s.	n.s.
Study quality	n.s.	n.s.	n.s.	n.s.	n.s.	n.s.	n.s.	n.s.	n.s.
Sample type^a^	n.s.	—	n.s.	n.s.	n.s.	n.s.	n.s.	n.s.	n.s.
SB/SH father									
Heterogeneity									
*τ* ^*2*^	0.01**	0.15**	0.02	0	0.03	0.01	0.01	0.01	0.03
*I* ^*2*^	92%	98%	52.5%	0%	63%	27%	37%	22%	67%
*Q*	—	—	12.90	2.90	16.70*	9.00	9.60	7.80	18.3**
Publication bias (Eggers' test z)	—	—	0.58	0.01	0.67	1.47	0.80	0.57	−0.34
Moderators									
Age	n.s.	n.s.	n.s.	n.s.	n.s.	n.s.	n.s.	n.s.	−0.12*
Gender (male %)	n.s.	n.s.	0.02**	n.s.	0.02*	0.01*	0.01*	0.01*	−0.04***
Study quality	n.s.	n.s.	0.96**	n.s.	n.s.	0.74*	n.s.	0.77*	n.s.
Sample type^a^	n.s.	—	−0.35**	n.s.	n.s.	n.s.	−0.25*	n.s.	0.56* ^yyyy^

*Note:* — = Not Applicable or impossible to calculate. Moderators β coefficients are reported only if significant.

^a^ Community = 0, at‐risk = 2.

* *p* < 0.05.

** *p* < 0.01.

*** *p* < 0.001.

### 3.4 RQ2: Potential Moderators of FFI Weighted Means

Meta‐regressions with age and study quality as moderators of Preoccupation results could not be performed due to too low variance in both groups. groupgroupAs shown in Table 3 (above), no moderation effects were detected for participants' gender and age or study quality, in the community group (Q between 0.01 and 0.85, all *p* > 0.36) or at‐risk (Q between 0.02 and 2.41, all *p* > 0.120) groups. In both groups, no moderation effects were revealed for SB/SH parents' scales, with Q between 0.01 and 0.45 in the community group (all *p* > 0.616) and Q between 0.25 and 2.79 in at‐risk group (all *p* > 0.095).

### 3.5 RQ3: Differences Related to Participants' Sample Type and Cultural Background

Because only two samples from the same study reported means of clinical participants, weighted aggregated data are reported in Table 2 but not meta‐analysed and comparisons with the clinical group were performed only regarding the pooled percentage distribution.

Similarly, a lack of data from at least two samples from two different studies with cultural backgrounds other than Western European impeded the computation of ES and consequent analysis of this variable and aggregated data are reported below (Section 3.4.4) only for descriptive purposes.

#### 3.5.1 Type of Sample

##### 3.5.1.1 Comparisons of Categories According to Type of Sample

The *community* group (*K* = 11, *N* = 1080) was used as the normative reference group against which the other two non‐community subgroups were compared. These groups included participants *at risk* of developing a mental disorder without a formal diagnosis and a *clinical* subgroup diagnosed with a psychiatric condition (see Section 2.4 for details). Both groups were considered to be at greater risk of insecurity than the community group.

###### 3.5.1.1.1 Community vs. At‐Risk

Odd ratios (OR) and Relative Risk (RR) analyses revealed that individuals in the at‐risk samples (*k* = 13, *n* = 598) were less likely to be classified as Secure (−37.50%; logOR = −0.60 [−1.17 to −0.04], SE = 0.29, variance = 0.08) and had a higher risk to receive an Insecure classification (logRR = 0.32, SE = 0.05; RR = 1.38 [1.25 to 1.52], RD = 0.15 [0.10 to 0.20], *p* < 0.001) compared to community participants.

Specifically, at‐risk participants had higher odds and risk of receiving a Preoccupied (Odds: 39.40%; logOR = 0.65 [−0.21 to 1.52], SE = 0.44, variance = 0.20; Risk: logRR = 0.50, SE = 0.14; RR = 1.64 [1.25–2.17], RD = 0.05 [0.02–0.09], *p* < 0.001) or Disorganized (Odds: 41.86%; logOR = 0.72 [−0.69 to 2.14], SE = 0.72, variance = 0.52; Risk: logRR = 0.62, SE = 0.25; RR = 1.86 [1.14–3.05], RD = 0.02 [0.01–0.04], *p* = 0.012) classification than the community ones. However, they have higher odds to receive a Dismissing classification (21.26%; logOR = 0.27 [−0.63 to 1.17], SE = 0.29, variance = 0.08), but not a substantial higher risk (logRR = 0.08, SE = 0.07; RD = 0.02 [−0.02 to 0.07], *p* = 0.293).

Additionally, regarding within‐group differences in insecure categories, community participants were most likely to be assigned in the Dismissing insecure classification (63.93%; logOR = −0.12 [−1.73 to 1.49], SE = 0.82, variance = 0.67). Instead, at‐risk participants have higher odds to be classified as Preoccupied rather than Dismissing (−104.08%; logOR = −0.51 [−1.98 to 0.96], SE = 0.75, variance = 0.56) and Disorganized (−63.93%; logOR = −0.39 [−1.34 to 0.56], SE = 0.48, variance = 0.23).

###### 3.5.1.1.2 Community vs. Clinical

For participants in the *clinical samples* (*k* = 3, *n* = 175), it was not possible to compute OR and RR values for the Disorganized category because no individuals in this subgroup were classified in that category (*n* = 0, 0%).

No significant difference emerged between community and clinical participants in the odds of receiving a Secure classification over any Insecure ones (Odds: logOR = −0.16 [−0.72 to 0.40], SE = 0.28, variance = 0.08; logRR = 0.07, SE = 0.07; RD = 0.04 [−0.04 to 0.12], *p* = 0.302). However, considering the single Insecure category type, the clinical group had higher odds and risk of being classified as Dismissing than communities (Odds: 30.55%; logOR = 0.44 [−0.13 to 1.01], SE = 0.29, variance = 0.08; Risk: logRR = 0.38, SE = 0.10; RR = 1.46 [1.21 to 1.78], RD = 0.14 [0.06 to 0.22], *p* < 0.001). Moreover, clinical participants had significantly lower odds and risk of being classified as Preoccupied (Odds: −178.12%; logOR = −2.28 [−4.36 to −0.20], SE = 1.06, variance = 1.13; Risk: logRR = −2.70, SE = 1.00; RR = 0.07 [0.01 to 0.48], RD = −0.08 [−0.10 to −0.06], *p* < 0.001).

Additionally, regarding within‐group likelihood of receiving insecure category types, the likelihood of being categorized as Dismissing over Preoccupied was higher in the clinical subgroup than in the community one (74.68%; logOR = 2.95 [0.88 to 5.03], SE = 1.06, variance = 0.12).

###### 3.5.1.1.3 Clinical vs. At‐Risk

Clinical participants had slightly higher odds of being classified as Secure than at‐risk ones, approaching significance but not reaching it (30.50%; logOR = 0.44 [−0.11 to 1.00], SE = 0.28, variance = 0.08).

Although no significant difference emerged between clinical and at‐risk groups in the odds of receiving a Dismissing classification (logOR = 0.17 [−0.39 to 0.79], SE = 0.28, variance = 0.08), the clinical group showed a higher risk to receive this category (logRR = 0.30, SE = 0.10; RR = 1.36 [1.11 to 1.66], RD = 0.05 [0.02 to 0.09], *p* = 0.004). Conversely, clinical participants had substantially lower odds and risk of receiving a Preoccupied classification than the at‐risk counterpart (Odds: −152.35%; logOR = −2.91 [−4.95 to −0.87], SE = 1.04, variance = 1.08; Risk: logRR = −3.20, SE = 1.00; RR = 0.04 [0.01 to 0.29], RD = −0.13 [−0.16 to −0.10], *p* < 0.001).

The comparison on within‐group likelihood of receiving insecure category types revealed higher likelihood of being categorized as Dismissing over Preoccupied in the clinical subgroup than in the at‐risk one (74.81%; logOR = 2.97 [0.90 to 5.04], SE = 1.05, variance = 1.11).

##### 3.5.1.2 Comparisons of FFI Patterns, Coherence and SB/SH Scores

SMD analyses indicated that participants in the at‐risk samples (*n* = 544) scored significantly lower (*n* = 1122) on the Secure scale, with a medium effect size (Hedge's g = −0.55 [−0.66 to −0.45], SE = 0.05, variance = 0.01) and slightly higher on the Disorganization scale, corresponding to a small effect (Hedge's g = 0.22 [0.11–0.32], SE = 0.05, variance = 0.01; n_community_ = 102, n_at‐risk_ = 519) than community participants.

By contrast, differences between groups were non‐significant on the Dismissing (Hedge's g = 0.18 [0.07 to 0.28], SE = 0.05, variance = 0.01) and Preoccupation (Hedge's g = 0.13 [0.03 to 0.24], SE = 0.05, variance = 0.01) scales.

Turning to the other subscales, no meaningful difference was observed in Overall coherence (Hedge's g = 0.00 [−0.11 to 0.11], SE = 0.06, variance = 0.01) between community and at‐risk participants (*n* = 376). However, the at‐risk group scored lower on the combined parent safe haven/secure base scale (Hedge's g = −0.34 [−0.44 to −0.23], SE = 0.05, variance = 0.01; n_community_ = 1265, n_at‐risk_ = 478) than the community group (*n* = 1243). Similar patterns were observed for mother and father subscales when examined separately: Hedge's g_mother_ = −0.46 [−0.59 to −0.32], SE = 0.07, variance = 0.01 and Hedge's g_father_ = −0.39 [−0.52 to −0.26], SE = 0.07, variance = 0.01 (n_community_ = 1188, n_at‐risk_ = 276).

#### 3.5.2 Cultural Background

Table 4 reports the available data aggregate for cultural background, regardless of the sample type.

**TABLE 4:** Pooled categories rate and means in FFI patterns and overall coherence and secure base/safe haven scales according to the cultural background.

		Patterns																		Coherence		Secure base/safe haven					
		Insecure		Secure or F/S				Ds				P/E				D/U				Overall		Parents^b^		Mother		Father	
Western European samples	*N*	*n*	%	*n* ^*a*^	%	M	SD	*n* ^*a*^	%	M	SD	*n* ^*a*^	%	M	SD	*n* ^*a*^	%	M	SD	M	SD	M	SD	M	SD	M	SD
Barcons et al. (2012)	*116*	*46*	40	*70*	60	—	—	30	26	—	—	*14*	12	—	—	*2*	2	—	—	2.88	0.79	2.40	0.71	—	—	—	—
Bastianoni et al. (2020)	*13*	*6*	46	*7*	54	2.58	1.00	4	31	1.69	0.85	*1*	8	1.50	1.50	*1*	7	1.38	0.87	—	—	—	—	—	—	—	—
Breinholst et al. (2018)_S1_	*111*	*58*	52	*53*	48	—	—	*48*	43	—	—	*7*	7	—	—	*0*	0	—	—	—	—	—	—	—	—	—	—
Breinholst et al. (2018)_S2_	*111*	*51*	46	*60*	54	—	—	*49*	44	—	—	*1*	1.1	—	—	*0*	0	—	—	—	—	—	—	—	—	—	—
Carone et al. (2023)_S1_	*55*	*25*	45	*30*	55	2.40	1.06	*16*	29	2.09	1.00	*9*	16	1.19	0.53	—	—	—	—	—	—	2.64	0.65	—	—	—	—
Carone et al. (2023)_S2_	*22*	*10*	45	*12*	55	2.45	1.19	*6*	28	2.21	1.23	*4*	18	1.17	0.46	—	—	—	—	—	—	2.71	0.78	—	—	—	—
Decarli et al. (2022)	*42*	*31*	74	*11*	27	2.20	1.10	*12*	29	2.78	0.90	*12*	9	2.78	1.05	*12*	29	2.18	1.29	—	—	—	—	—	—	—	—
Hillman et al. (2024)	*70*	—	—	—	—	2.55	1.19	—	—	2.09	1.16	—	—	1.76	0.83	—	—	1.64	0.97	2.49	1.02	—	—	—	—	—	—
McConnachie et al. (2020)_S1_	*30*	—	—	—	—	1.85	0.68	—	—	2.57	0.82	—	—	1.35	0.62	—	—	1.53	0.84	—	—	—	—	—	—	—	—
McConnachie et al. (2020)_S2_	*29*	—	—	—	—	2.02	0.60	—	—	2.05	0.76	—	—	1.95	0.77	—	—	1.72	0.81	—	—	—	—	—	—	—	—
McConnachie et al. (2020)_S3_	*38*	—	—	—	—	1.63	0.59	—	—	2.05	0.85	—	—	1.87	0.82			2.15	0.91	—	—	—	—	—	—	—	—
Muzi and Pace et al. (2023)	*46*	*37*	80	*9*	20	1.70	0.74	18	39	2.36	0.87	*10*	22	1.00	0	*9*	20	1.79	0.83	2.34	0.44	1.57	0.67	1.60	0.70	1.54	0.65
Muzi et al. (2024)	*36*	*20*	56	*16*	44	2.29	0.95	16	44	2.07	0.96	*2*	6	1.49	0.67	*2*	6	1.22	0.48	—	—	—	—	—	—	—	—
Ongari et al. (2013)	*4*	*3*	75	*1*	25	2.00	1.41	21	50	2.75	0.86	*0*	0	2.44	0.47	*1*	25	1.75	0.96	2.50	1.00	—	—	—	—	—	—
Pace et al. (2020)	*110*	*36*	33	*74*	67	2.85	0.80	*25*	23	1.80	0.82	*8*	7	1.42	0.58	*3*	3	1.14	0.39	3.03	0.58	2.50	0.78	2.68	0.81	2.32	0.76
Pace et al. (2021)	*112*	*43*	38	*69*	62	3.04	2.60	*20*	18	2.85	2.78	*22*	20	2.21	2.79	*1*	1	1.91	0.81	2.81	0.67	2.33	0.95	2.47	0.89	2.19	1.01
Pace et al. (2022)	*79*	*28*	35	*51*	65	2.68	0.78	21	26	2.03	0.41	*4*	5	2.46	0.39	*3*	4	1.27	0.55	2.86	0.57	2.48	0.67	2.53	0.84	2.44	0.85
Pace et al. (2023)	*102*	*19*	19	*83*	81	3.22	0.77	*17*	17	1.59	0.76	*1*	1	1.17	0.35	*1*	1	1.05	0.24	3.39	0.58	2.87	0.85	2.92	0.82	2.82	0.88
Peñarrubia et al. (2023)_S1_	*38*	*7*	18	*31*	82	2.97	0.68	*3*	8	1.63	0.82	*2*	5	1.45	0.69	*2*	5	1.13	0.58	2.92	0.71	2.44	0.91	2.74	1.01	2.13	0.81
Peñarrubia et al. (2023)_S2_	*29*	*17*	59	*12*	41	2.33	0.71	10	35	2.07	0.98	*4*	14	2.70	0.96	*3*	10	1.40	0.77	2.33	0.71	2.04	0.43	2.17	0.93	1.90	0.72
Psouni and Apetroaia (2014)	*120*	*57*	48	*63*	53	—	—	*46*	38	—	—	*8*	7	—	—	*3*	3	—	—	—	—	—	—	—	—	—	—
Psouni et al. (2020)	*341*	*149*	44	*192*	56	2.46	0.53	*125*	37	2.28	1.28	*17*	5	1.24	0.35	*7*	2	1.09	0.25	2.58	0.49	2.21	0.75	2.45	0.75	1.97	0.81
Tamari et al. (2024)	*108*	—	—	—	—	—	—	—	—	—	—	—	—	—	—	—	—	—	—	2.40	0.87	2.56	0.93	2.69	1.02	2.42	0.84
Terrone et al. (2021)	*178*	—	—	—	—	2.74	0.81	—	—	1.95	0.88	—	—	1.45	0.67	—	—	1.26	0.63	2.74	0.76	2.35	0.78	2.52	0.83	2.17	0.73
Walczak et al. (2017)_S1_	*32*	*14*	44	*18*	56	2.59	1.10	*14*	44	2.72	1.11	*0*	0	1.16	0.37	*0*	0	1.16	0.37	2.41	0.56	—	—	—	—	—	—
Walczak et al. (2017)_S2_	*32*	*14*	43	*18*	57	2.57	1.17	*14*	43	2.76	1.19	*0*	0	1.05	0.23	*0*	0	1.08	0.28	2.46	0.65	—	—	—	—	—	—
Schmidt et al. (2022)	*90*	*81*	90	*9*	10	1.40	0.79	42	47	2.66	0.85	*35*	39	—	—	*4*	4	—	—	—	—	1.50	0.72	1.67	0.69	1.33	0.60
Slutsky et al. (2016)	*19*	*7*	37	*12*	63	—	—	5	26	—	—	*2*	11	1.50	1.50	*0*	0	1.28	0.56	—	—	—	—	—	—	—	—
Eastern‐European samples	*N*	*n*	%	*n*	%	M	SD	*n*	%	M	SD	*n*	%	M	SD	*n*	%	M	SD	M	SD	M	SD	M	SD	M	SD
Groza (2016)	*63*	*29*	46	*34*	54	—	—	—	—	—	1.16	—	—	1.29	0.71	—	—	—	—	—	—	—	—	—	—	—	—
Muntean and Ungureanu (2012)	*40*	—	—	—	—	—	—	—	—	—	—	—	—	1.17	0.48	—	—	—	—	—	—	—	—	—	—	—	—
Pierrehumbert et al. (2011)	*32*	—	—	—	—	2.53	1.24	—	—	2.00	0.99	—	—	—	—	—	—	1.25	0.57	3.47	0.61	3.39	0.82	3.53	0.70	3.24	0.75
North‐American samples	*N*	*n*	%	*n*	%	M	SD	*n*	%	M	SD	*n*	%	M	SD	*n*	%	M	SD	M	SD	M	SD	M	SD	M	SD
Esbjørn et al. (2015)	*34*	*25*	74	*9*	27	1.97	0.97	17	50	3.21	0.96	*2*	6	1.62	0.92	*6*	18	1.68	0.94	—	—	—	—	—	—	—	—
Kerns et al. (2015)	*107*	—	—	—	—	—	—	—	—	—	—	—	—	—	—	—	—	—	—	2.68	0.73	2.30	0.83	2.37	0.81	2.23	0.81
Kerns et al. (2022)	*92*	—	—	—	—	—	—	—	—	—	—	—	—	—	—	—	—	—	—	2.48	0.73	2.14	0.71	2.22	0.74	2.07	0.69
Steele and Steele (2005)	*55*	—	—	—	—	—	—	—	—	—	—	—	—	—	—	—	—	—	—	2.14	0.70	—	—	—	—	—	—
Latin American samples	*N*	*n*	*%*	*n*	*%*	M	SD	*n*	*%*	M	SD	*n*	*%*	M	SD	*n*	%	M	SD	M	SD	M	SD	M	SD	M	SD
Escobar et al. (2013)	*44*	*22*	50	*22*	50	1.50	1.31	*16*	37	—	—	*6*	13	—	—	*0*	0	—	—	—	—	—	—	—	—	—	—
Escobar et al. (2014) S2	*25*	*17*	68	*8*	32	—	—	*13*	52	—	—	*4*	16	—	—	—	—	—	0.97	—	—	—	—	—	—	—	—
Escobar et al. (2014) S2	*25*	*7*	28	*18*	72	1.56	2.33	*5*	20	—	—	*2*	8	—	—	—	—	1.53	0.84	—	—	—	—	—	—	—	—
Asiatic samples																											
Pandya (2023)_S1_	*43*	—	—	—	—	1.23	0.13	—	—	2.32	1.08	—	—	1.50	0.73	—	—	2.38	0.57	—	—	1.78	0.84	—	—	—	—
Pandya (2023)_S2_	*43*	—	—	—	—	1.25	0.13	—	—	2.34	0.98	—	—	1.56	0.84	—	—	2.40	0.56	—	—	1.75	0.44	—	—	—	—

^a^ Frequency within the category.

^b^ Average mean of mother and father scores.

With the data available, it was possible to calculate the pooled percentage distribution of Western‐European samples, irrespective of the sample type (*k* = 21, *n* = 1660), which was 901 Secure (54%), 759 Insecure (46%), specifically 562 Ds (34%), 163 P (9%) and 54 D (3%). The forced three‐way distribution (*n* = 1606) was 56% Secure, 35% Ds and 9% P (44% Insecure).

There were also calculated two‐way and three‐way distributions in Latin American countries (*k* = 3, *n* = 94) being 48 Secure (51%) and 46 Insecure (49%) of which 34 Ds (36%) and 12 P (13%).

Participants in Western European and Latin American samples did not significantly differ in the odds of being classified as Secure (logOR = −0.12 [−0.67 to 0.43], SE = 0.28, variance = 0.08) or Ds (logOR = 0.09 [−0.49 to 0.67], SE = 0.30, variance = 0.08), but Latin‐American participants had higher odds to be classified as P (83.33%; logOR = 0.20 [−0.68 to 1.09], SE = 0.45, variance = 0.20).

**FIGURE 5:**
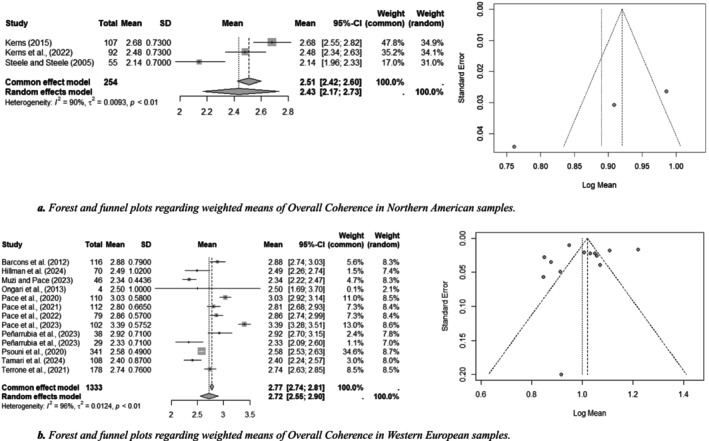
Forest and funnel plots regarding weighted meands of Overall Coherence in Northern America e Western European samples. (a) Forest and funnel plots regarding weighted means of Overall Coherence in Northern American samples. (b) Forest and funnel plots regarding weighted means of Overall Coherence in Western European samples.

Concerning FFI scores, the only comparison possible was Northern American (*k* = 3, *n* = 254) vs. Western European (*k* = 13, *n* = 1333) on the pooled weighted mean of Overall Coherence. Figure 5 shows forest and funnel plots about this scale in the two cultural groups, also showing that there was substantial heterogeneity in ES in both, specifically Northern American (Figure 5a; M_w_ = 2.43, SD_w_ = 0.72; *τ* ^*2*^ = 0.01 [0.01–0.47]) and Western European (Figure 5b; M_w_ = 2.72, SD_w_ = 0.66; *τ* ^*2*^ = 0.01 [0.01–0.04]). SMD revealed a small effect, in terms of lower means in the Northern American samples (Hedge's *d* = −0.43 [−0.57 to −0.30], SE = 0.07, variance = 0.01).

### 3.6 RQ4: Trends, Gaps and Relationships With Other Constructs in FFI Literature

#### 3.6.1 Systematic Review Results

Only with descriptive purposes, Table 5 reports narrative synthesis of results concerning psychometric characteristics and relationships of FFI with other investigated outcomes. Notably, inter‐rater agreement was the most reported psychometric information (67.30% of studies) and other constructs investigated together with attachment were: emotion‐behaviour symptoms (36.5%), verbal IQ (22.92%), caregivers' characteristics such as attachment or reflective functioning (18.75%) and various variables related to child adjustment, e.g., coping or academic skills or alexithymia (16.67%).

**TABLE 5:** Information about FFI psychometric properties and/or relationships with other outcomes provided by studies included in the systematic review^a^ (*N* = 50).

Study	Narrative results (RQ5)	
	Psychometric properties	Relationships with other outcomes^b^, ^c^
Community samples		
Breinholst et al. (2018)	Danish back‐translated authorized version [DAN]. Two raters ICC = 0.86.	Comparatively, there were no differences in attachment patterns between community and clinically anxious children, who however showed lower scores in reflective functioning scales.
Carone et al. (2023)	Italian back‐translated authorized version [IT]. Two raters ICCs from 0.81 to 0.84.	Comparatively, there was no difference in child security according to parental sexual orientation (gay, lesbian or heterosexual). Parental overall coherence in the AAI predicted parental reflective functioning, related to higher child security.
Decarli et al. (2022)	[IT] Two‐raters Cohen's k for agreement on classifications 0.87–0.89. Internal reliability for the ‘overall coherence’ scale is indicated by Cronbach's *α* = 0.91.	Higher levels of anxiety symptoms were predicted by lower scores of quality of contact with the best friend.
Escobar et al. (2014)	[SP] Two‐raters Cohen's k for agreement on classifications 0.94	Comparatively, community adolescents scored higher than adopted ones on YSRb thought problems scale. Thought problems were predicted by an interactive effect between insecure attachment and being non‐adopted.
Kerns et al. (2015)	English original version [EN]. Reliability in terms of intraclass correlations values ranging 0.79–0.82. Convergent validity with the KSSb concerning SB/SH availability of mother and father. FFI and KSS showed overlapping associations with other constructs.	Children peer and academic competence and coping skills were positively related SB/SH availability of both parents and coherence.
Kerns et al. (2022)	[EN] Two raters median ICCs 0.82,	In terms of identity characteristics, securely‐attached adolescents showed more interpersonal and temperamental strengths (e.g., humour, forgiveness, honesty, persistence) and spirituality. Attachment security did not predict intellectual strengths.
Gastelle (2018)^a^	Median alphas 0.90.	Higher verbal IQ and supervision partnership in parenting practices predicted high perceived SB/SH mother and father.
Koehn and Kerns (2018)^a^	Convergent validity between SB/SH scores in the FFI and measuresb of parental sensitivity (CRPBI) and autonomy support (POPS).	Once controlled IQ, age and temperament, attachment security predicted positive emotion, with SB/SH father as unique significant predictor.
Obeldobel and Kerns (2020)^a^	No association between SB/SH scores in the FFI and results of ad hoc observational measures of parent–child interaction in terms of sensitivity and autonomy support (Gastelle 2018).	Attachment security was correlated with hope, love and gratitude.
McConnachie et al. (2020)	[EN], Two raters ICCs from 0.71 to 0.75.	Comparatively, there were differences based on parental sexual orientation, as participants reared in gay families showed higher security and less disorganization or preoccupation than peers reared by heterosexuals and lower preoccupation than lesbian‐reared children. Emotional symptoms at 4–8 years predicted higher attachment disorganization at 10–14 years.
Pace et al. (2020) Muzi and Pace (2022, 2023)^a^ Muzi et al. (2022)^a^	[IT] Two raters inter‐rater Cohen's k ranged 0.84–1, correlations among raters scores 0.29–0.74 Independence to verbal skills Discriminant validity to emotional behavioural problems. Concurrent validity with AAI meta‐analytic international and national adolescent categories distributions. Convergent validity with the IPPA only in community adolescents.	Verbal IQ did not show relations with attachment categories and scales (except school competence). Attachment classifications were unrelated to internalizing and externalizing symptoms. Internalizing symptoms were predicted by an interactive effect of attachment preoccupation and alexithymia, regardless of the type of sample (community, adopted, in residential care); Externalizing symptoms were predicted by the cumulative effect of lower attachment security, high disorganization and alexithymia, regardless of the type of sample (community, adopted, in residential‐care);
Pace et al. (2021) Pace et al. (2020)	[IT] Two raters inter‐rater Cohen's *k* = 1 on categories.	Binge eating symptoms in girls were predicted by attachment dismissal and preoccupation, showing a positive correlation with disorganization.
Pace et al. (2023) Muzi et al. (2021, 2022)^a^	[IT] Two raters inter‐rater Cohen's *k* = 1 on categories. Cronbach's alphas 0.86 (T1) and 0.87 (T2) Test–retest reliability. 93.5% stability of four‐way attachment classification over 4 months. Significant correlations between patterns and coherence scales in T1 and T2.	Internalizing and anxiety symptoms showed positive correlations with preoccupation. Internalizing, social and identity‐related symptoms and somatic complaints showed positive correlations with disorganization. Thought problems in the YSR at T2 (pandemic‐related mild social restrictions) showed positive correlations with disorganization in T1 and preoccupation in T2. The quality of contact with the best friend was a mediator of the effect of SB/SH parents on adolescents' depressive withdrawal.
Peñarrubia et al. (2023)	[SP] Cohen's k values for two raters' inter‐rater agreement from 0.57 to over 0.70	Concerning family structure, adoptees showed higher security in single‐parent adoptive families. Higher verbal IQ was related to higher SB/SH mother.
Psouni and Apetroaia (2014)	Swedish authorized translated version [SE]. Cohen's k for Two raters' inter‐rater agreement on classification 0.82. Reliability for scores between 0.73 and 0.85. Convergent validity with SBS classifications (87%) and between scores about SB/SH mother and father. Convergent validity with KSS scores about security, overall coherence, SB/SH mother and father and adaptive response, but no on other pattern and defensive strategies scales.	NI
Psouni et al. (2020)	[SE], NI. Exploratory Factor Analysis. A 3‐component solution (F1, security; F2: Preoccupation; F3: Idealization) was extracted and discriminated accurately attachment classifications.	NI
Schmidt et al. (2022)	Portuguese [PT] translation validated in Brazil. Internal consistency Cronbach's alpha 0.82.	No differences in internalizing, externalizing or total symptoms between participants classified as clinical or non‐clinical according to CBCLc cut‐off scores. In the non‐clinical group, Ds participants showed higher anxiety, depression than preoccupied and disorganized ones and securely‐attached participants showed lower withdrawal than insecure ones. Mother and father SB/SH showed positive correlations with school and social competence and relationship problems.
Slutsky et al. (2016)	[EN], NI.	Security scores predicted higher curiosity, while dismissing scores predicted lower scores. Disorganization was related to higher avoidance and negative feelings toward donor conception.
Steele and Steele (2005)	[EN], original first development. Cronbach's alpha ranging 0.74–0.88 Longitudinal convergence with another attachment measure. FFI scales related to coherence and SB/SH showed positive correlations with infant‐father attachment in the SSP at 18 months.	FFI scales related to coherence and SB/SH showed positive correlations with parent attachment, i.e., mother/father AAIc outcomes. Verbal IQ was positively correlated to overall coherence in the FFI.
Stievenart et al. (2012)^a^	NI on versions used. Cronbach's alpha 0.83 for four coherence scales in both countries. Configural Invariance Analysis (CFA) on the four scales contributing overall coherence score. Cross‐country invariance in coherence measurement between Belgium and Romania.	NI
Tamari et al. (2024)	Hebrew translated version [ISR], two raters inter‐rater agreement values 0.76–0.86	Verbal IQ was positively related to narrative coherence, but not to SB/SH mother and father. Maternal sensitive guidance in emotional dialogues during preschool (T1) was longitudinally positively associated to adolescents' narrative coherence and SB/SH mother during adolescence, while actual maternal sensitive guidance (T2) was not and this variable was not related to SB/SH father at any time. FFI coherence and parental SB/SH did not show correlations with negative life events in adolescents' life or pubertal development.
Terrone et al. (2021)	[IT], NI.	Gambling disorder symptoms were predicted by insecure attachment, with reflective functioning subscales (developmental perspective and theory of mind friend) as mediators of the effect. Gambling behaviours and symptoms were negatively related to security, reflective functioning and adaptive response subscales and positively with Ds scores and father derogation.
At risk samples		
Abrines et al. (2012)	Spanish translated version [SP]. Significant correlations between scores assigned by two certified raters.	Participants classified as secure showed lower levels of hyperactivity and attention symptoms than insecure ones.
Barcons et al. (2012, 2014)	[SP], NI.	The secure classification of adopted children predicted a more positive relationship with parents and more adaptive skills. No effect of attachment secure/insecure classification on social stress scores.
Bastianoni et al. (2020, 2021) (qualitative studies)	[IT], NI.	Thematic content analysis revealed that adopted adolescents talked more than non‐adopted ones about mental states. Predominant themes in adopted adolescent narratives: Positive relationships with the adoptive family; relational conflict; recognition of significant others' supportive roles; possibility of reparation; fear of rejection or abandonment; and a tendency not to recognize the adoptive family. Themes indicating positive attitudes toward the adoptive family, regarded as a reparative relational space, prevailed.
Esbjørn et al. (2015)	[DAN] scales average two raters ICC = 0.88 (range 0.72–0.91)	Lower scores in overall coherence and quality of contact with the best friend predicted higher levels of anxiety symptoms.
Escobar et al. (2013, 2014)	[SP] Two‐raters Cohen's k for agreement on classifications 0.94.	Adopted participants with secure attachment showed more positive and adaptational outcomes than insecure ones in different tasks of emotional processing and attention. No difference in attachment classification according to maternal educational level. See above for comparison with community peers.
Frazier and Knapp (2020)	NI	No baseline difference in attachment in adolescents in foster care attending psychotherapy or equine therapy.
Groza and Muntean (2016)	NI on the translation used. Cronbach's alphas were 0.96 (reflective functioning), 0.94 (Diversity of feelings) and 0.75 (SB/SH).	Adopted children showing secure attachment were matched with parents showing higher reflective functioning and coherence at the Parent Development Interview [PDI] and more optimal parenting. Insecurely attachment children had adoptive parents showing less optimal parenting.
Hillman et al. (2024) Hillman et al. (2023)^a^	NI	Early adopted adolescents demonstrated greater attachment security and narrative coherence compared to those late adopted. Longitudinally, a lower IQ in childhood predicted higher attachment security during adolescence and childhood attachment insecurity predicted disorganization in adolescence. Those adoptees who were placed without siblings exhibited higher levels of total problems on the YSR and used more maladaptive emotion regulation strategies than those placed with adoptive siblings.
Muntean and Ungureanu (2012)	NI	Secure attachment prevailed in adoptive children matched with adoptive parents showing a negotiating parenting style in the PDI. Insecure attachment prevailed in children matched with parents showing an ineffectual parenting style and no securely attached children had parents showing a punitive parenting style.
Muzi et al. (2024)	[IT] Two raters' inter‐rater agreement on categories *k* = 0.86.	6‐month decrease in total, internalizing and externalizing symptoms levels did not correlate with attachment patterns scores at T1. Disorganization showed a positive correlation with a 6‐month improvement in the youth‐educator relationship as referred by the adolescent in the PARAc. Both dismissal and preoccupation showed correlations with educator‐referred improvements in the youth‐educator relationship, but only higher scores in the Ds pattern predicted higher relational improvements over 6 months.
Muzi and Pace (2023) Muzi and Pace (2020), Pace et al. (2022)^a^ Muzi et al. (2022)^a^	[IT] Two raters' inter‐rater agreement on categories with Cohen's k ranging from 0.89 to 1. Cronbach's alpha 0.88 Convergent validity. FFI and IPPAb did not show convergent results in residential care. FFI scales related to idealization, anger and role reversal predicted IPPA scores.	Comparatively, adolescents in residential care had worse outcomes in attachment than those late‐adopted or from the community. Internalizing symptoms levels were predicted by cumulative effect of disorganization and alexithymia dimensions. See above (Pace et al. 2020) for additional results on multi‐method prediction models on symptoms with attachment and alexithymia.
Ongari et al. (2013) (qualitative study)	NI.	Qualitatively, adopted adolescents showed a longitudinal change in attachment, in terms of reduction of disorganization and gained narrative coherence and reflectiveness from childhood (ASCT) to adolescence (FFI).
Pace et al. (2022) Pace et al. (2015, 2016, 2018, 2019, 2023)^a^ Muzi and Pace (2022, 2023)^a^	[IT] Two raters inter‐rater agreement on categories with Cohen's k ranging 0.91–1. Cronbach's alpha 0.83. Longitudinal convergence with other attachment measures. Discontinuity between attachment classifications during childhood at the SRPc and MCASTc and FFI classifications in adolescence.	Comparatively, no difference in attachment with community peers. Longitudinal changes in attachment suggest a positive effect of the adoption. FFI attachment outcomes were related to AAIc outcomes of adoptive mothers. Internalizing symptoms were predicted by higher attachment disorganization regardless of the group (late‐adopted or community). See above (Pace et al. 2020) for additional results on multi‐method prediction models on symptoms with attachment and alexithymia.
Pandya (2023)	NI on the version used. Single rater, Cronbach's alpha for scales ranging 0.79–0.87. An alternative coding system applied (some scores ranged 5–20 points)	Visible intervention outcomes of a psychoeducational intervention on spirituality and parental abilities in terms of better attachment outcomes in the intervention adopted group versus the control group.
Peñarrubia et al. (2023)	[SP], see above.	See above.
Pierrehumbert et al. (2011)	NI on the version used. Convergent validity between FFI information related to social and school competence and the results of School Success Profile questionnaire.	NI
Zadeh et al. (2017)^a^	[EN] Two raters inter‐rater correlations on pattern scales ranging 0.71–0.84	A positive children's perception of donor conception was positively related to attachment security and negatively with disorganization. No relationships with the interest in donor.
***Clinical samples***		
Breinholst et al. (2018)	[DAN], See above.	See above.
Walczak et al. (2017)	[DAN], two raters inter‐raters ICC = 0.85	Intervention outcomes of Cognitive Behavioural Therapy (CBT) for children with anxiety were not related to children's attachment. Children who responded or no to treatment showed no difference in attachment scores.

*Note:* NI = No information on the topic.

^a^ Duplicate samples only included in the systematic review in case of information useful for the narrative synthesis, merged across studies on overlapping samples.

^b^ Outcomes' measures: AAI = Adult Attachment Interview; CBCL = Child Behavior Checklist 6–18 years; CRPBI = Child Rearing Practices Behavior Inventory; IPPA = Inventory for Parent and Peer Attachment; KSS = Kern Security Scale; MCAST = Manchester Child Attachment Story Task; POPS = Perceptions Of Parent Scale; SBS = Secure Base Scripts; SRP = Separation Reunion Procedure; SSP = Strange Situation Procedure; YSR = Youth Self Report.

^c^ SB/SH = Secure base/safe haven availability of the parent.

#### 3.6.2 Meta‐Analysis of Relationships With Other Constructs

With available data, it was possible to meta‐analyse relationships of FFI scales with several emotional and behavioural problems, i.e., Total problems, Internalizing Problems, Externalizing Problems, Other Problems (Attentional, Social and Thought Problems) and Verbal IQ. Here only significant results are detailed, but the Appendix S1 report detailed results, funnel and forest plots of the meta‐analysis for RQ4 for each outcome investigated and results concerning heterogeneity tests and moderators are reported in Table 3.

#### 3.6.3 Relationships With Emotion‐Behaviour Problems

The meta‐analyses revealed three significant associations: two between higher attachment disorganization and higher levels of both internalizing problems (*r* = 0.20 [0.10, 0.29]) and thought problems (*r* = 0.14 [0.04, 0.24]). Moreover, another association was detected between higher levels of internalizing problems and lower SB/SH father (*r* = −0.12 [−0.21, −0.03]). Associations were all small in magnitude but reliable due to the absence of between‐study heterogeneity or risk of publication biases and no effect of moderators (see Table 3). The rest of association were null and not significant, affected in many scales by moderate between study heterogeneity—especially in the case of relationships with dismissing and preoccupied patterns and SB/SH Father. However, except for the relationships with the SB/SH father scale, meta‐regressions mostly showed no moderation effects of participants' age, gender, sample type or study quality.

#### 3.6.4 Relationships With Verbal IQ

The meta‐analyses including moderators analysis (Table 3) revealed several small but significant associations with secure‐autonomous pattern (*r* = 0.25 [0.06, 0.42]), with moderators older age, female gender and at‐risk status; Ds (*r* = −0.26 [−0.36, −0.16]); overall coherence (*r* = 0.27 [0.14, 0.42]), with moderators female gender and at‐risk status; SB/SH mother (*r* = 0.27 [0.14, 0.42]); SB/SH father (trimmed *r* = 0.21 [0.05, 0.36]) with moderators older age, female gender and at‐risk status.

## 4 Discussion

This systematic review and meta‐analysis synthesize current knowledge on the age‐adapted attachment interview Friends and Family Interview (FFI; Steele and Steele 2005) for middle childhood and adolescence. The information reported includes data from almost 3000 participants in a wide age range covering primary school to college, mostly from Europe and North America.

### 4.1 Aggregate Data in Community, At‐Risk and Clinical Samples and Moderators (RQ1, RQ2)

Aggregated distribution of FFI attachment categories—computed in response to RQ1 ‐mostly confirmed expectations based on literature with secure categories prevailing in community samples and insecure ones in at‐risk samples.

Comparing FFI community distributions with CAI category ranges (Secure: 15%–77%, Ds: 20%–30%, P: 0%–7%, D: 1%–18%, based on Gastelle and Kerns 2022) and AAI distributions in adolescents (F: 50%, Ds: 33%, P: 6%, U: 11%; Bakermans‐Kranenburg et al. 2025), similar rates of Secure and Dismissing categories emerge. However, the FFI shows higher Preoccupied and lower Disorganized rates, prompting reflection.

While both CAI and AAI include narrative questions targeting loss and trauma to detect disorganization, the FFI relies more on video‐based coding for drawing firm conclusions about disorganization. Given the elevated Preoccupied rates and the well‐known difficulty in distinguishing preoccupation from disorganization in narrative interviews (Main et al. 2008), this may reflect a limitation of the FFI's psycholinguistic coding, possibly leading to overestimation of Preoccupied classifications when some might more reasonably fit with the disorganized classification.

Nonetheless, aggregated distributions across all three methods suggest convergent outcomes in identifying three‐way attachment classifications.

Interpretation of the aggregated FFI distribution for the at‐risk sample requires caution. This is partly because the data combine participants classified here as both at‐risk and clinical, whose CAI distributions fall within a wide range (9%–84% Secure, 55%–82% Dismissing, 0%–9% Preoccupied and 9%–35% Disorganized), as reported by Privizzini (2017) and Gastelle and Kerns (2022). In addition, the at‐risk group also includes specific populations, such as adopted children, for whom a distinct AAI adolescent distribution has been documented (Bakermans‐Kranenburg et al. 2025).

Taken together, the pooled FFI results align with the CAI literature in showing predominance of insecure categories and falling within the broad ranges previously reported. When tentatively compared with AAI distributions in at‐risk adults, however, FFI data suggest relatively higher rates of security, comparable levels of Ds and elevated rates of Preoccupation, alongside markedly lower rates of disorganization. One possible explanation is that children and adolescents may be less exposed than adults to adverse life events that contribute to attachment disorganization (Bakermans‐Kranenburg et al. 2025).

At the same time, the observed discrepancies across both community (lower exposure) and at‐risk (higher exposure) groups underscore a recurring concern noted here and elsewhere (Pace et al. 2020): current FFI guidelines for coding disorganization may require refinement. Without such adjustments, there is a risk of misclassifying disorganized patterns as preoccupied, a problem already highlighted in earlier work (Main et al. 2008). Weighted means of FFI pattern scales broadly mirrored category distributions in both community and at‐risk samples. Similarly, pooled scores on narrative coherence and SB/SH aligned with expected patterns, suggesting generally appropriate use of both dimensional and categorical coding systems. However, substantial heterogeneity in effect sizes was found across all FFI scales and groups that, though adjusted for bias, may reflect measurement limitations, urging caution in interpreting results despite overall strong study quality. Notably, previous meta‐analyses and reviews using other methods (Bakermans‐Kranenburg et al. 2025; Madigan et al. 2016; Privizzini 2017) also report high variability in attachment outcomes.

Moreover, consistent with broader findings (Bakermans‐Kranenburg et al. 2025; Gastelle and Kerns 2022), age and gender did not moderate heterogeneity in weighted means (e.g., Muzi and Pace 2020, 2022; Kerns et al. 2022; Peñarrubia et al. 2023; Schmidt et al. 2022). While the lack of gender effects aligns with previous evidence (Bakermans‐Kranenburg et al. 2025; Madigan et al. 2016), the absence of age effects was less expected. This may suggest that developmental differences in attachment are better examined across major life stages—such as infancy, adolescence and adulthood—rather than within a single phase (Verhage et al. 2016; Van IJzendoorn and Bakermans‐Kranenburg 2010).

Also, this meta‐analysis assessed study quality as moderator and it was not significant. Therefore, this heterogeneity in FFI results can ultimately reflect casual and reasonably existing differences across samples and, given the results mainly converge with those obtained with similar attachment assessment methods, findings ultimately support the solidity of the FFI as a reliable method to assess attachment categories and means in clinical and at‐risk minors. Lastly, the pooled distribution of categories in clinical samples of anxious children showed that the majority had secure classifications. This suggests that anxiety disorders are similarly distributed among individuals with secure or insecure attachment. As Kerns and Brumariu (2014) suggested, disorganized individuals may be at a greater risk than insecure ones. However, the absence of children classified as D in the results prevented testing this hypothesis. Furthermore, the exclusive presence of samples with anxiety disorders in this review impedes a comment considering CAI and AAI distributions, which merge different psychiatric conditions. In this regard, the paucity of diverse clinical samples indicates a gap of attachment clinical studies highlighted with several narrative methods (Gastelle and Kerns 2022), also impeding the exploration of potential moderators such as gender or parental attachment states of mind (Barcons et al. 2012; Steele and Steele 2005), ultimately calling for further studies. Also, it may be that the anxiety difficulties reported in the FFI study included in the meta‐analyses may be genetic rather than social in origin, but this would require careful study to disentangle these influences.

### 4.2 Attachment Differences According to Participants' Risk Status and Cultural Background (RQ3)

#### 4.2.1 Differences Among Community, at Risk and Clinical Groups

Expected differences between *at‐risk* vs. *community samples* were confirmed but some counter‐intuitive results emerged. Specifically, it was confirmed that at‐risk participants had higher odds and risk of being classified as Insecure than community ones, in line with the literature with other methods (Bakermans‐Kranenburg et al. 2025; Privizzini 2017). Also, the higher rate of Disorganized in at‐risk than community participants is confirmed (van den Dries et al. 2009). However, community and at‐risk participants did not differ in rates of Ds, contrasting with a general statement reported by single studies that at‐risk children and adolescents tend to present more attachment avoidance than community counterparts (e.g., Barcons et al. 2012; Pace et al. 2020). The difference emerged in terms of more preoccupation in at‐risk samples instead, which can be commented on considering the nature of samples included under the ‘at‐risk’ label in this review. Indeed, heterogeneous conditions with different risks (e.g., adopted and in residential care or abused) have been included in this group based on the exposure to relational adverse and potentially traumatic experiences in the primary attachment environments, such as losses or maltreatment by attachment figures. In this regard, this group includes similar participants to adults exposed to childhood adversities in Bakermans‐Kranenburg et al. (2025), whom the authors found at higher risk and proportion of preoccupied representations over dismissing ones. Therefore, the result of this review can be considered in line with results obtained in adults with the AAI, expanding this knowledge to children and adolescent samples.

Results of the *at‐risk* vs. *community* comparison on standardized mean differences partially confirmed results on categories, highlighting a moderate impact of the at‐risk type of sample in terms of lower security and higher disorganization, no effect on Ds category and a small impact in lowering scores of the perceived availability of parents as secure‐base/safe‐haven. However, a sample type effect on preoccupation was not confirmed, and no expected difference in lower narrative coherence emerged. This can be due to the stated limitations of this review, both in terms of psychometric weakness of weighted means due to study heterogeneity or to the heterogeneity of samples included in the at‐risk groups that can show marked within sub‐groups differences in coherence (Muzi and Pace 2023).

Regarding differences between *clinical* vs. *community* participants, the comparison of both distribution and means revealed no difference in security, contrasting the literature results (Colonnesi et al. 2011). The only difference emerges in the quality of insecurity displayed by clinical participants, with a remarkable prevalence of avoidance over preoccupation or disorganization compared to both community and at‐risk counterparts. Given recent evidence supports a major role of genetic over environmental factors in anxiety (Peel et al. 2023), this result can suggest attachment as not as related to children's anxiety. However, considering the previously stated results (Colonnesi et al. 2011), this result can also be explained remarking the limit of this review to include only children with anxiety disorders up to 13 years old.

#### 4.2.2 Differences According to Cultural Background

Defined country division according to the United Nations (2024), data available was too scarce to respond to the research question appropriately. Available data suggest that the overall FFI four‐way distribution in Western‐European samples, compared with the European adult samples' four‐way distribution in the AAI, i.e., 55% F, 20% Ds, 9% P and 15% U/CC (Bakermans‐Kranenburg et al. 2025), is quite similar in Secure‐autonomous and Preoccupied rates and differs in terms of higher Ds and lower U/CC categories with the FFI, which can be expected considering previously commented here and elsewhere (Bakermans‐Kranenburg et al. 2025) differences between adult and adolescent samples in attachment.

Regarding the Latin American distribution, this study distribution was 3‐way and the AAI one in Bakermans‐Kranenburg et al. (2025) was four‐way, then comparison was made with the ranges reported in Mesman et al. (2016), highlighting a similar rate of Secure‐autonomous but a higher rate of Ds explainable considering the higher avoidance of adolescents versus adults. Furthermore, the only possible comparison between distributions in Western European and Latin samples found no difference in Secure‐autonomous rates but more preoccupied categories in Latinos. This brought original data because up to date this difference has been noted between Northern American or European countries versus Asian or African countries (Mesman et al. 2016), while there was no evidence of a difference with Latinos. On the one hand, this can be due to the at‐risk type of Latin samples pooled in this review, calling for caution and further research in interpreting this difference. On the other hand, these data could expand to middle childhood and adolescence the universality of secure attachment theorized as the prevalent pattern worldwide (Mesman et al. 2016). Variations in the quality of insecurity according to life stage, e.g., adult vs. adolescence and cultural differences in parenting practices and society structuration (Van IJzendoorn and Bakermans‐Kranenburg 2010), should be further investigated to adapt the method of assessment and coding lines appropriately.

Notably, the scarcity of data concerning Northern‐American but also Asian and African countries impeded comparisons similar to those performed with the AAI (Bakermans‐Kranenburg et al. 2025). It was only possible to report lower coherence in Northern American versus Western‐European samples which, however, should be interpreted with caution due to scarce numerosity of the former, where participants were also younger on average. Overall, results and lack of data suggest further investigation before commenting on cultural differences in FFI results.

### 4.3 State‐Of‐Art of FFI Literature and Relationships Other Constructs (RQ4)

Systematic review results confirm and extend the findings and research gaps noted by Gazelle and Kerns et al. (2022). Since 2019, psychometric research on the FFI has grown, generally supporting its validity (e.g., factor structure, invariance, test–retest reliability). However, each property is typically supported by only one study, indicating a need for replication across age groups, countries and language versions. A frequent omission is information on the interview's translation or language used, which raises concern given the importance of standardized training and author‐led supervision to ensure reliability. As well, research trends also mirror broader attachment literature, with similar limitations. For example, clinical studies using the FFI remain scarce and, despite claimed interest in parenting variables such as reflective functioning, states of mind or sexual orientation (Gastelle and Kerns 2022; Camoirano 2017; Carone et al. 2023; Koehn and Kerns 2018), few studies have examined these in relation to the FFI or provided data suitable for meta‐analysis. This highlights the need for broader collaboration and more consistent research focus.

Nevertheless, data available for RQ4 meta‐analyses confirm well established research interest for attachment links with psychopathology (Madigan et al. 2016) and verbal IQ (Deneault et al. 2023), expanding information about them. The verbal IQ link underscores the importance of measuring this in future research and controlling for verbale IQ in correlations between the FFI and external variables.

Findings only partially confirmed links between attachment and emotional and behavioural disorders of children and adolescents (Madigan et al. 2016) or adults (Bakermans‐Kranenburg et al. 2025) found with other attachment mesaures. Specifically, only disorganized attachment was associated with internalizing and thought problems, contradicting broader evidence connecting disorganization to both internalizing and externalizing symptoms and dismissing and preoccupied insecure patterns to internalization (Madigan et al. 2016).

In depth, the link to internalizing problems aligns with Madigan et al. (2016) community findings, showing a small but robust effect. Lower father availability as a secure base was also associated with internalizing symptoms, with some heterogeneity but no publication bias—supporting the elsewhere noted importance of differentiating maternal and paternal roles (Gastelle and Kerns 2022; Madigan et al. 2016). Further, thought problems, often tied theoretically to disorganization and dissociation (Liotti 2004; Kobak et al. 2006), showed a similarly small but solid effect—marking the first meta‐analytic confirmation of the link between attachment disorganization and dissociative symptoms or bizarre thoughts in children and adolescents, previously confirmed only in adults or by single studies (Bakermans‐Kranenburg et al. 2025; Borelli et al. 2010; Obsuth et al. 2014).

Moreover, no moderating effects of age, gender, sample type or study quality emerged, supporting FFI's consistency. However, key moderators identified in prior work, e.g., culture, method, informant (Bakermans‐Kranenburg et al. 2025; Madigan et al. 2016) could not be tested due to low variability. The limited number and scope of studies—mostly Western and CBCL‐based—may explain the absence of expected associations with other insecure patterns or coherence (Gastelle and Kerns 2022).

Lastly, pursuing a trend in broader attachment research to explore links between attachment and verbal IQ, this meta‐analysis found that higher verbal IQ was associated with greater attachment security, narrative coherence, both parents' availability as SB/SH and lower dismissing scores. These results contrast with earlier findings suggesting FFI scores as independent of verbal ability (Steele and Steele 2005; Pace et al. 2020), but some specificities of this study results can help interpret this discrepancy. On the one side, moderation analyses revealed stronger associations between verbal IQ and attachment security among younger participants, females and at‐risk samples, echoing trends observed in adult AAI studies (Bakermans‐Kranenburg et al. 2025) and calling for meta‐analyses with larger samples enabling the parsing of age group, gender and sample status. Furthermore, aside from Hillman et al. (2023), all verbal IQ data here meta‐analysed came from translated FFI versions. As Bakermans‐Kranenburg et al. (2025) suggest that AAI coding guidelines may need adaptation across cultures and language itself may be included as a possible moderator, one can hypothesize some language‐based reasons for discrepant results, that remained untested here due to the limited number of studies and country‐level comparisons.

Overall, discrepancies in findings—both within the FFI literature and compared to broader attachment research using other methods—highlight the need to expand the FFI evidence base, enabling more refined and methodologically robust meta‐analyses.

### 4.4 Strengths and Limitations

This single‐method review brought conspicuous and sometimes original information concerning the Friends and Family Interview specifically, but also general contributions to knowledge about attachment in middle childhood and adolescence. Substantial efforts have been made to ensure a rigorous and inspectable review and meta‐analysis process to maximize the quality of the information reported. However, this work has limitations influencing the goodness of results.

For the systematic review, the imbalance toward Western‐European studies limits the generalizability of results remarking an urgent need for country‐diverse and inter‐country research, especially in Africa and Asia. In this regard, despite common practice in performing systematic reviews, limiting the search to contributions in English may have limited the retrieval of evidence to overcome this and future upgrades of this review should involve multilingual research teams for more comprehensive inclusion criteria. Further, the NOS quality evaluation identified ‘selection’ and ‘ascertainment’ as weaker areas, reflecting common limitations in non‐experimental social science studies. While this indicates overall strong research quality, it also suggests a need for improved reporting on sample selection, response rates and exposure details. Moreover, given the word limit, it was necessary to focus on certain information excluding the discussion on other interesting information present in the included studies which should be further investigated, e.g., the impact of attachment on intervention outcomes (Pandya 2023; Walczak et al. 2017), on new relationships after attachment ruptures (Escobar et al. 2014; Muzi et al. 2024) and relationships with professional caregivers' characteristics (Muzi et al. 2024). Also, the choice of extracting data only concerning the psycholinguistic‐coded scores impeded providing information about studies employing the FFI non‐verbal coding guidelines, potentially crucial to cope with the emerged potential weakness of the interview in detecting disorganization, calling for future reviews on the topic. Concerning the meta‐analysis, a noteworthy limitation was the high heterogeneity across studies, which likely arose from the quasi‐experimental, single‐sample design of most included studies. Although such variability can offer insights into real‐world differences, it also complicates the interpretation of pooled effect sizes, even after adjusting for potential publication bias. While heterogeneity presents an opportunity to explore moderating factors and refine future research, it remains a challenge to fully mitigate its impact. Future studies could focus on standardizing data collection and coding methods to reduce heterogeneity and enhance the robustness of findings.

## 5 Implications for Practice and Conclusion

In conclusion, this systematic review and meta‐analysis suggests the Friends and Family Interview (FFI) as a promising option to assess attachment during middle childhood and adolescence. However, despite the interview being available in different languages, it is important to note that the implications stated below can be considered valid for researchers and practitioners in European and eventually American countries, for whom there is more evidence. In contrast, there is not enough evidence to extend these suggestions to colleagues in Asia, Africa or Oceania.

As implications, current findings inform results obtained from the FFI mostly align with those obtained with other validated methods to assess attachment, supporting the value of the interview in collecting information about attachment and significant relational skills or developmental competence of children and adolescents. However, the research is still limited and, given training for administering and coding the FFI is on payment and not free (like each attachment—narrative, observational, projective—measure, e.g., AAI, CAI, SSP), clinicians and scholars need to make individual choices about whether to invest time and money to learn the FFI scoring system.^1^

## Funding

The authors have nothing to report.

## Conflicts of Interest

The authors declare no conflicts of interest.

## Supporting information


**Appendix S1:** Supporting information.

## Data Availability

The data that support the findings of this study are available on request from the corresponding author. The data are not publicly available due to privacy or ethical restrictions.

## Endnotes

^1^
All queries regarding FFI training should be sent to the Howard Steele<steeleh@newschool.edu>.
