# Supplementary material for: Twenty Years of Attachment Research With the Friends and Family Interview: A Systematic Review and Meta‐Analysis
Source: Clin Psychol Psychother. 2026 Jan 16;33(1):e70203. doi: 10.1002/cpp.70203 (PMC12811740; doi:10.1002/cpp.70203)
Supplement: Supplementary file 1 — Appendix S1: Supporting information. [file CPP-33-e70203-s001.docx]

**Appendix A - RQ4 detailed results^[[1]](#footnote-1)^ for the meta-analysis on FFI relationships with:**

**I. Total problems**

**I.1. Security:**

The aggregated correlation with total problems indicated a non-significant negative effect (r = –0.04, 95% CI [–0.13, 0.05]). There was no heterogeneity (τ² = 0, I² = 0%), with a non-significant Q-test, Q(6) = 3.3, p = .77, suggesting no between-study variability. Egger’s test indicated no potential publication bias (z = –0.42, p = .674). The trim-and-fill procedure did not impute any missing studies, and the adjusted effect remained virtually unchanged (r = –0.04, 95% CI [–0.13, 0.05]).
Meta-regression (k = 7) showed no residual heterogeneity (R² = 0%), with a non-significant test for residual heterogeneity (QE(2) = 0.62, p = .73). None of the moderators—sample risk, gender, age, or study quality—significantly predicted the effect size (QM(4) = 4.56, p = .335).


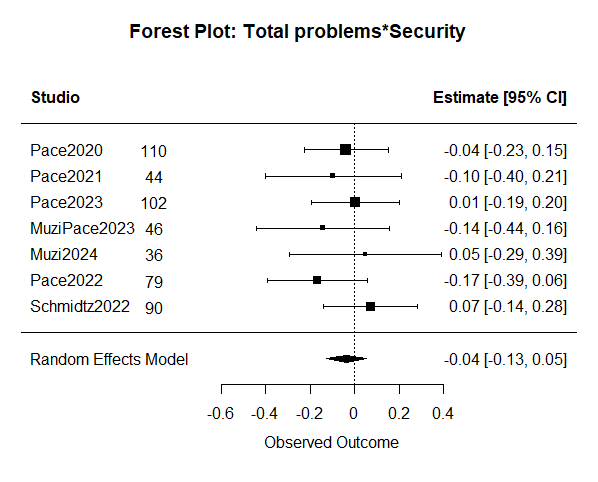

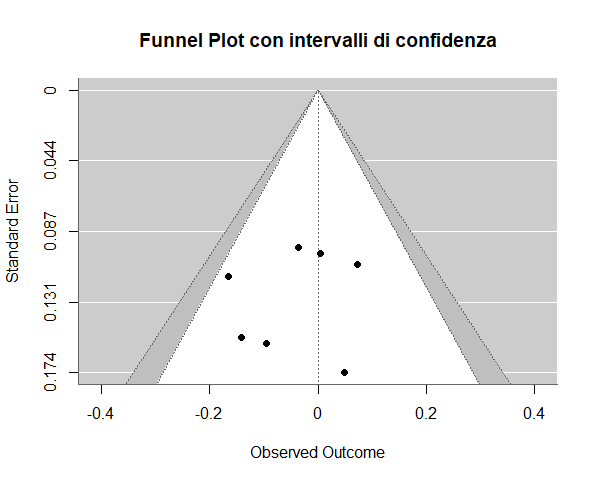


**I.2. Dismissing:**

The aggregated correlation with total symptoms indicated a non-significant negative effect (r = –0.15, 95% CI [–0.41, 0.14]). There was substantial heterogeneity across studies (τ² = 0.13, I² = 90%), with a highly significant Q-test, Q(6) = 74.9, p < .001, indicating notable between-study variability. Egger’s test did not reveal evidence of publication bias (z = 1.11, p = .266). The trim-and-fill procedure imputed two missing studies, adjusting the effect to r = –0.26 (95% CI [–0.49, 0.00]). Meta-regression (k = 7) failed to account for heterogeneity (R² = 0%), and the residual heterogeneity remained significant (QE(2) = 46.78, p < .001). None of the tested moderators—sample risk, gender, age, or study quality—significantly predicted the effect size (QM(4) = 1.29, *p* = .863).


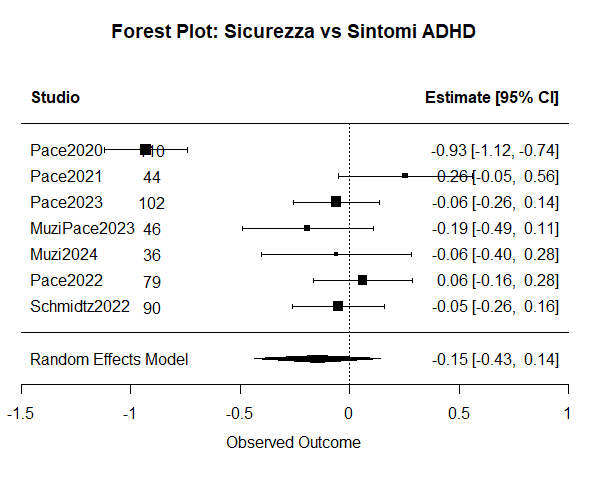

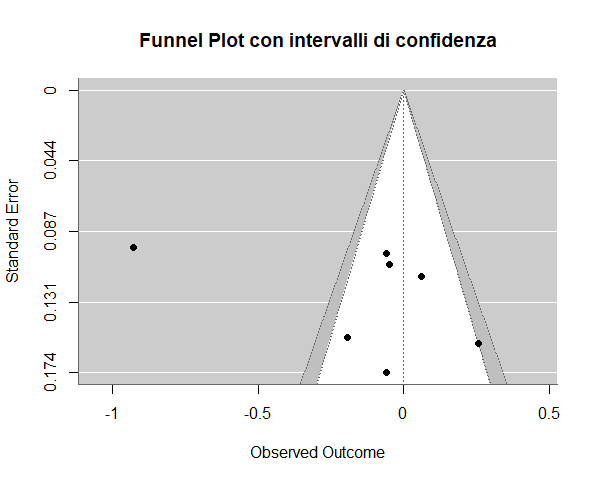


**I.3. Preoccupation:**

The aggregated correlation revealed a small, non-significant positive effect (r = 0.08, 95% CI [–0.01, 0.17]). Heterogeneity was low (τ² = 0.002, I² = 10.6%), with a non-significant Q-test, Q(6) = 6.8, p = .34, indicating limited between-study variability. Egger’s test did not suggest publication bias (z = 0.77, p = .439). The trim-and-fill procedure imputed one potentially missing study, adjusting the effect to r = 0.06 (95% CI [–0.04, 0.16]). Meta-regression (k = 7) not accounted for residual heterogeneity (R² = 0%), but the remaining heterogeneity was not significant (QE(2) = 3.22, p = .199). None of the moderators—sample risk, gender, age, or study quality—significantly predicted the effect size (QM(4) = 2.58, p = .640).


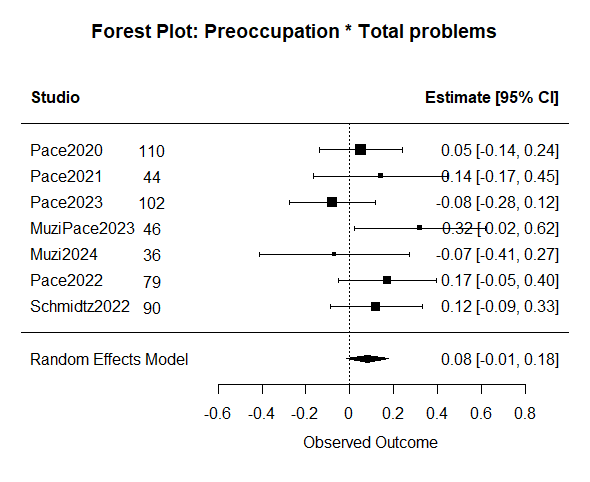

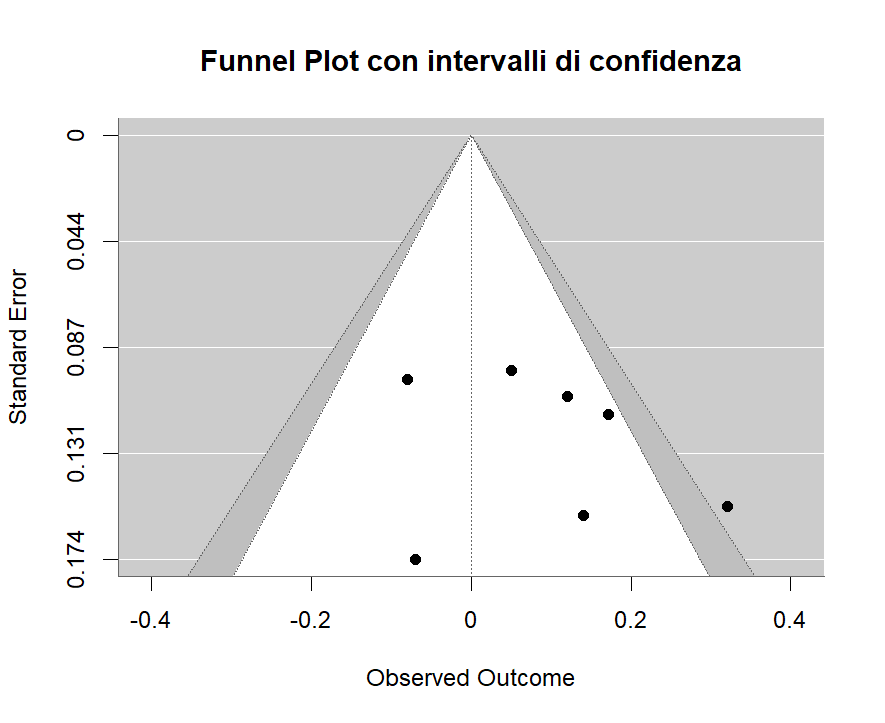


**I.4. Disorganization**:

The aggregated effect size for total problems revealed a small, non-significant positive association (r = 0.09, 95% CI [–0.00, 0.19]). Heterogeneity was virtually absent (τ² = 0, I² = 0%), and the Q-test confirmed minimal between-study variability (Q(6) = 1.3, p = .93). Egger’s test showed no indication of publication bias (z = 0.74, p = .462), and the trim-and-fill procedure imputed two studies, slightly adjusting the estimate to r = 0.07 (95% CI [–0.02, 0.16]).
**Meta-regression** (k = 6) indicated no residual heterogeneity (R² = 0%, QE(1) = 0.02, p = .879), and none of the tested moderators—sample risk, gender, age, or study quality—significantly explained the variance in effect sizes (QM(4) = 1.28, p = .864). All coefficients remained non-significant, with no trend-level effects observed.


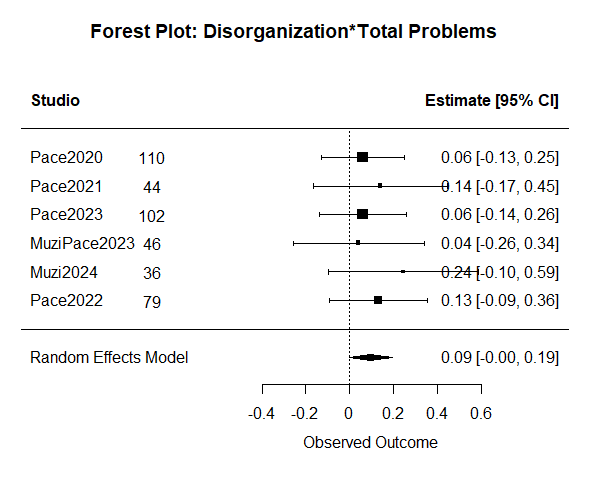

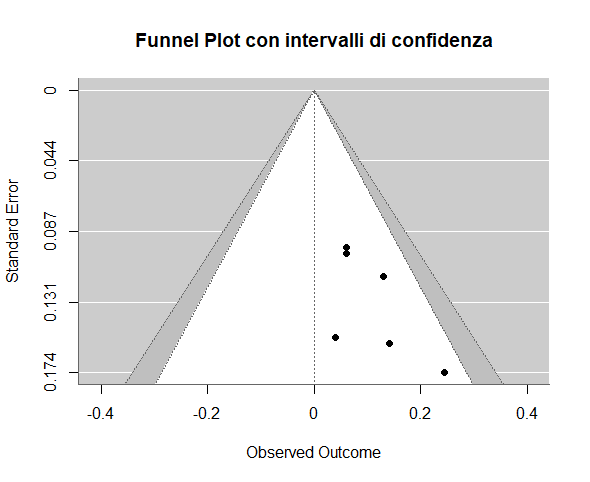


**I.5. Overall Coherence:**

The overall effect size was small and negative but non-significant (r = –0.05, 95% CI [–0.15, 0.04]). Heterogeneity was minimal (τ² = 0.001, I² = 4.5%), with the Q-test showing no significant between-study variability (Q(6) = 6.0, p = .42). Egger’s test did not indicate publication bias (z = –1.24, p = .216), and the trim-and-fill method imputed two studies, adjusting the effect size slightly toward null (r = –0.01, 95% CI [–0.10, 0.09]).
Meta-regression analyses (k = 7) revealed no significant residual heterogeneity (R² = 0%, QE(2) = 2.08, p = .353) and no evidence that sample risk, gender, age, or study quality moderated the effect (QM(4) = 3.93, p = .416). All moderator coefficients were non-significant and close to zero, suggesting a robust null effect across the examined subgroups.


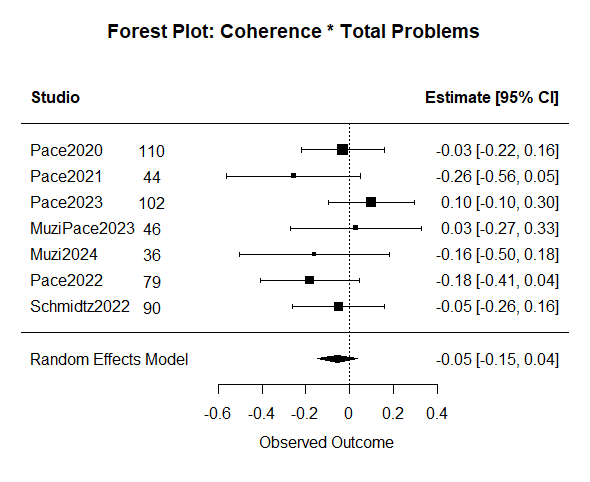
**
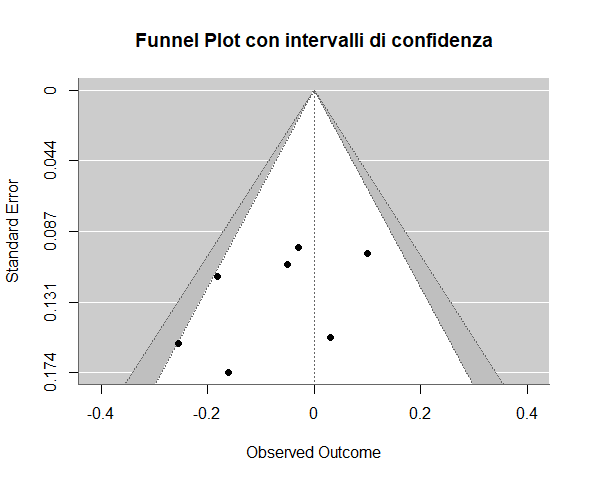
**

**I.6. SB/SH mother:**

The overall effect size was small and negative but non-significant (r = –0.05, 95% CI [–0.14, 0.04]). No heterogeneity was detected (τ² = 0, I² = 0%), and the Q-test indicated no significant between-study variability (Q(6) = 4.5, p = .60). Egger’s test showed no evidence of publication bias (z = –0.78, p = .437), and the trim-and-fill procedure did not impute any missing studies, confirming the robustness of the observed effect. Meta-regression analyses (k = 7) revealed no significant residual heterogeneity (R² = 0%, QE(2) = 1.71, p = .426) and no moderating effects for sample risk, gender, age, or study quality (QM(4) = 2.83, p = .586). All moderators had non-significant coefficients with wide confidence intervals, reinforcing the consistency of the null effect across subgroups.


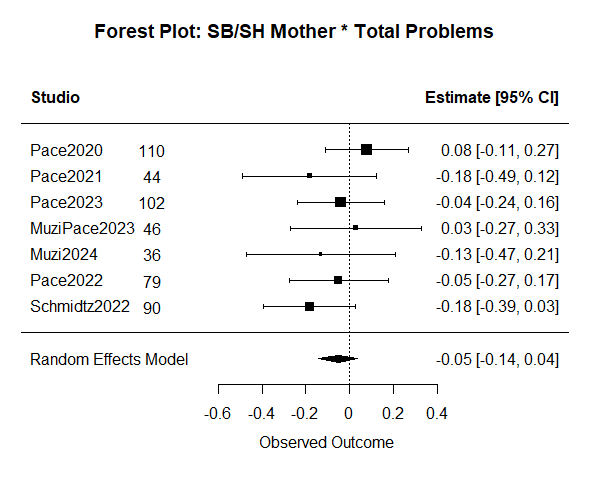

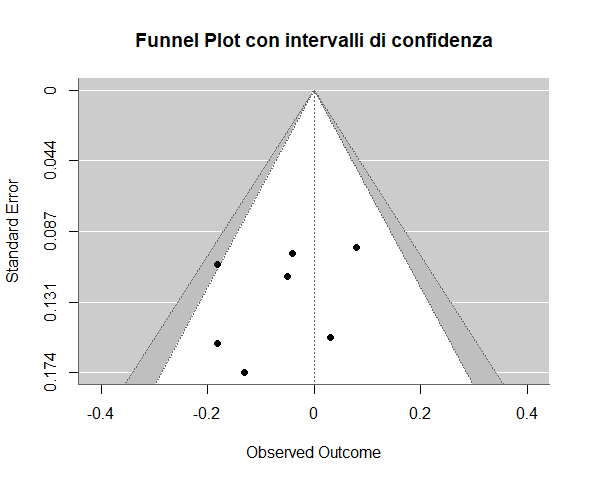


**I.7. SB/SH father**:

The overall effect size was small and negative but not statistically significant (r = –0.10, 95% CI [–0.23, 0.04]). Moderate heterogeneity was detected (τ² = 0.016, I² = 52.5%), and the Q-test indicated significant variability among studies (Q(6) = 12.9, p = .045), suggesting that the true effect size may vary across samples. Egger’s test showed no evidence of publication bias (z = 0.58, p = .561), and the trim-and-fill procedure did not impute any missing studies.
Meta-regression analyses (k = 7) indicated no substantial residual heterogeneity (R² = 100%, QE(2) = 0.95, p = .621), and the tested moderators significantly explained variance in effect sizes (QM(4) = 11.95, p = .018), specifically sample risk (B = -0.35, p = 0.005 [-0.59, -0.10]), gender (B = 0.02, p = 0.002 [0.008, 0.034]) and study quality (B = 0.96, p = 0.004 [0.30, 1.63]).


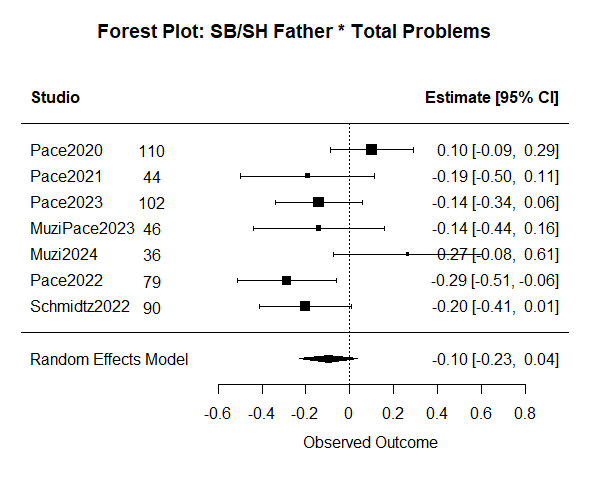

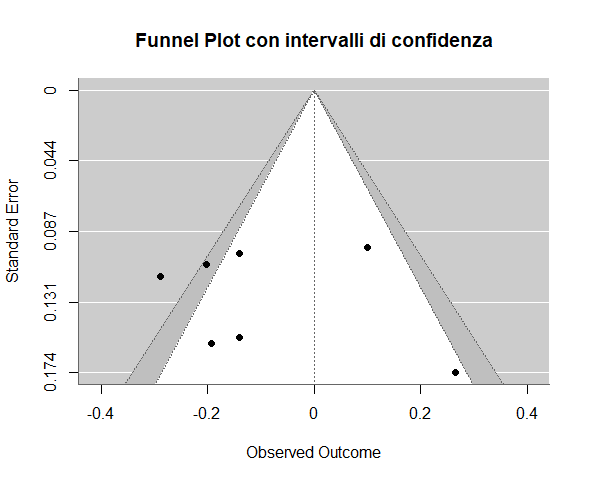


**II. Internalizing Problems**

**II.1. Security:**

The meta-analysis revealed a small, marginally non-significant negative association between the variable and internalizing problems (r = –0.10, 95% CI [–0.21, 0.00]). Heterogeneity was low to moderate (τ² = 0.005, I² = 25.9%), with the Q-test indicating no significant between-study variability (Q(6) = 8.1, p = .23). However, Egger’s test suggested potential publication bias (z = –2.44, p = .0146), and the trim-and-fill method imputed 2 studies, adjusting the effect size to a smaller and fully non-significant value (r = –0.06, 95% CI [–0.16, 0.04]).
Meta-regression results (k = 7) showed that none of the tested moderators—sample risk, gender, age, or study quality—significantly predicted effect size variability (QM(4) = 6.50, p = .165), and the residual heterogeneity was negligible (R² = 100%, QE(2) = 1.62, p = .444). These findings suggest a weak and uncertain association between the variable and internalizing problems, potentially influenced by publication bias and unexplained study-level factors.


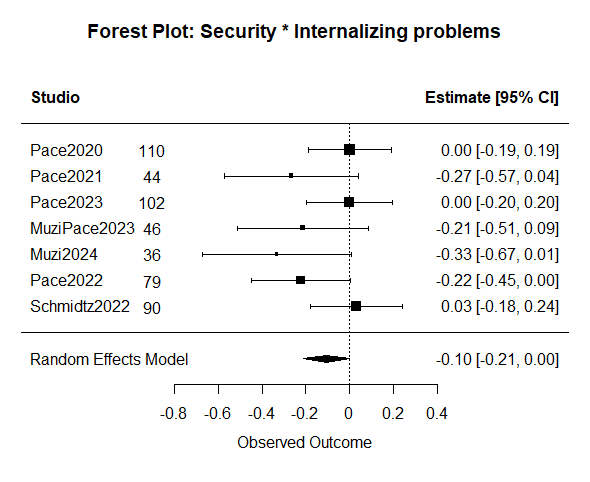

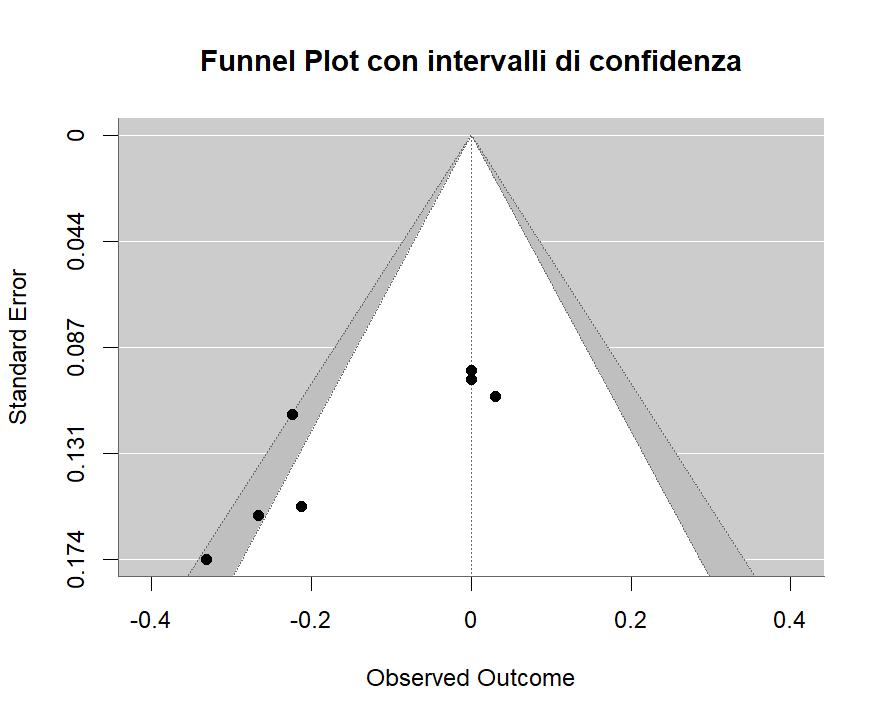


**II.2. Dismissing:**

The meta-analysis revealed a small, non-significant negative association (r = -0.05, 95% CI [-0.170, 0.074]). Moderate heterogeneity was present (I² = 45%, τ² = 0.012), though not statistically significant (Q(6) = 11.3, p = 0.08). No evidence of publication bias emerged (Egger's test: p = 0.743), and the Trim & Fill method did not impute additional studies.
***Meta-regression*** with moderators sample, age, gender and methodological quality showed no significant effects (QM(4) = 3.62, p = 0.460). However, residual heterogeneity remains high (τ² = 0.020; I² = 63.1%), suggesting the presence of other sources of variability not considered in the model.


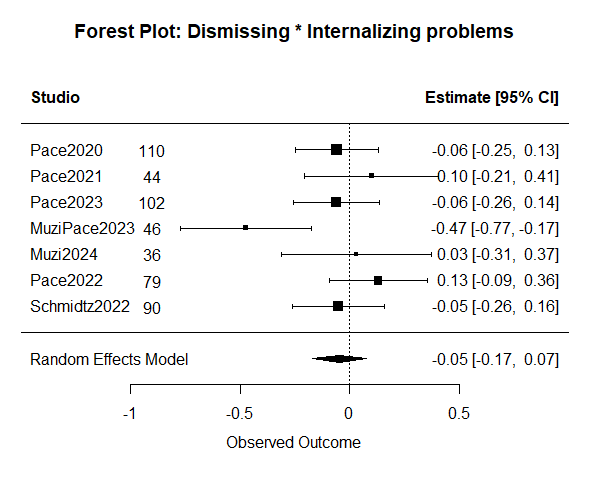

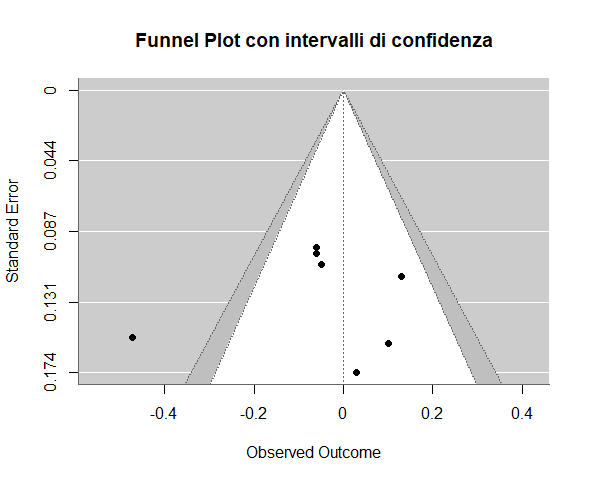


**II.3. Preoccupation:**

A The pooled effect size was small and positive, but not statistically significant, r = .13, 95% CI [−.02, .28]. The test for heterogeneity was significant, Q(6) = 16.1, p = .013, indicating substantial heterogeneity across studies (τ² = .029, I² = 66.4%). There was no indication of publication bias based on Egger’s regression test, z = 0.74, p = .462. The Trim and Fill procedure did not impute additional studies, suggesting that the observed effect was not meaningfully distorted by missing data.
A mixed-effects ***meta-regression*** not explained residual heterogeneity (R² = 0%), revealing substantial heterogeneity unexplained (I² = 73.91), QE(2) = 7.75, p = .021. The overall model was not significant, QM(4) = 4.03, p = .401, indicating not effect of moderator tested.


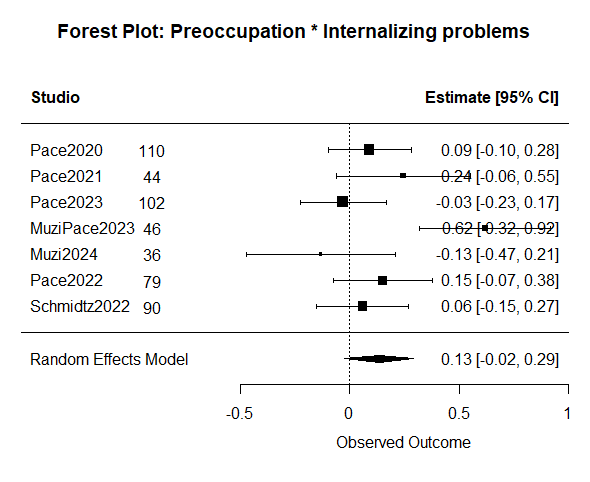

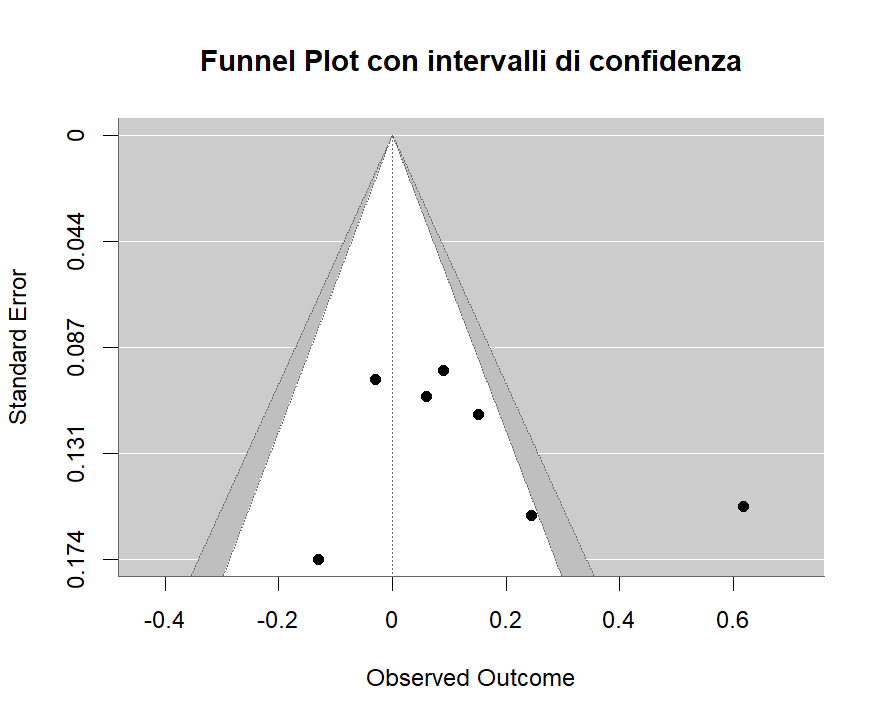


**II.4. Disorganized:**

The pooled effect size was statistically significant and small-to-moderate in magnitude, *r* = .20, 95% CI [.10, .29], suggesting a positive association. There was no evidence of heterogeneity across studies, with Q(6) = 3.60, p = .610, and both τ² and I² equal to 0, indicating consistency in effect sizes. Egger’s test did not detect funnel plot asymmetry, z = 0.66, p = .512, and the Trim and Fill method did not impute any missing studies, suggesting a low risk of publication bias. A meta-regression including sample type, gender, age, and study quality as moderators did not explain significant variance in effect sizes, QM(4) = 3.27, p = .514. None of the individual moderators reached significance (ps > .14), and residual heterogeneity remained negligible (QE(1) = 0.29, p = .592; τ² = 0, I² = 0%).


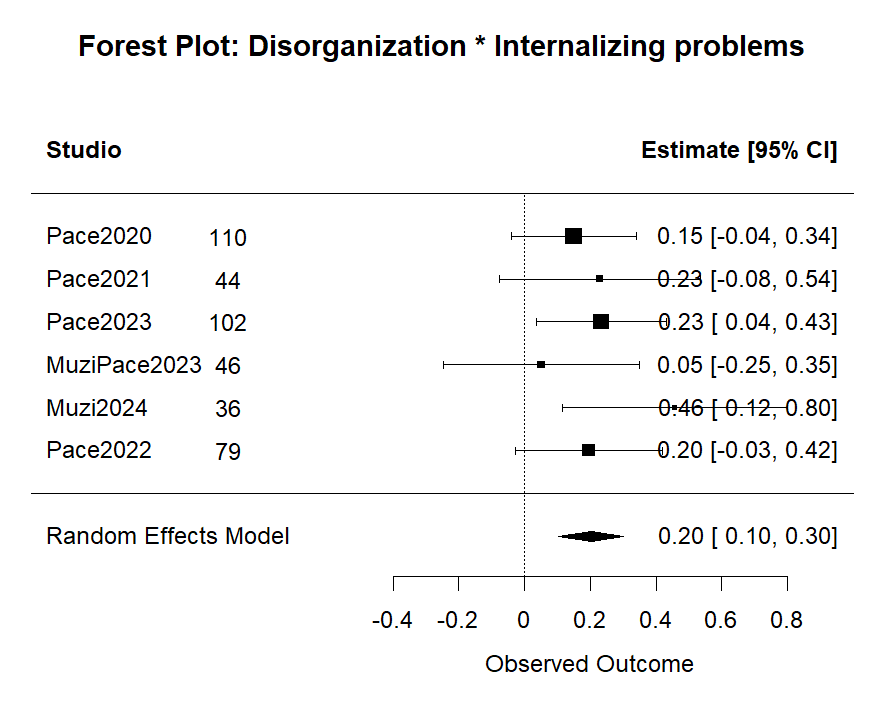

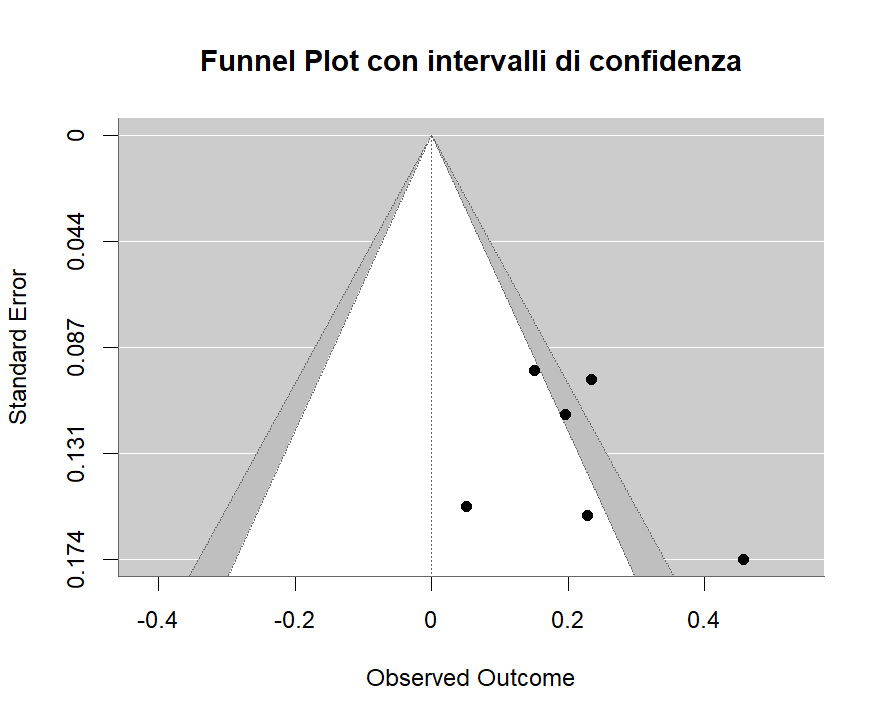


**5. Overall Coherence:**

The pooled effect size was negative but not statistically significant, r = –.11, 95% CI [–.22, .00], indicating a small and inconclusive association. Moderate heterogeneity was observed, with I² = 36% and τ² = 0.008. However, the Q-test for heterogeneity was non-significant, Q(6) = 9.5, p = .15, suggesting that variability across studies may be due to chance. Egger’s test indicated significant funnel plot asymmetry, z = –2.12, p = .034, raising the possibility of publication bias. Nevertheless, the Trim and Fill method imputed only one study, and the adjusted effect remained non-significant, r = –.08, 95% CI [–.20, .04].
***Meta-regression*** analyses with sample risk, gender, age, and study quality as moderators revealed no significant effects, QM(4) = 3.92, p = .417. None of the individual moderators contributed significantly to the prediction of effect sizes, and residual heterogeneity remained moderate (I² = 44.35%).


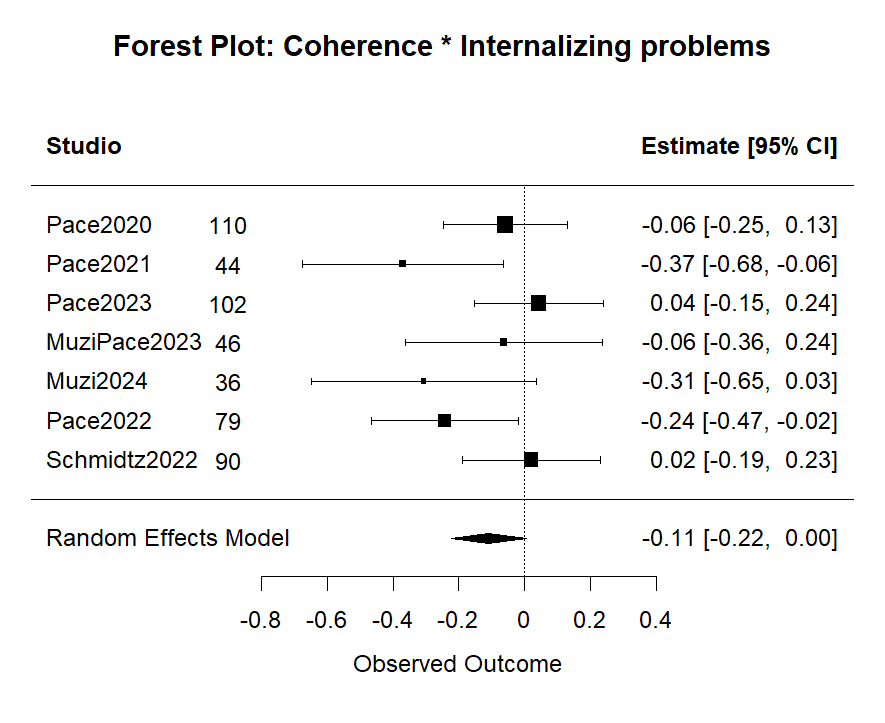

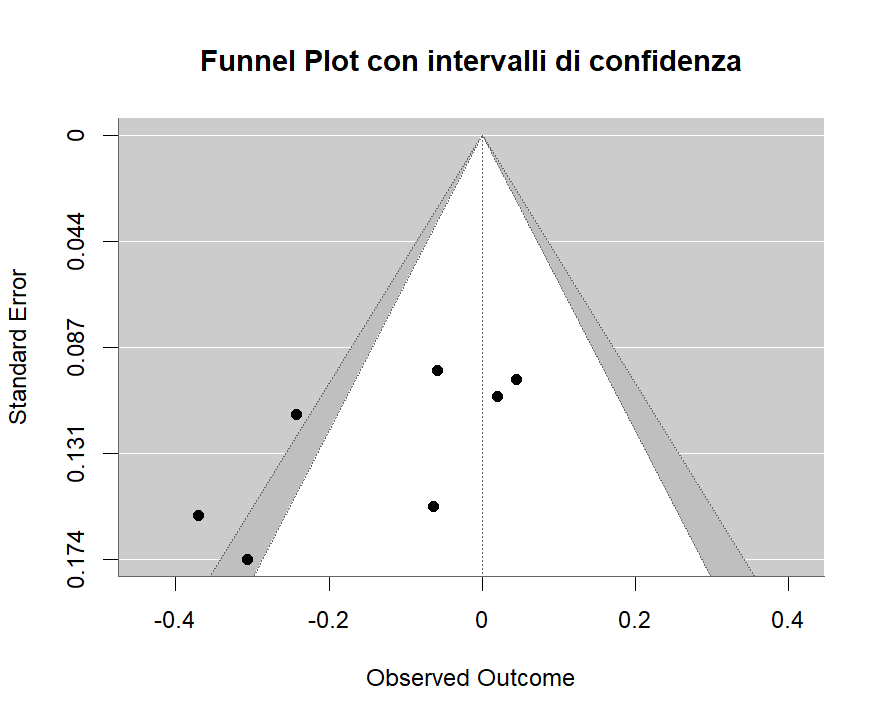


**6. SB/SH Mother:**

The pooled effect size was small and non-significant, r = –.03, 95% CI [–.12, .06], indicating no reliable association across studies.
There was no evidence of heterogeneity among the included studies, with τ² = 0, I² = 0%, and a non-significant Q-test, Q(6) = 3.3, p = .77, suggesting that the observed variation is likely due to sampling error. Egger’s test for publication bias was not significant, z = –1.38, p = .17. The Trim and Fill procedure imputed two studies, but the adjusted effect remained small and non-significant, r = –.01, 95% CI [–.09, .07].
A ***meta-regression*** including sample risk, gender, age, and study quality as moderators showed no significant effects, QM(4) = 2.21, p = .698. None of the individual moderators significantly predicted effect sizes, and residual heterogeneity remained absent (*I*² = 0%).


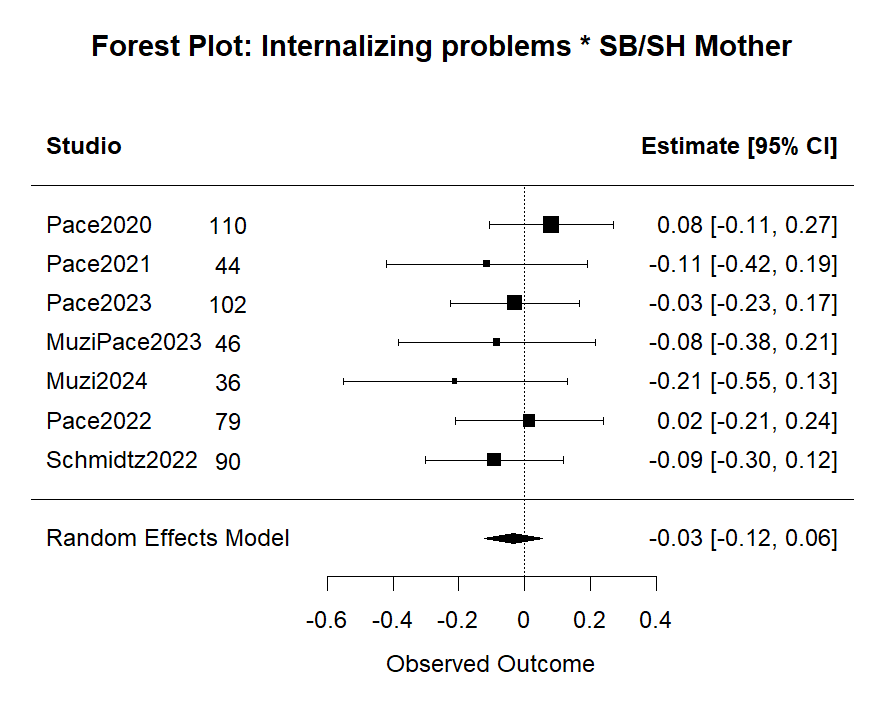

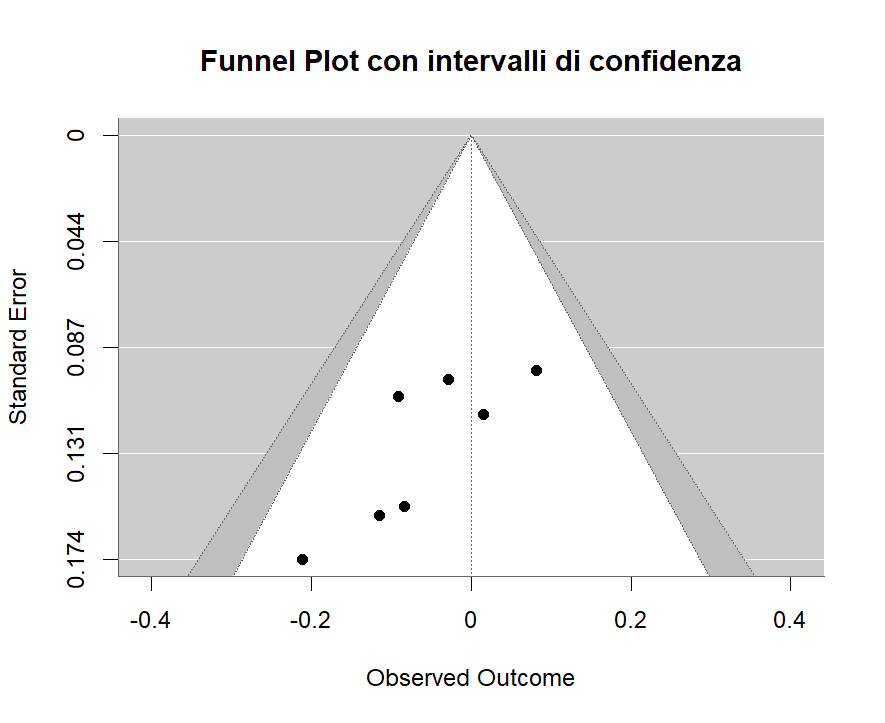


**7. SB/SH Father:**

A random-effects meta-analysis revealed a small but statistically significant negative effect, r = –.12, 95% CI [–.21, –.03], indicating a modest inverse association. There was no evidence of heterogeneity among the included studies, with τ² = 0, I² = 0%, and a non-significant Q-test, Q(6) = 2.9, p = .82, suggesting consistency across study results. Egger’s test indicated no significant funnel plot asymmetry, z = –0.002, p = .998, and the Trim and Fill method did not impute any missing studies, supporting the absence of publication bias.

***Meta-regression*** analyses examining sample risk, gender, age, and study quality as moderators found no significant effects, QM(4) = 1.35, p = .85.

**
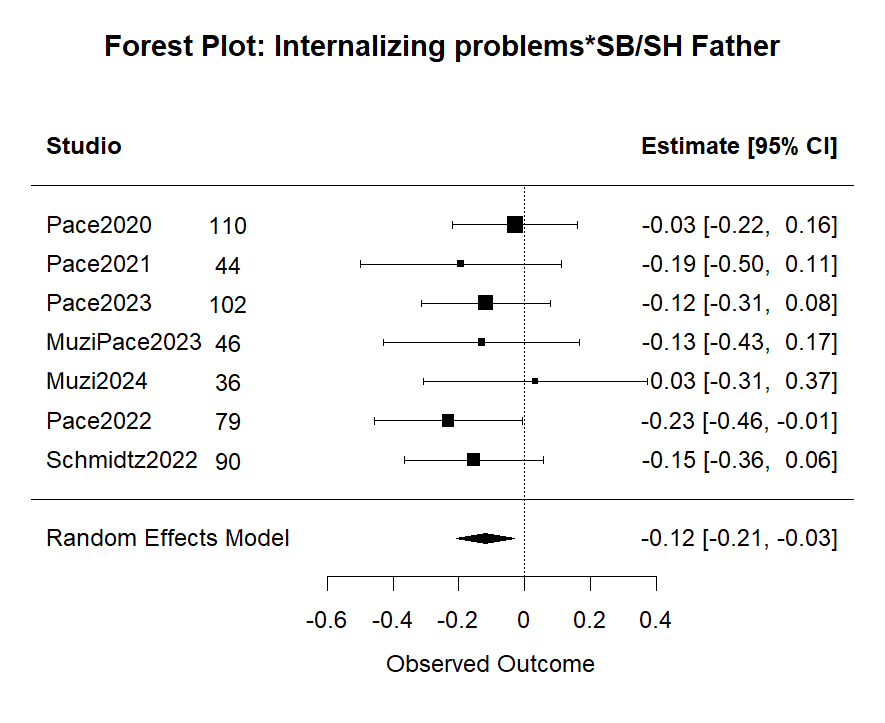
**
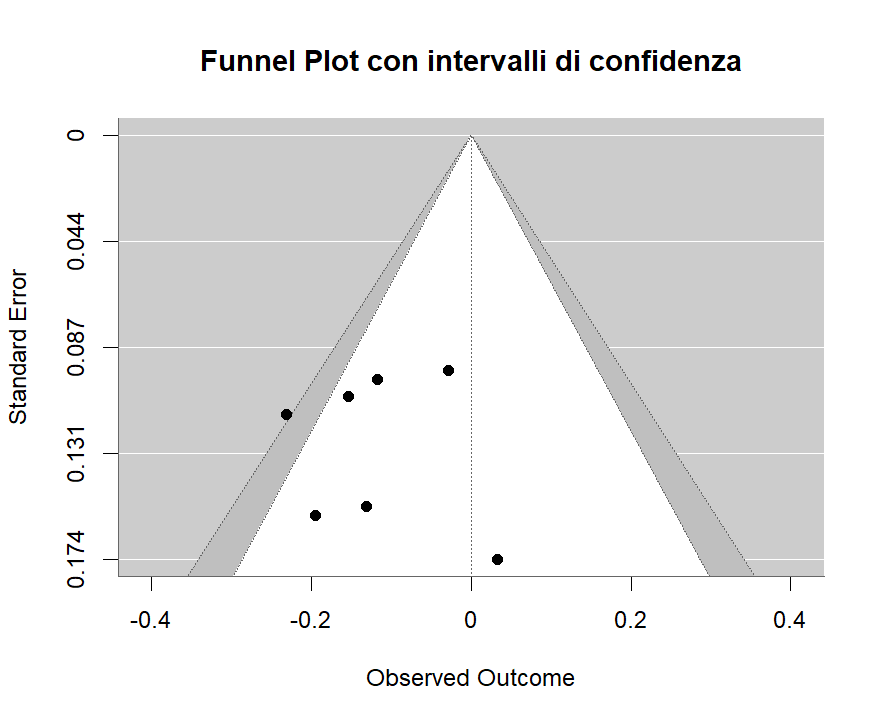


**III. Externalizing problems**

**III.1. Security:**

The aggregated correlation with security indicated non-significant negative effect (*r* = –.02, 95% CI [–.11, 0.07]). There was no heterogeneity (*τ²* = 0, *I²* = 0%), with a non-significant Q-test, Q(6) = 6.4, *p* = .40, suggesting no between-study variability. Egger’s test indicated no potential publication bias (*z* = –1.23, *p* = .216), and the results remained the almost same with the trim-and-fill procedure (*r* = –.019 (95% CI [–.11, .007]).
***Meta-regression*** (*k* = 7) explained all residual heterogeneity (*R*² = 100%), with no significant residual heterogeneity left (*QE*(2) = 0.70, *p* = .70). Still, none of the moderators—sample risk, gender, age, or quality—were significant predictors of the effect size (*QM*(4) = 5.65, *p* = .023).


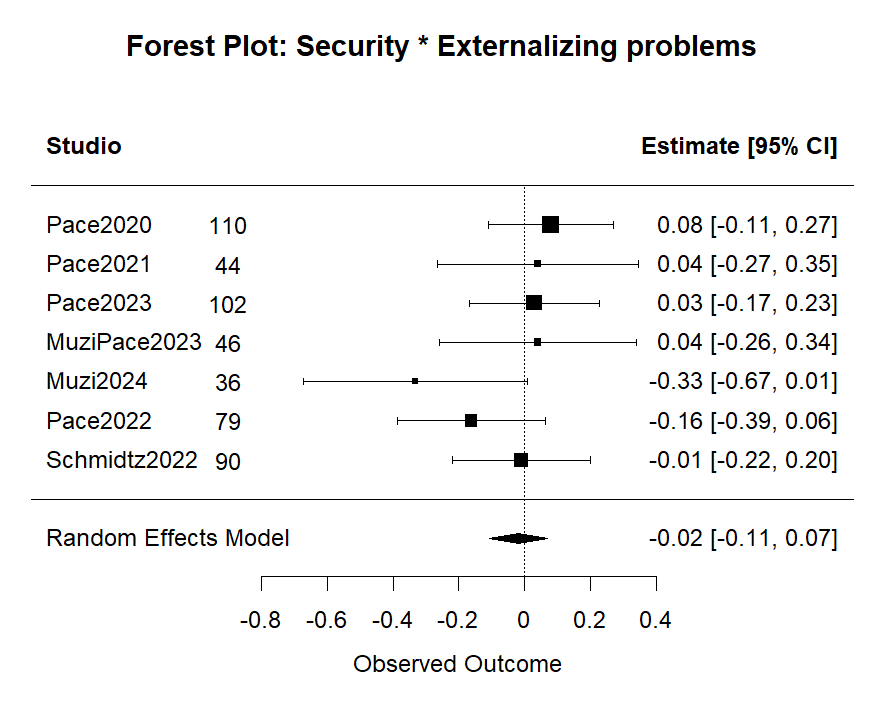
**
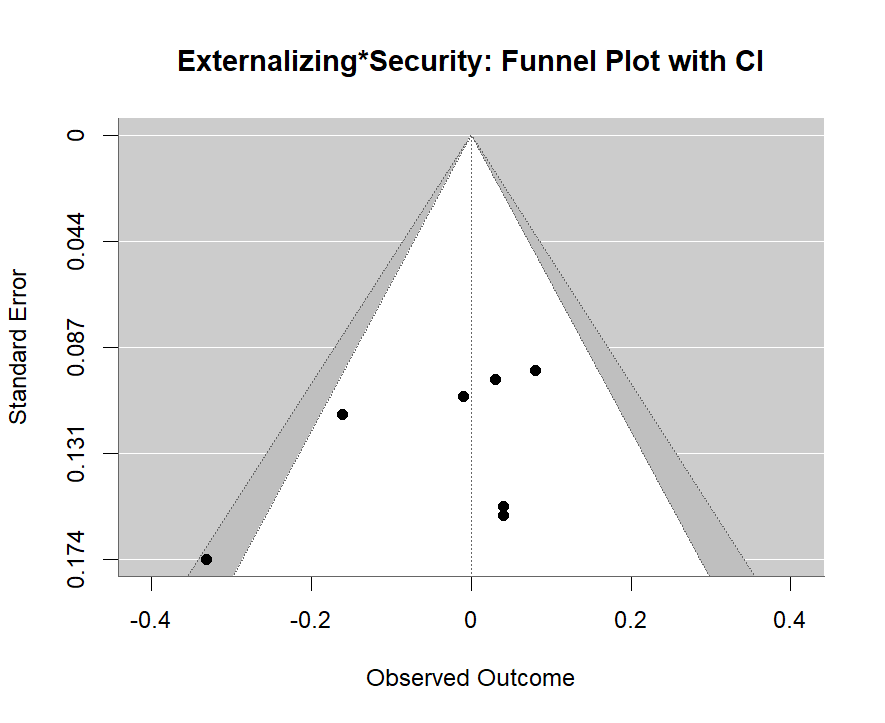
**

**III.2. Dismissing:**

The meta-analysis revealed a small, non-significant negative effect size (*r* = –.09, 95% CI [–.45, .029]), indicating minimal evidence of an association. Heterogeneity was substantial (*τ²* = 0.261, *I²* = 94.7%), with the Q-test approaching significance, *Q(6)* = 143.7, *p* < .001, suggesting high variability across studies. Egger’s test showed no indication of publication bias (*z* = 1.65, *p* = .099). The trim-and-fill procedure imputed two missing studies, adjusting the effect size to *r* = -0.22 (95% CI [-0.52, 0.12]), suggesting a negligible effect size.
***Meta-regression*** *(k* = 7) indicated substantial residual heterogeneity (I² = 93%) not accounted for by the included moderators. The amount of heterogeneity explained was R² = 33%, and residual heterogeneity was significant (*QE*(2) = 32.40, *p* < .001). Test of moderators was not significant, *QM*(4)=6.62, p= .157, but risk status was a significant moderator, B =1.11 , p = .022 (0.16, 2.06).
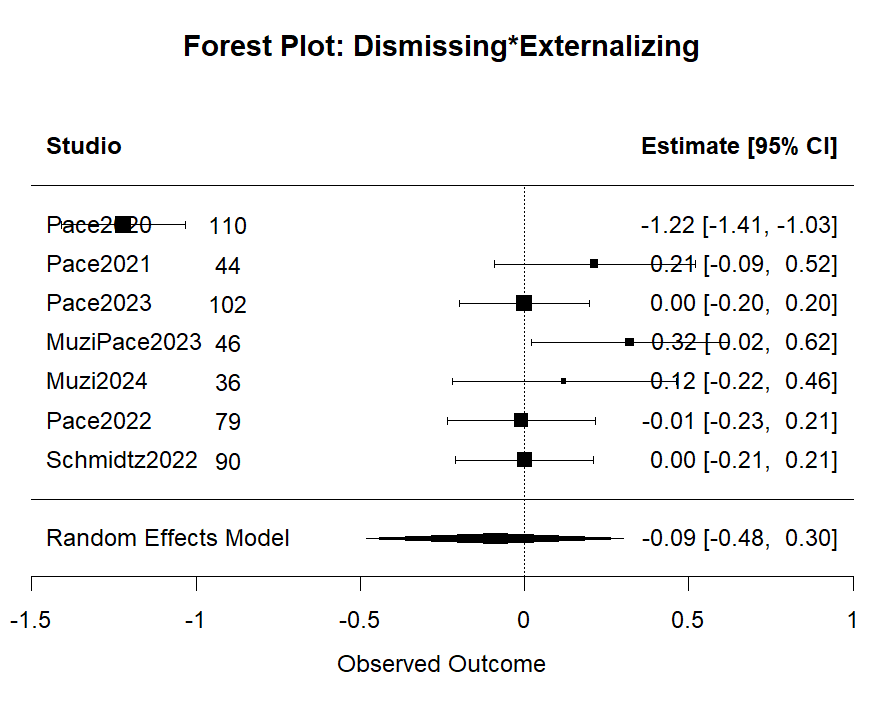

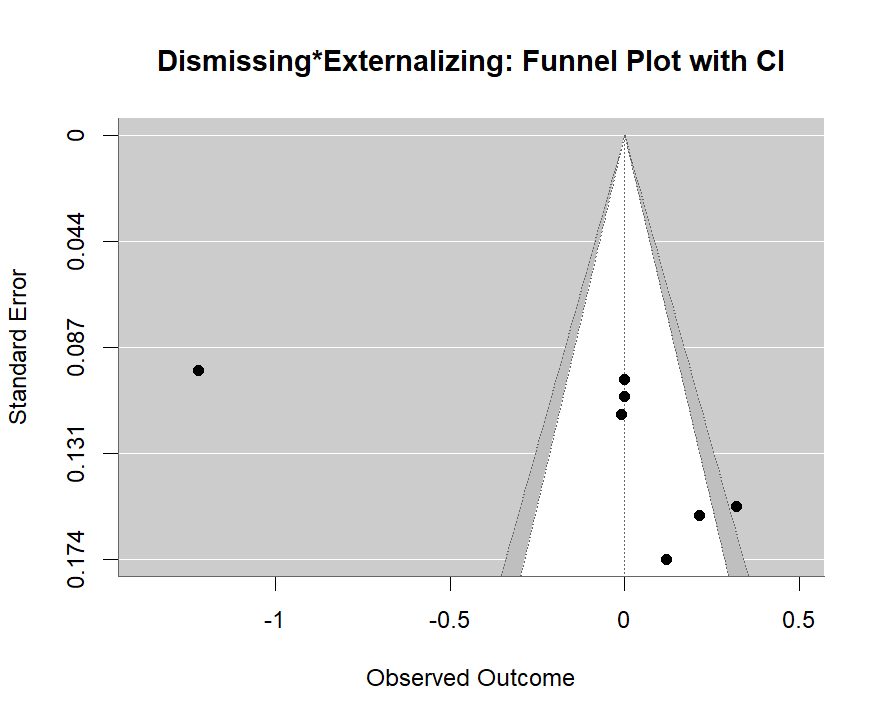


**III.3. Preoccupation:**

The meta-analysis yielded a almost null, non-significant negative effect (r = -.03, 95% CI [–.15, .07]). Heterogeneity was moderate (τ² = 0.01, I² = 33.9%), but the Q-test was not significant (Q(6) = 9, *p* = .170), indicating no between-study variability. Egger’s test did not detect funnel plot asymmetry (z = -0.87, p = .385). However, the trim-and-fill method impute two additional studies, correcting the ESs to r = -.01, 95% CI [–.10, .12], which remained non-significant.
Meta-regression (k = 7) indicated moderate residual heterogeneity (I² = 61.2%) not accounted for by the included moderators. The amount of heterogeneity explained was R² = 0%, and residual heterogeneity was not significant (QE(2) = 5.01, p =.08). None of the moderators—sample risk, gender, age, or quality—were significant (QM(4) = 2, p = .736).


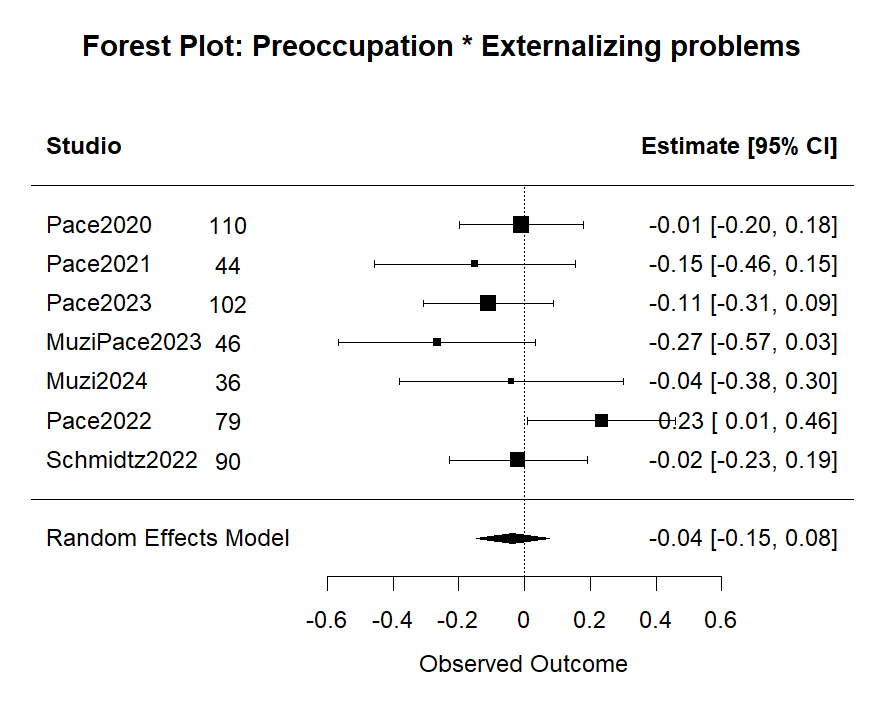

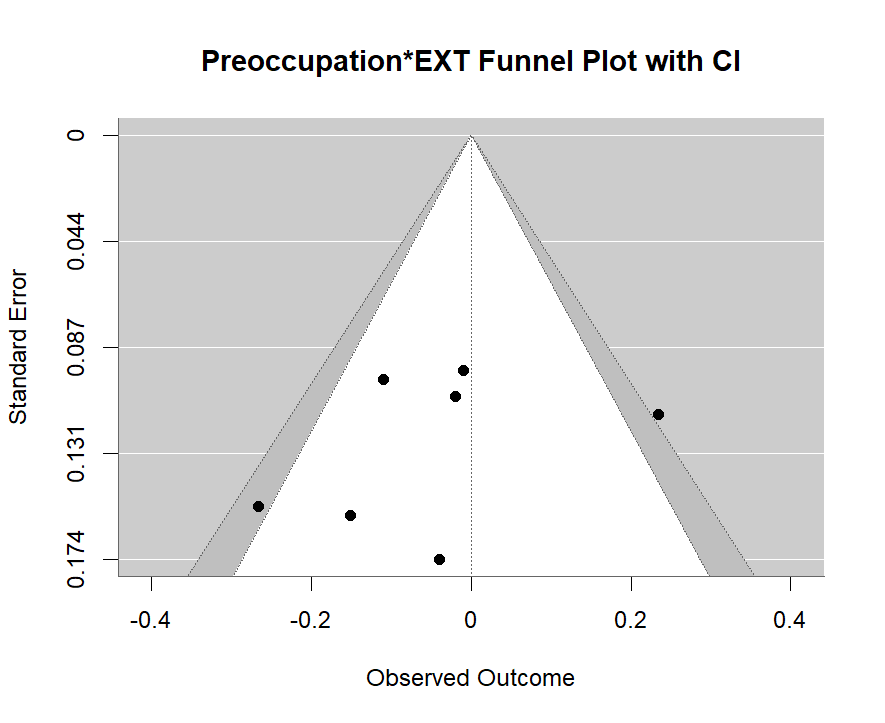


**III.4. Disorganization:**

The meta-analysis revealed a not significant negative effect (*r* = –.010, 95% CI [–.11, .09). There was no evidence of heterogeneity (τ² = 0, I² = 0%, Q(6) = 1.7, p = .88), suggesting that the effect sizes were consistent across studies. Egger’s test indicated no significant evidence of publication bias (z = 0.81, p = .42). The trim-and-fill procedure imputed two potentially missing studies, slightly adjusting the effect size to *r* = –.037 (95% CI [–.123, .050]). This adjusted estimate remains small and non-significant, further supporting the conclusion of no robust effect.
The mixed-effects *meta-regression* (k = 6) also showed no residual heterogeneity (τ² = 0, I² = 0%), and the model did not significantly explain additional variance (QM(4) = 1.46, p = .83). None of the tested moderators significantly influenced the effect size, all *p* >.63.


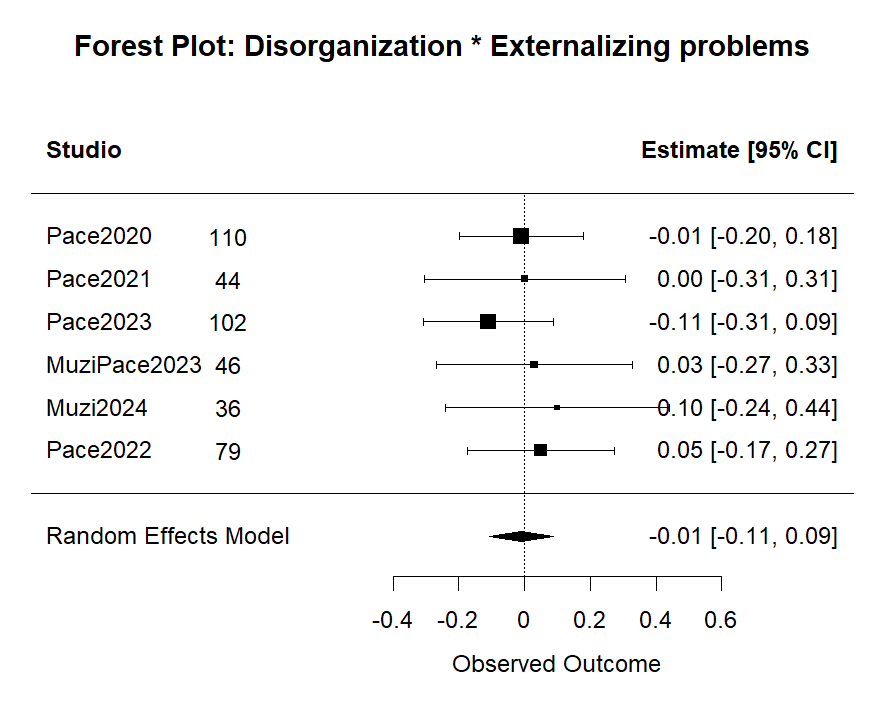

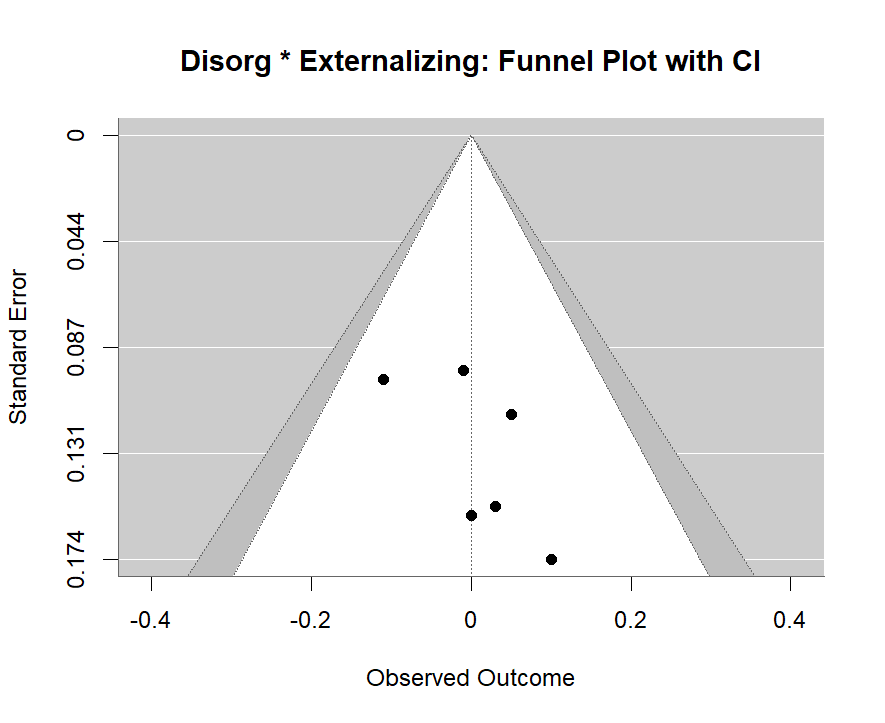


**III.5. Overall Coherence**

The meta-analysis revealed a small, non-significant negative effect size (*r* = -.015, 95% CI [–.087, .116]), indicating no evidence of association. Heterogeneity was low-to-moderate (*τ²* = 0.01, *I²* = 21%), with non-significant Q-test, *Q(6)* = 8.4, *p* = .21, suggesting between-study variability attributable to casual sampling error. Egger’s test showed no indication of publication bias (*z* = -0.92, *p* = .355). The trim-and-fill procedure imputed one missing study, adjusting the effect size to *r* = 0.04 (95% CI [-0.07, 0.15]), confirming no evidence of association.
***Meta-regression*** *(k* = 7) indicated moderate residual heterogeneity (*I²* = 51%) not accounted for by the included moderators. The amount of heterogeneity explained was R² = 0%, and residual heterogeneity was not significant (*QE*(2) = 3.71, *p* = .157). None of the moderators—sample risk, gender, age, or quality—were significant (*QM*(4) = 3.34, *p* = .502).


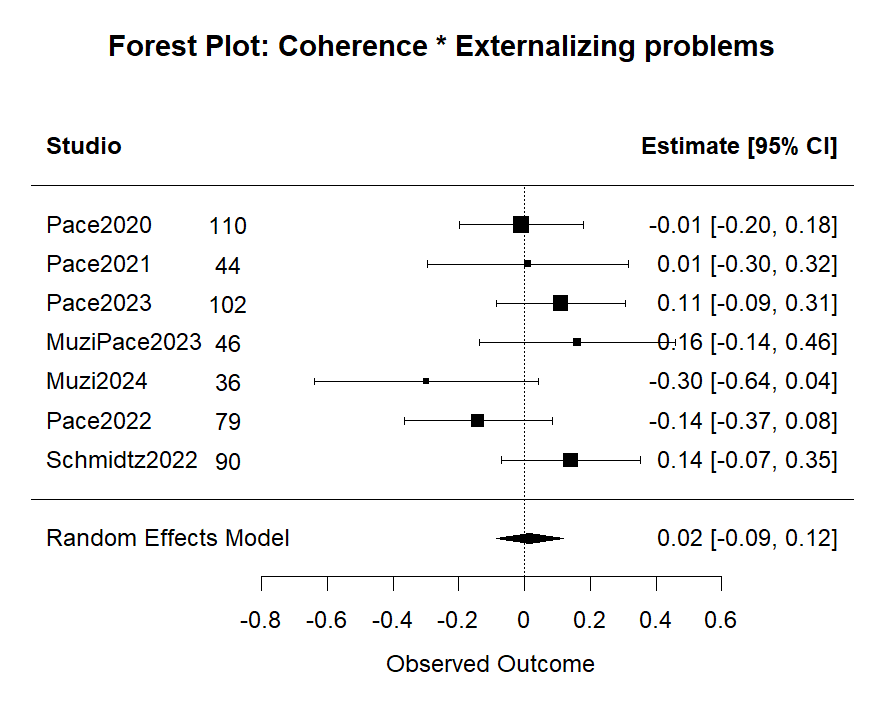

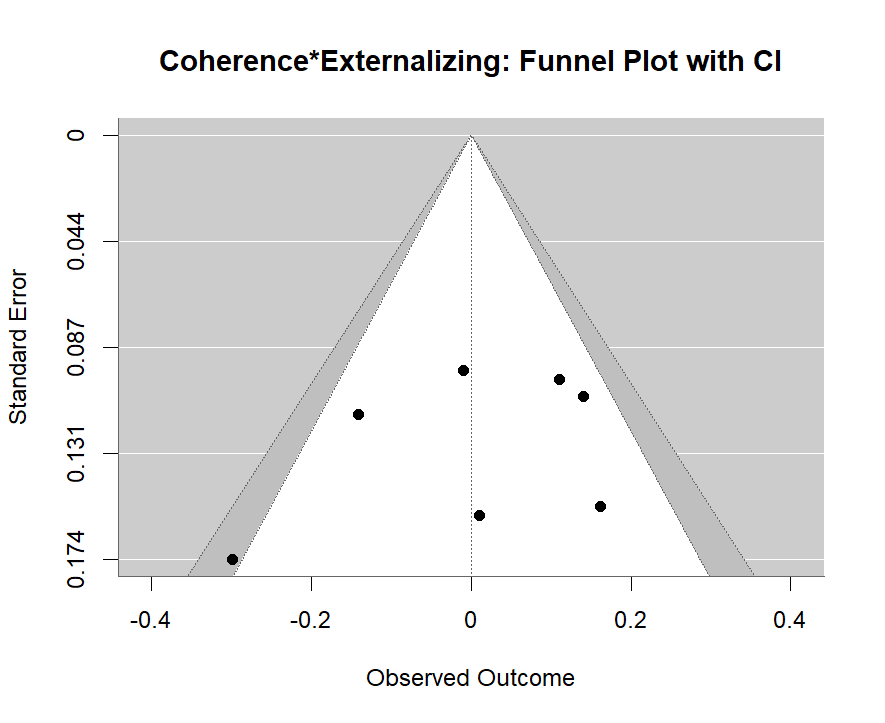


**III.6. SB/SH mother**:

The meta-analysis revealed a small, non-significant negative effect size (r = -0.08, 95% CI [–.18, .02]), indicating no evidence of association. Heterogeneity was low-to-moderate (*τ²* = 0.01, *I²* = 26.5%), with non-significant Q-test, *Q(6)* = 7.8, *p* = .25, suggesting between-study variability attributable to casual sampling error. Egger’s test showed no indication of publication bias (*z* = -1.12, *p* = .264). The trim-and-fill procedure imputed one missing study, adjusting the effect size to *r* = -0.06 (95% CI [-0.16, 0.04]), confirming no evidence of association.
***Meta-regression*** *(k* = 7) indicated no residual heterogeneity (*I²* = 0%) not accounted for by the included moderators. The amount of heterogeneity explained was R² = 0%, and residual heterogeneity was not significant (*QE*(2) = 1.11, *p* = 0.57). None of the moderators—sample risk, gender, age, or quality—were significant (*QM*(4) = 2.20, *p* = .698).


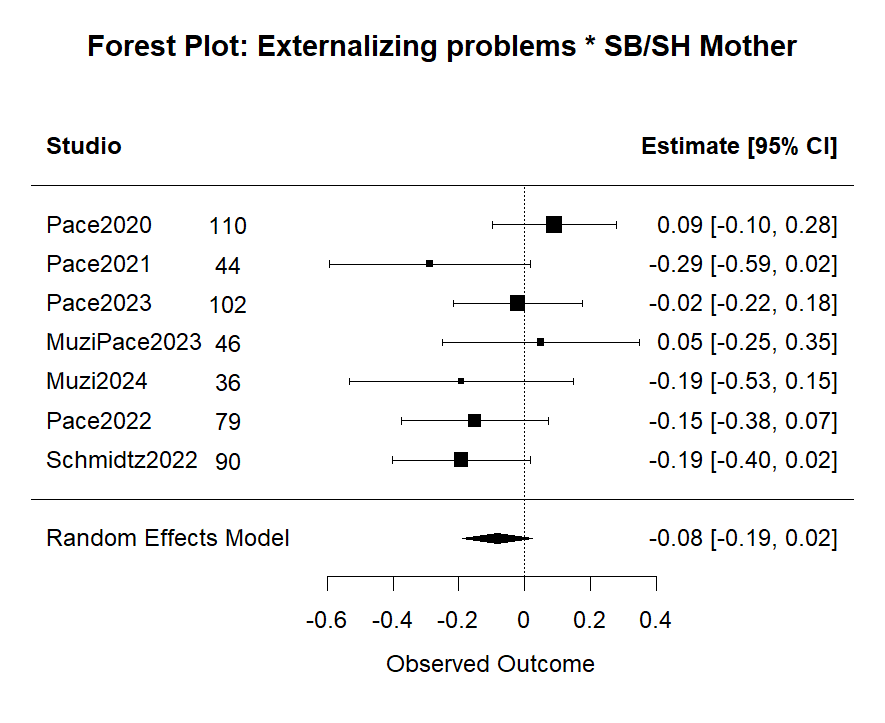

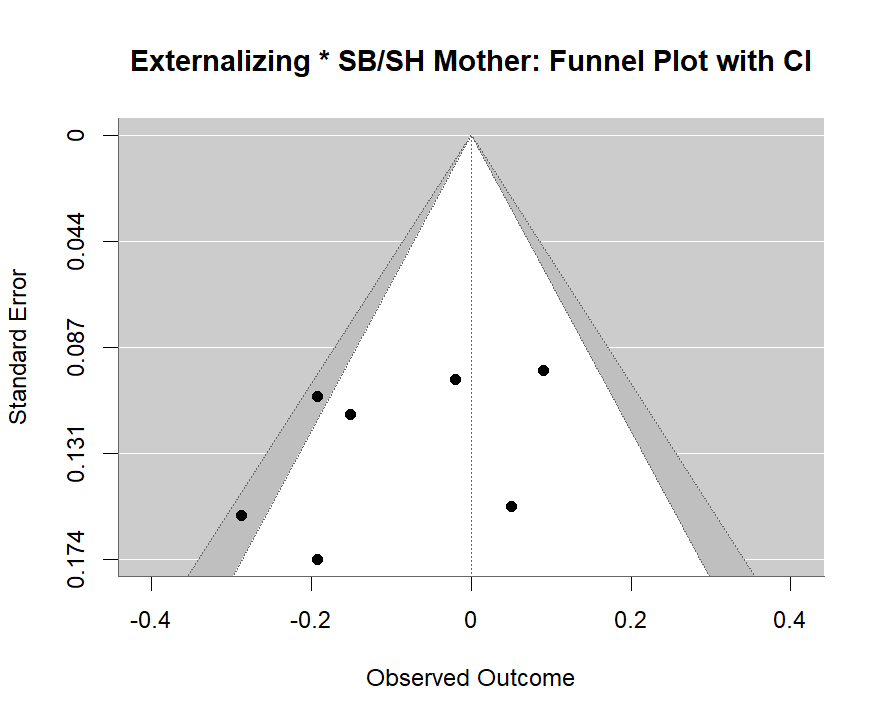


**III.7. SB/SH father:**

The meta-analysis revealed a non-significant effect size (*r* = .05, 95% CI [–.20, .10]), indicating no evidence of association. Heterogeneity was moderate (*τ²* = 0.03, *I²* = 63%), with significant Q-test, *Q(6)* = 16.7, *p* = .01, suggesting not casual between-study variability that justified the use of a robust random model. However, Egger’s test did not suggested publication bias (*z* = 0.67, *p* = .502). The trim-and-fill procedure imputed no missing study, suggesting robust results not affected by publication biases, and the adjusted effect size remained the same indeed, *r* = 0.05 (95% CI [–.20, .102]), confirming no evidence of association.
***Meta-regression*** *(k* = 7) indicated moderate residual heterogeneity (*I²* = 54%), not accounted for by the included moderators, *QM*(4) = 5.95, *p* = .203. The amount of heterogeneity explained was R² = 42%, with significant amount of residual heterogeneity remained unexplained, *QE*(2) = 4.34, *p* = .114). However, beta coefficients indicated gender as significant moderator, B = 0.02, p = .031 (.002, .037).

**
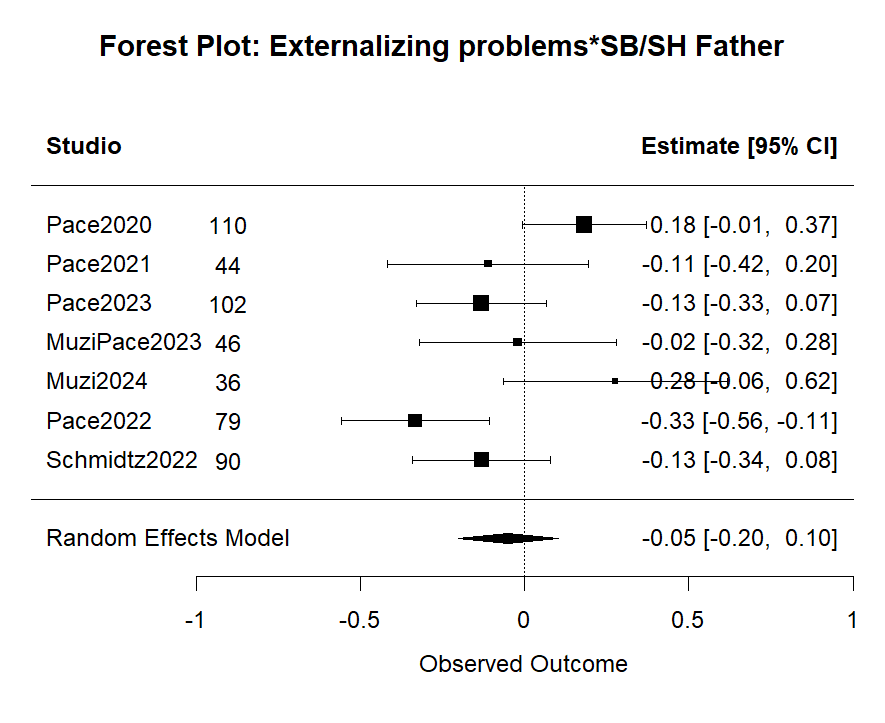

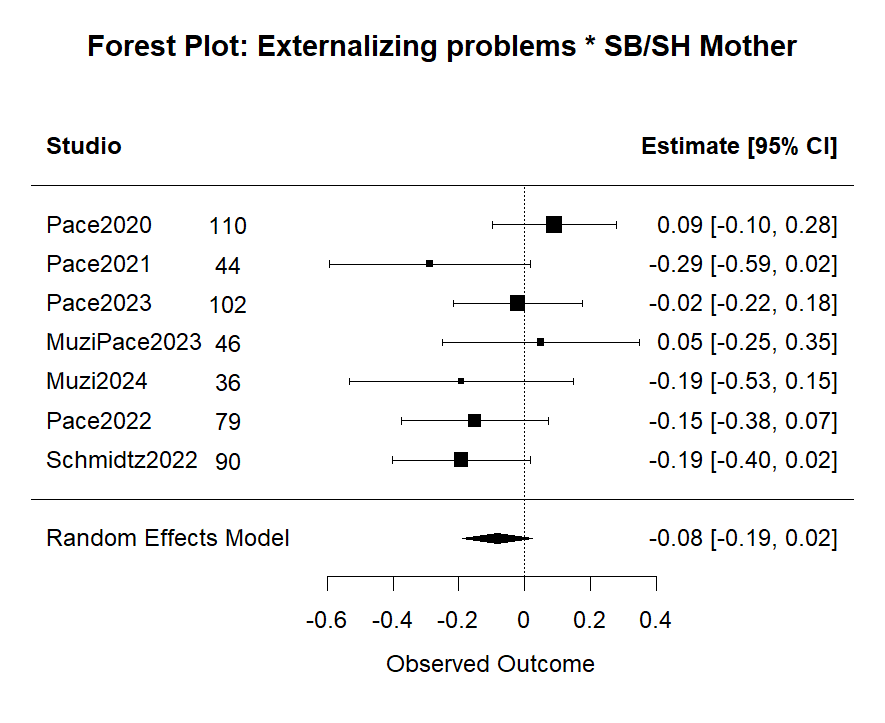
**

**IV. Other problems**

**IVa. Attentional problems.**

**IVa.1. Security:**

The aggregated correlation with security indicated non-significant negative effect (*r* = –0.04, 95% CI [–0.12, 0.05]). There was no heterogeneity (*τ²* = 0, *I²* = 0%), with a non-significant Q-test, Q(6) = 3.3, *p* = .77, suggesting no between-study variability. Egger’s test indicated no potential publication bias (*z* = –0.43, *p* = .668). The trim-and-fill procedure imputed two missing samples, adjusting the effect to *r* = .001 (95% CI [–.08, .08]).
***Meta-regression*** (*k* = 7) explained all residual heterogeneity (*R*² = 100%), with no significant residual heterogeneity left (*QE*(2) = 2.01, *p* = .36). Still, none of the moderators—sample risk, gender, age, or quality—were significant predictors of the effect size (*QM*(4) = 1.26, *p* = .869).


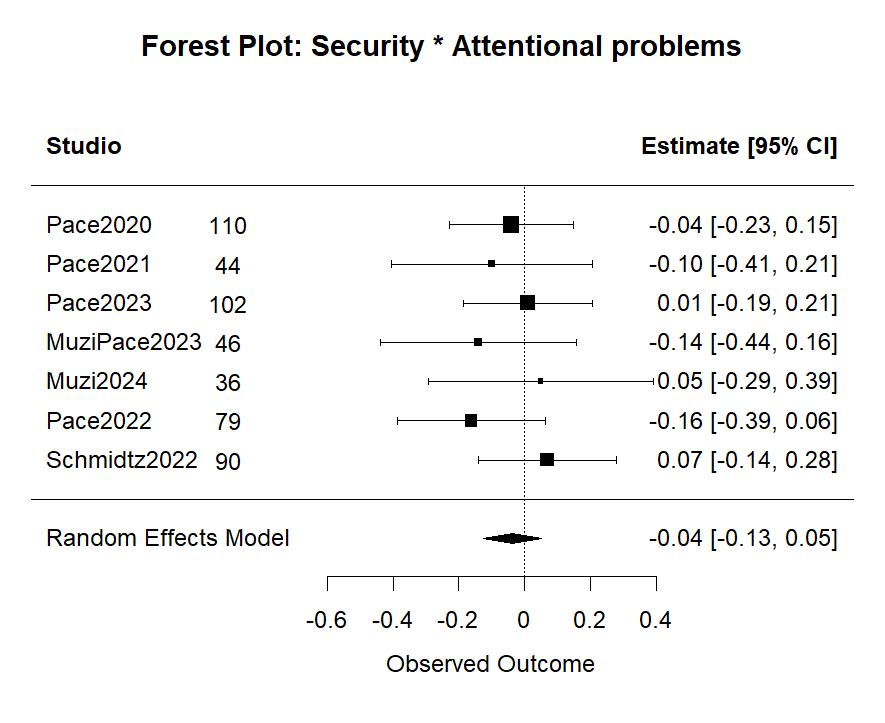

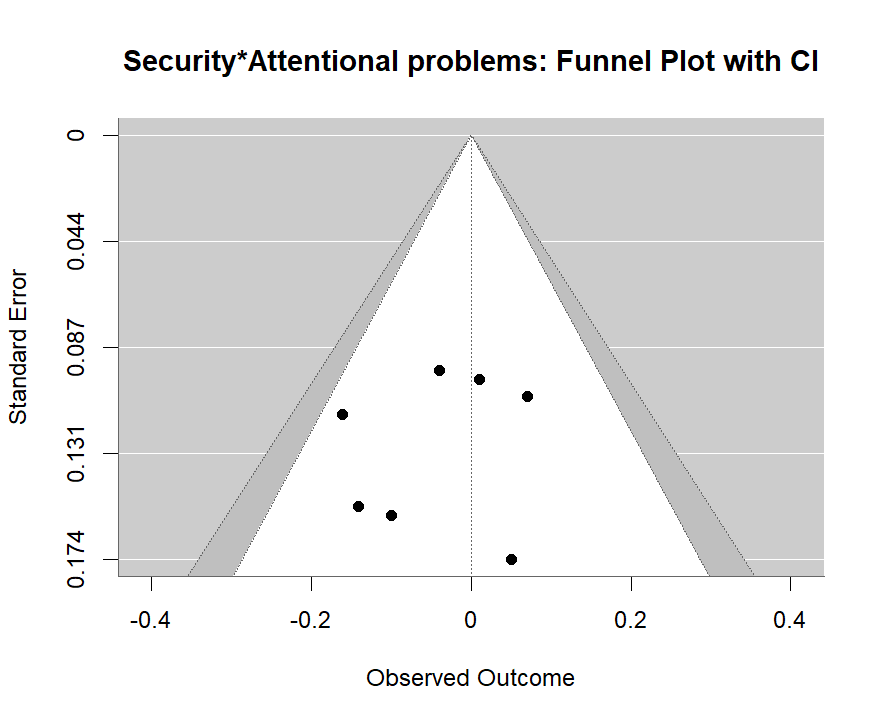


**IVa.2. Dismissing:**

The meta-analysis revealed a small, non-significant negative effect size (*r* = –.01, 95% CI [–.09, .09]). No between-study heterogeneity was detected, *τ²* = 0, *I²* = 0%), *Q(6)* = 6.2, *p* = .04, and Egger’s test results did not indicate publication bias (*z* = -0.40, *p* = .685). The trim-and-fill procedure imputed one missing study, but the adjusted effect size was still negligible, *r* = 0.01 (95% CI [-0.07, 0.10]).
***Meta-regression*** *(k* = 7) explained low residual heterogeneity (*R*² = 24.74%), with no significant residual heterogeneity left (*QE*(2) = 2.10, *p* = .349). Still, none of the moderators—sample risk, gender, age, or quality—were significant predictors of the effect size (*QM*(4) = 4.11, *p* = .390).


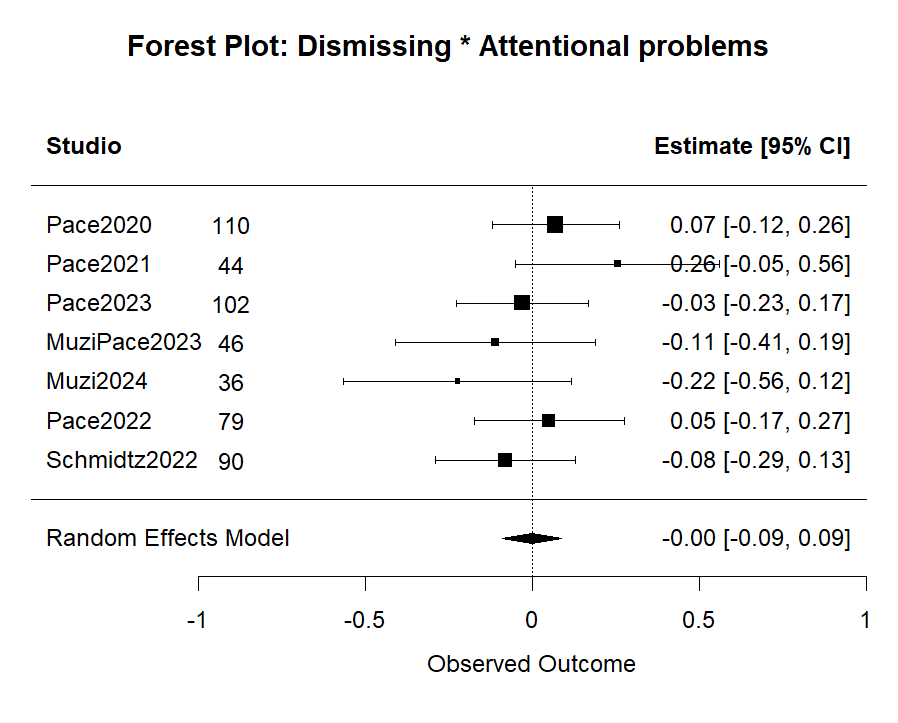

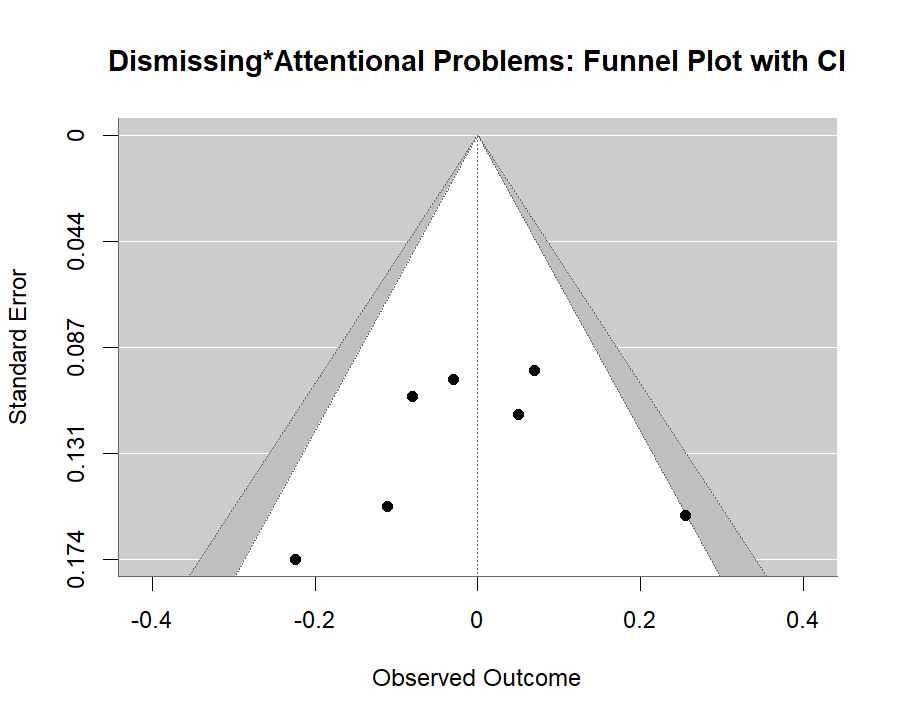


**IVa.3. Preoccupation:**

The aggregated correlation with preoccupation indicated non-significant effect (r = 0.06, 95% CI [–0.02, 0.15]). Heterogeneity was low (τ² = 0.01, I² = 3.8%), with a non-significant Q-test, Q(6) = 5.7, p = .46, suggesting no between-study variability. Egger’s test indicated no potential publication bias (z = 1.70, p = .088). The trim-and-fill procedure imputed two missing samples, adjusting the effect to r = .029 (95% CI [–.06, .12]).
***Meta-regression*** (k = 7) explained all residual heterogeneity (R² = 100%), with no significant residual heterogeneity left (QE(2) = 0.23, p = .891). Sample risk was a significant predictor, B = 0.28, p = .027 (.032, .527) moderators, while the model was not significant (QM(4) = 5.46, p = .244).


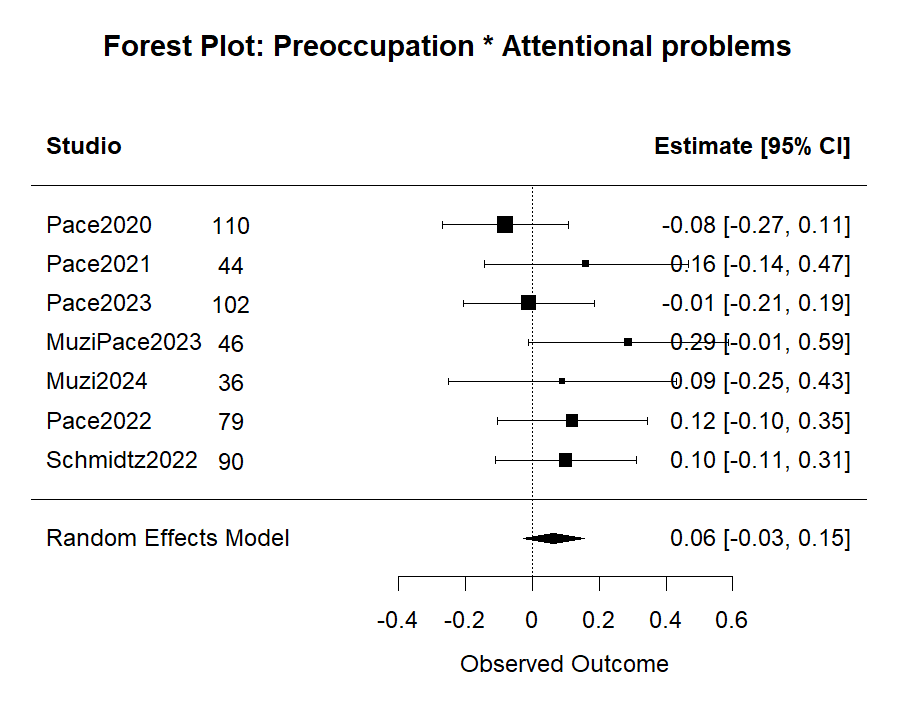

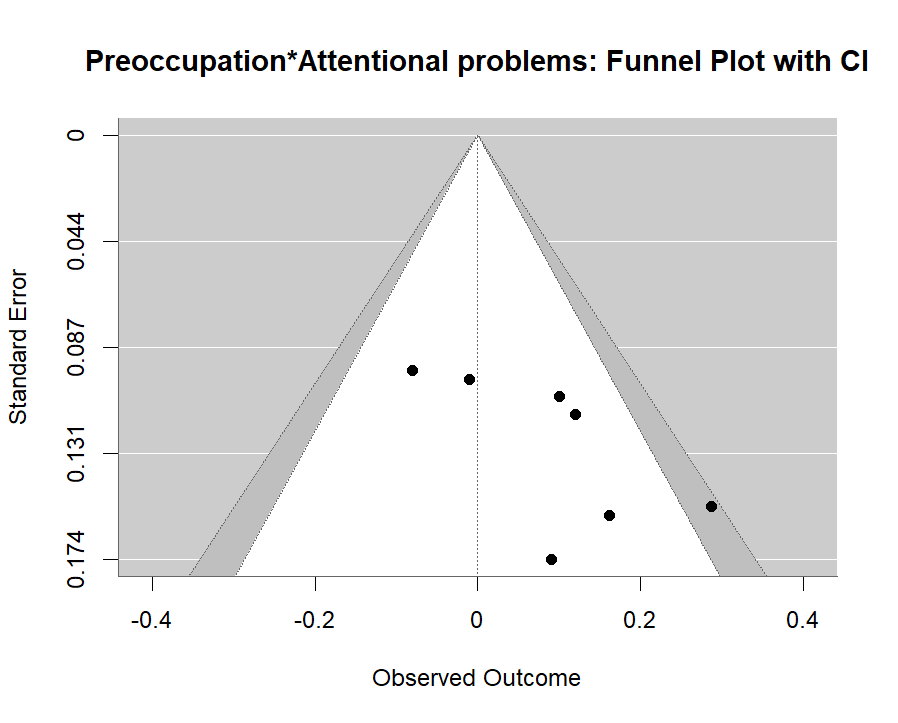


**IVa.4. Disorganization:**

The meta-analysis revealed a not significant effect (*r* = .01, 95% CI [–.10, .10). There was no evidence of heterogeneity (τ² = 0, I² = 0%, Q(6) = 1.7, p = .89), suggesting that the effect sizes were consistent across studies. Egger’s test indicated no significant evidence of publication bias (z = -0.19, p = .84). The trim-and-fill procedure imputed one potentially missing study, slightly adjusting the effect size to *r* = .01 (95% CI [–.08, .10]). This adjusted estimate remains small and non-significant, further supporting the conclusion of no robust effect.
The mixed-effects *meta-regression* (k = 6) also showed no residual heterogeneity (τ² = 0, I² = 0%), and the model did not significantly explain additional variance (QM(4) = 1.56, p = .81). None of the tested moderators significantly influenced the effect size, all *p* >.31.


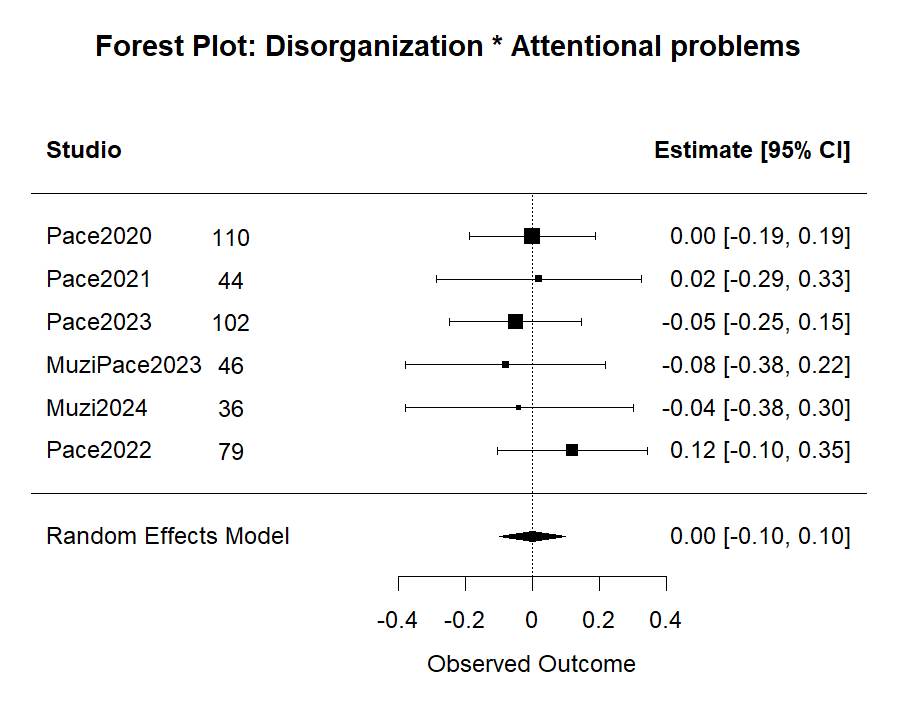

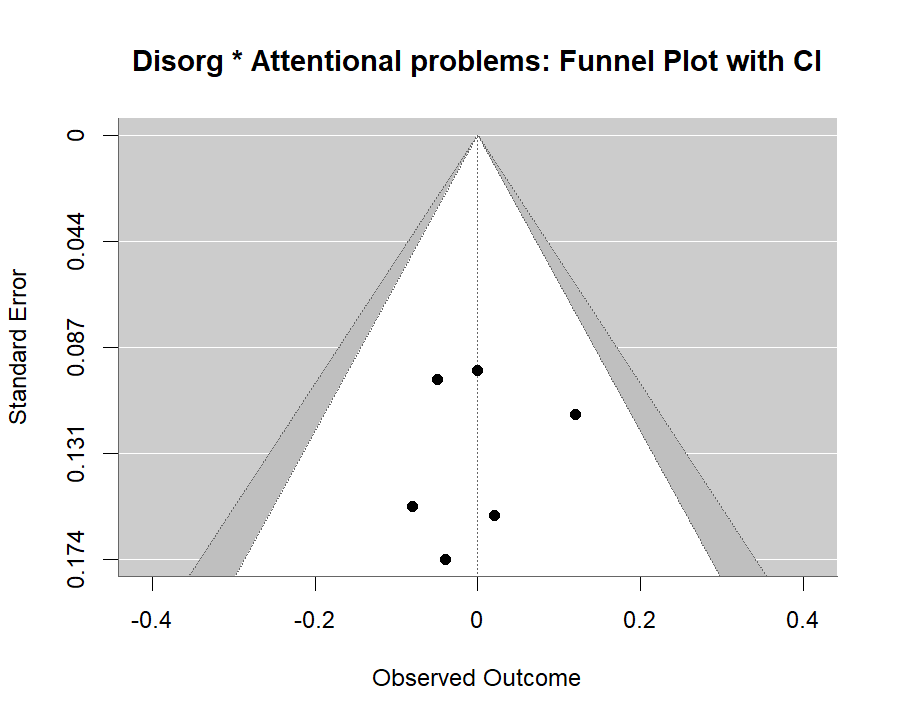


**IVa.5. Overall Coherence:**

The meta-analysis revealed a small, non-significant negative effect size (*r* = -.02, 95% CI [–.11, .06]), indicating no evidence of association. Heterogeneity was low-to-moderate (*τ²* = 0, *I²* = 0%), with non-significant Q-test, *Q(6)* = 3.8, *p* = .71, suggesting between-study variability attributable to casual sampling error. Egger’s test showed no indication of publication bias (*z* = -0.16, *p* = .874). The trim-and-fill procedure imputed one missing study, adjusting the effect size to *r* = -0.02 (95% CI [-0.11, 0.06]), confirming no evidence of association.
The mixed-effects *meta-regression (k = 6)* also showed no residual heterogeneity (τ² = 0, I² = 0%), and the model did not significantly explain additional variance (QM(4) = 1.69, p = .79). None of the tested moderators significantly influenced the effect size, all p >.34.


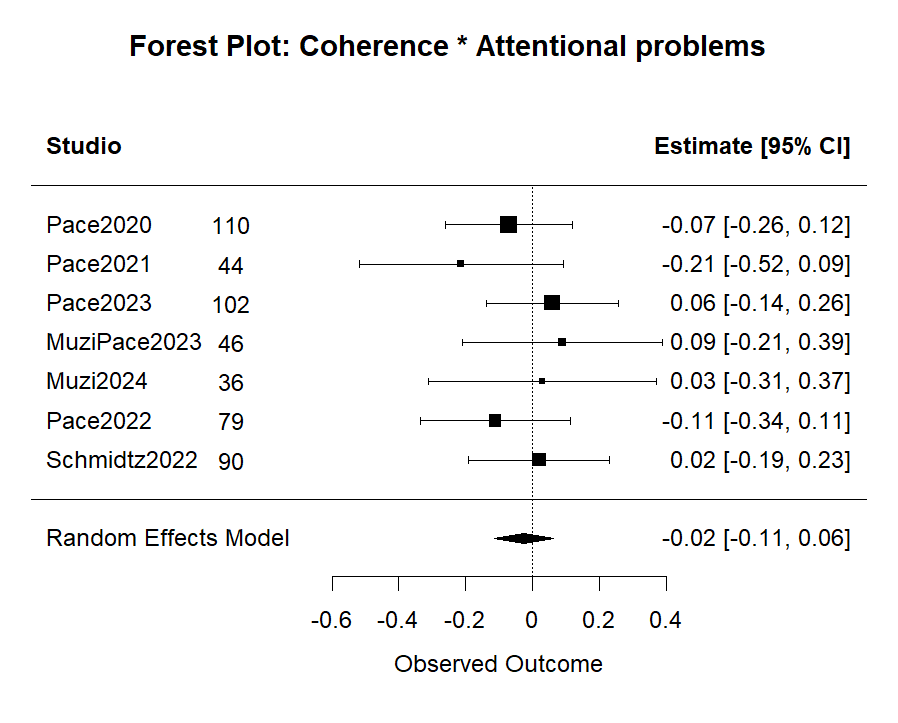

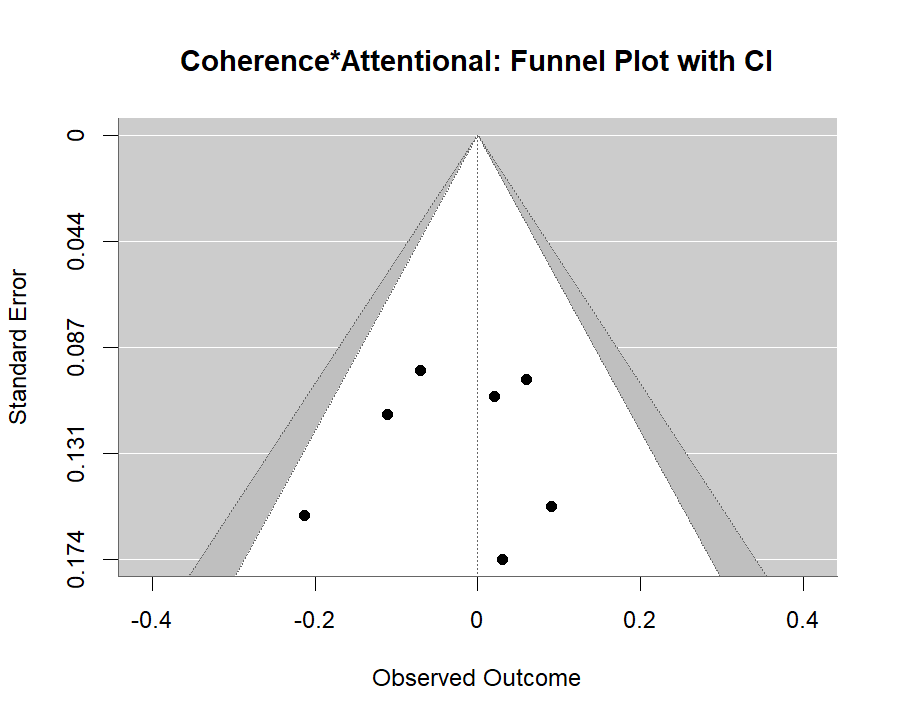


**IVa.6. SB/SH mother**:

The meta-analysis revealed a small, non-significant negative effect size (*r* = -0.08, 95% CI [–.17, .01]), indicating no evidence of association. Heterogeneity was null (*τ²* = 0.0, *I²* = 0%), with non-significant Q-test, *Q(6)* = 3.1, *p* = .790, suggesting between-study variability attributable to casual sampling error. Egger’s test showed no indication of publication bias (*z* = 0.47, *p* = .634). The trim-and-fill procedure imputed one missing study, adjusting the effect size to *r* = -0.10 (95% CI [-0.18, -0.018]), confirming no evidence of association.
***Meta-regression*** *(k* = 7) indicated no residual heterogeneity (*I²* = 0%) not accounted for by the included moderators. The amount of heterogeneity explained was R² = 0%, and residual heterogeneity was not significant (*QE*(2) = 1.30, *p* = 0.52). None of the moderators—sample risk, gender, age, or quality—were significant (*QM*(4) = 1.82, *p* = .769).


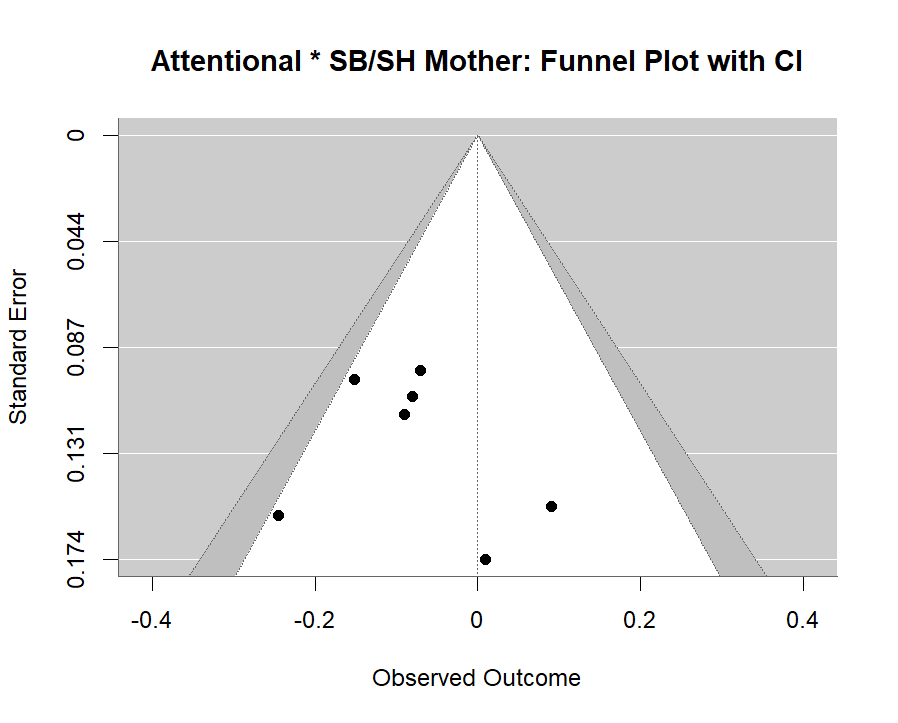

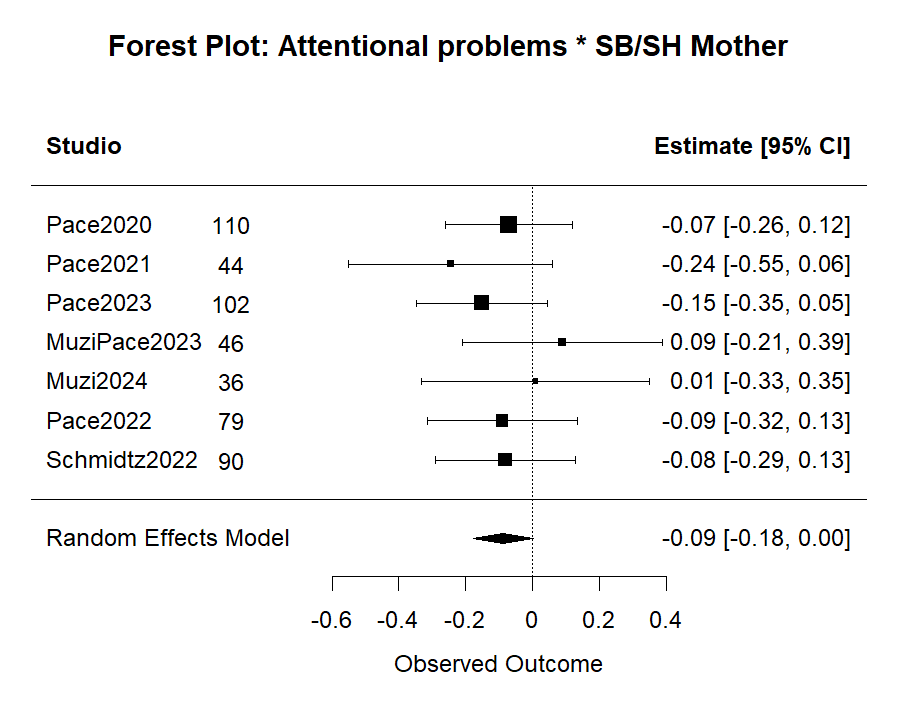


**IVa.7. SB/SH father:**

The meta-analysis revealed a non-significant negative effect size (*r* = -.04, 95% CI [–.15, .06]), indicating no evidence of association. Heterogeneity was low-to-moderate (*τ²* = 0.01, *I²* = 27.2%), with non-significant Q-test, *Q(6)* = 9, *p* = .18. Egger’s test did not suggested publication bias (*z* = 1.47, *p* = .140). The trim-and-fill procedure imputed no missing study, suggesting robust results not affected by publication biases, and the adjusted effect size remained the same indeed, *r* = 0.04 (95% CI [–.15, .06]), confirming no evidence of association.
The mixed-effects ***meta-regression*** (k = 6) also showed no residual heterogeneity (τ² = 0, I² = 0%), and the model did not significantly explain additional variance (QM(4) = 7.47, p = .113). However, beta coefficients indicated gender B = 0.01, p = .030 (.001,.028), and study quality B = 0.74, p = .029 (.074, 1.400) as significant moderators.


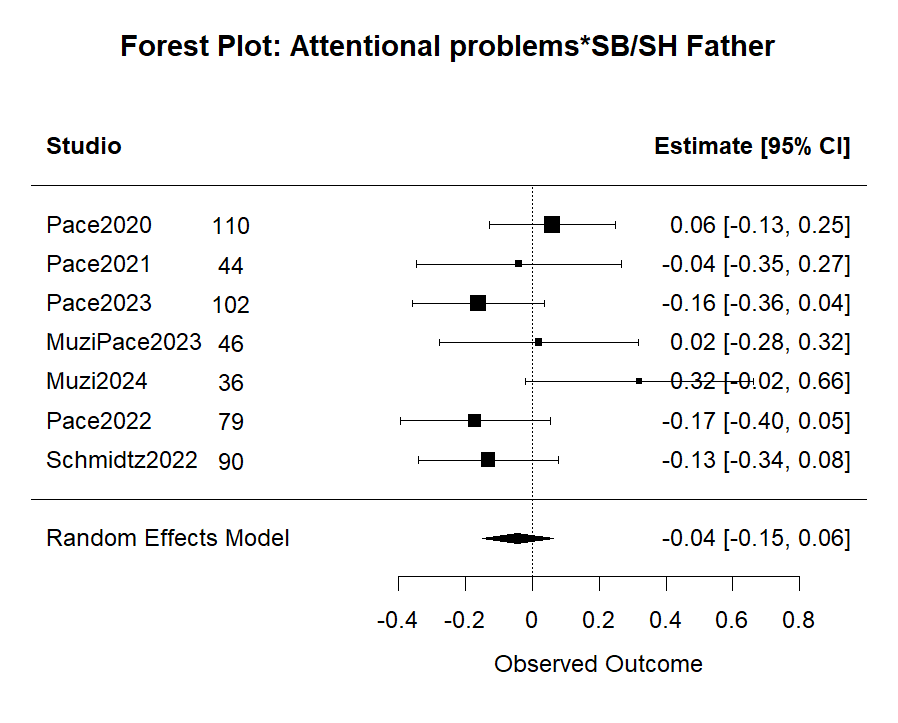

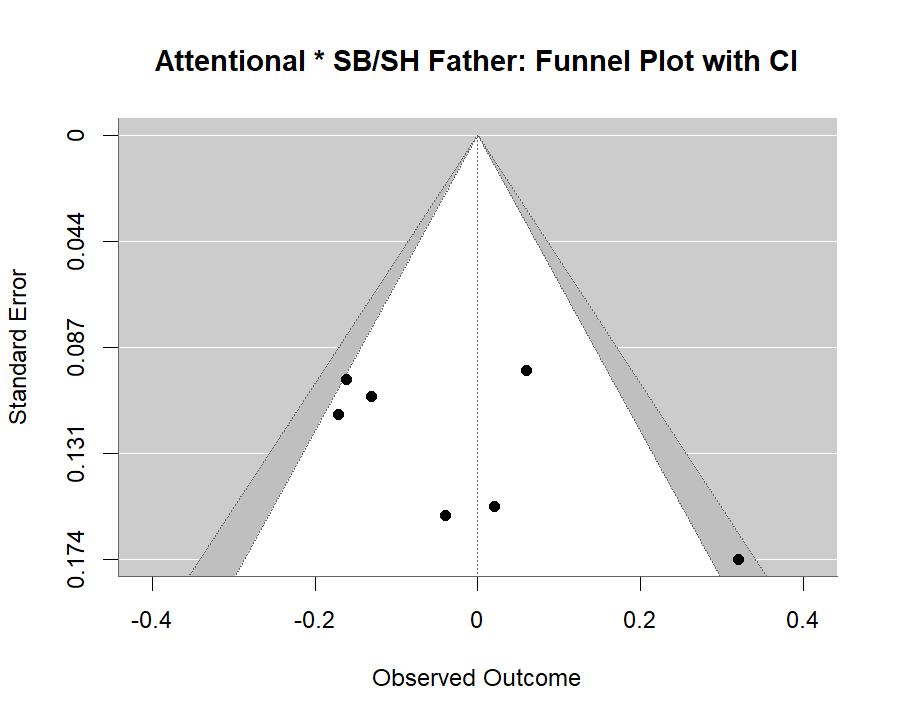


**IVb. Social problems.**

**IVb.1. Security:**

The aggregated correlation with security indicated non-significant negative effect (*r* = –0.12, 95% CI [–0.20, -0.28]). There was no heterogeneity (*τ²* = 0, *I²* = 0%), with a non-significant Q-test, Q(6) = 4.6, *p* = .600, suggesting no between-study variability. Egger’s test indicated no potential publication bias (*z* = –0.94, *p* = .348). The trim-and-fill procedure imputed three missing samples, adjusting the effect to lower *r* = -0.05 (95% CI [–.14, -.04]).
***Meta-regression*** (*k* = 7) showed no residual heterogeneity to explain, *τ²* = 0, *R*² = 0.0%, *QE*(2) = 0.72, *p* = .697). None of the moderators—sample risk, gender, age, or quality—were significant predictors of the effect size (*QM*(4) = 3.87, *p* = .424).

**
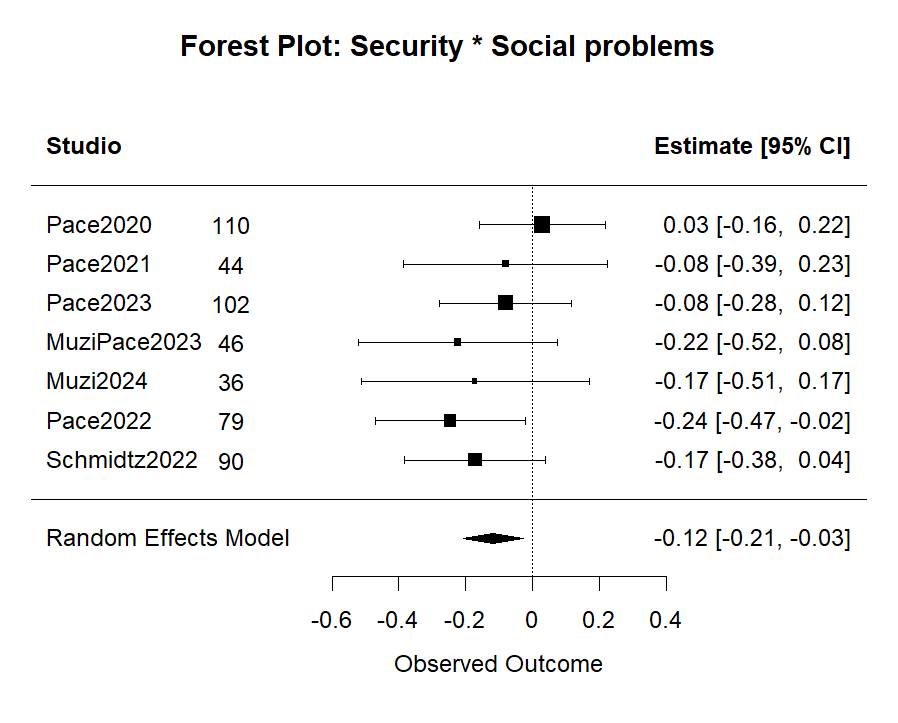

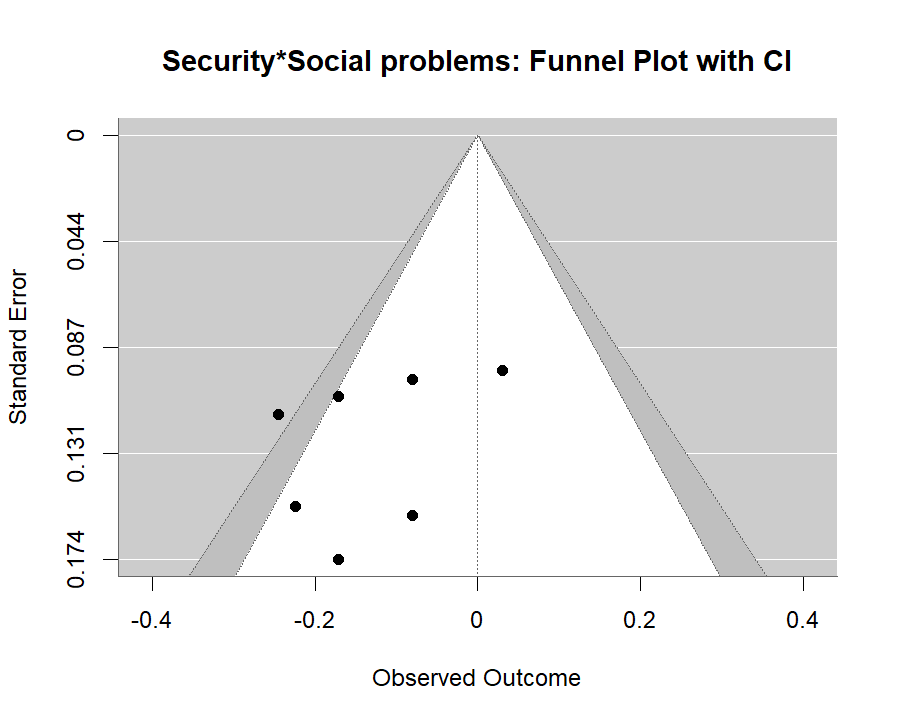
**

**IVb.2. Dismissing:**

The meta-analysis revealed a small, non-significant negative effect size (*r* = –0.03, 95% CI [–0.16, 0.10]). Moderate between-study heterogeneity was detected, *τ²* = .01, *I²* = 51%), with not significant Q test, *Q(6)* = 12.2, *p* = .058, and Egger’s test results did not indicate publication bias (*z* = -0.05, *p* = .961). The trim-and-fill procedure imputed no missing study, and the effect size remained negligible, *r* = -0.02 (95% CI [-0.15, -0.10]).
***Meta-regression*** *(k* = 7) revealed high residual heterogeneity (τ² = 0.0447, I² = 79.2%), with a significant Q-test (QE(2) = 8.73, p = .013), indicating notable between-study variability. The model explained none of this heterogeneity (R² = 0%), and the test of moderators was non-significant (QM(4) = 1.57, p = .814). None of the individual predictors (sample risk, gender, age, quality) significantly moderate the effect. The overall effect estimate was also non-significant (estimate = 1.57, p = .790, 95% CI [–10.01, 13.15]).


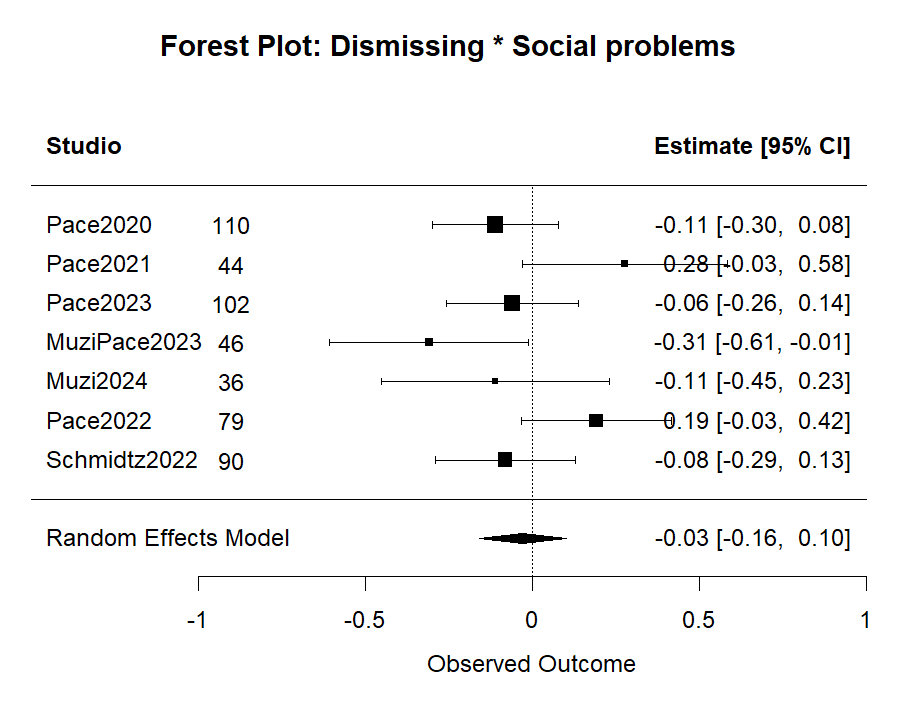

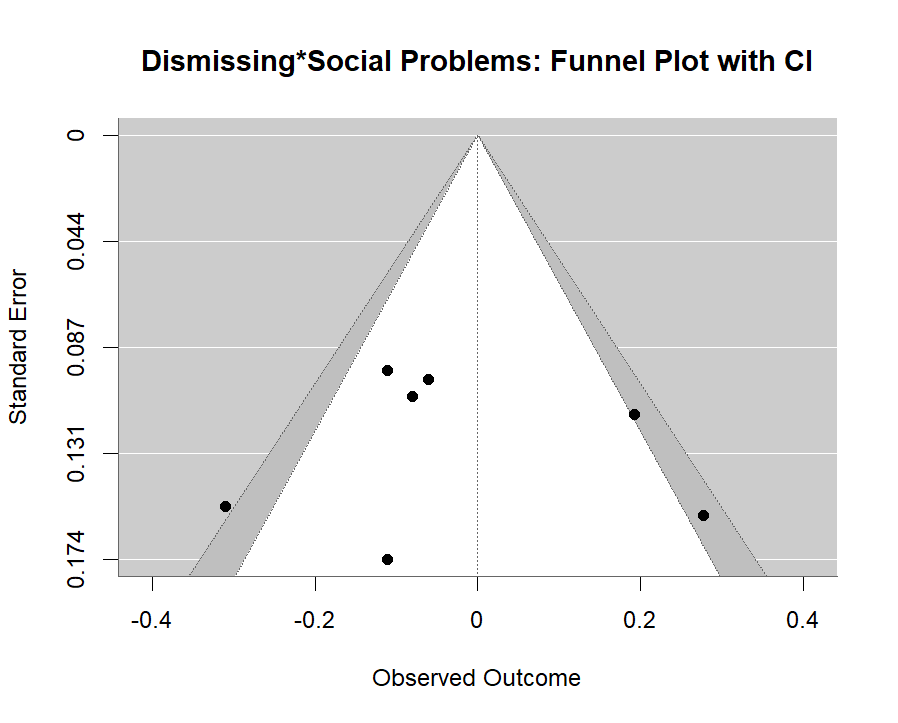


**IVb.3. Preoccupation:**

The aggregated correlation with preoccupation indicated non-significant effect (r = 0.12, 95% CI [–0.04, 0.21]). There was no heterogeneity (τ² = 0, I² =0%), with a non-significant Q-test, Q(6) = 4.6, p = .600, suggesting no between-study variability. Egger’s test indicated no potential publication bias (z = 0.96, p = .334). The trim-and-fill procedure imputed two missing samples, adjusting the effect to r = .12 (95% CI [.04, .21]).
A mixed-effects ***meta-regression (k = 7)*** showed no residual heterogeneity (τ² = 0, I² = 0%), with a non-significant Q-test (QE(2) = 0.31, p = .857), indicating consistency across studies. The model did not explain any additional variance (R² = 0%), and the test of moderators was also non-significant (QM(4) = 2.09, p = .718), with none of the individual moderators significantly predicted effect sizes.


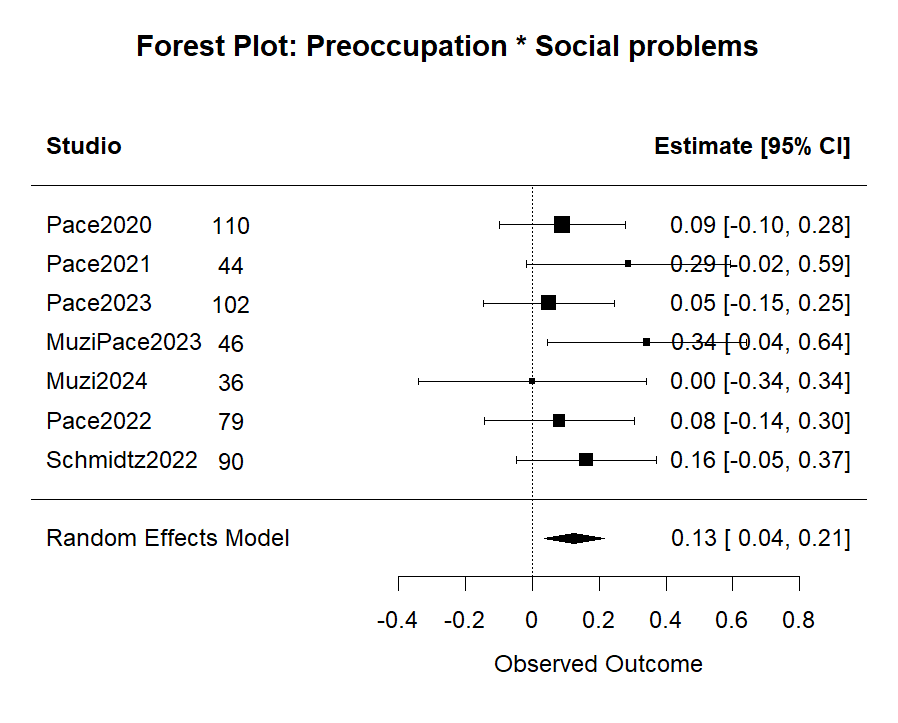

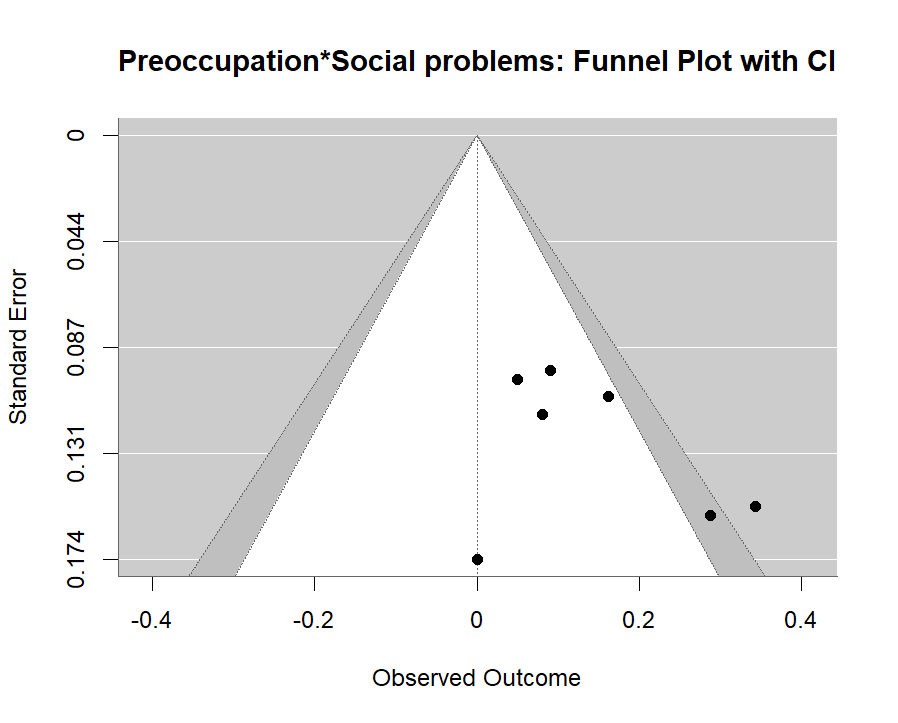


**IVb.4. Disorganization:**

The meta-analysis revealed a not significant effect (*r* = .12, 95% CI [0.01, 0.23]). There was low heterogeneity (τ² = 0.01, I² = 26.3%) with non-significant Q test, Q(6) = 6.4, p = .27), suggesting that the effect sizes were consistent across studies. Egger’s test indicated no significant evidence of publication bias (*z* = 0.11, *p* = .91). The trim-and-fill procedure imputed one potentially missing study, slightly adjusting the effect size to r = 0.12 (95% CI [0.01, 0.23]). This adjusted estimate remains small and non-significant, further supporting the conclusion of no robust effect.
A mixed-effects ***meta-regression*** model (*k* = 6) revealed no residual heterogeneity (τ² = 0, I² = 0%), suggesting high consistency among studies. The test for residual heterogeneity was not significant (QE(1) = 3.34, p = .068), but not explained model heterogeneity (R² = 0%) which was substantial, I^2^= 70%. The overall test of moderators was non-significant (QM(4) = 1.14, p = .887), but none of moderators was significant, all *p* <.440.


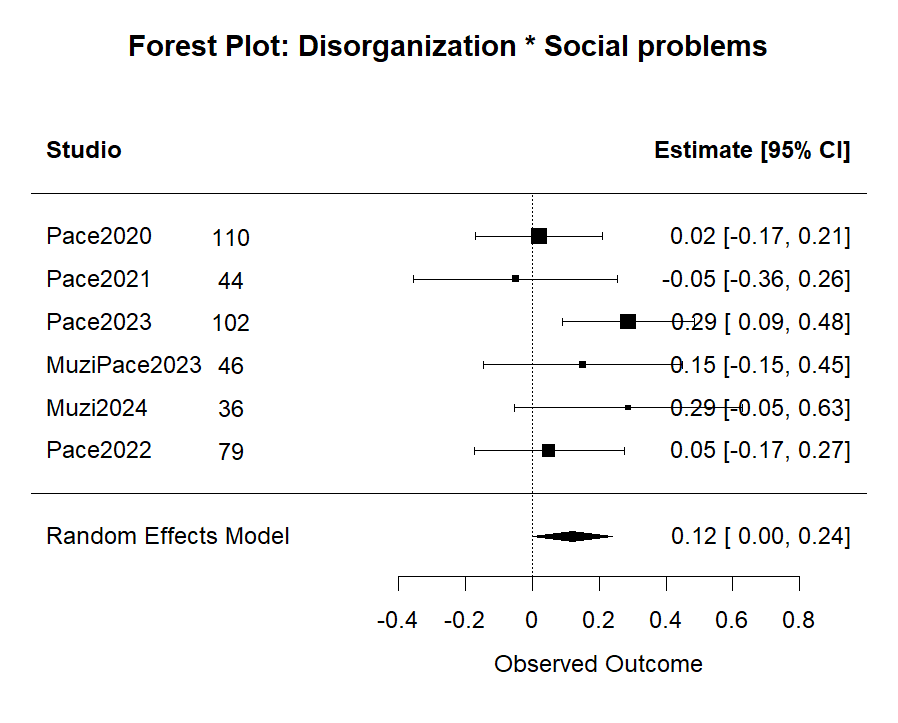

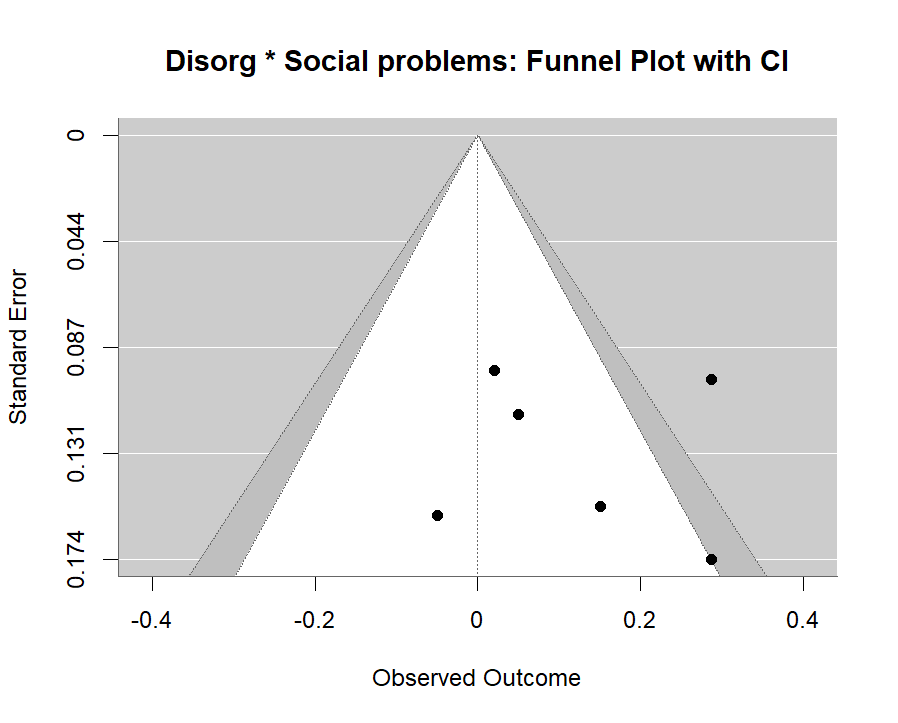


**IVb.5. Overall Coherence:**

The meta-analysis revealed a small, non-significant negative effect size (*r* = -0.08, 95% CI [–0.17, 0.01]), indicating no evidence of association. There was no heterogeneity (*τ²* = 0, *I²* = 0%), with non-significant Q-test, *Q(6)* = 5.4, *p* = .49, suggesting between-study variability attributable to casual sampling error. Egger’s test showed no indication of publication bias (*z* = -0.93, *p* = .350). The trim-and-fill procedure imputed one missing study, adjusting the effect size to *r* = -0.06 (95% CI [-0.15, 0.03]), confirming no evidence of association.
The mixed-effects *meta-regression (k = 6)* also showed no residual heterogeneity (τ² = 0, I² = 0%), and the model did not significantly explain additional variance (QM(4) = 4.14, p = .387). None of the tested moderators significantly influenced the effect size, all p >.100.


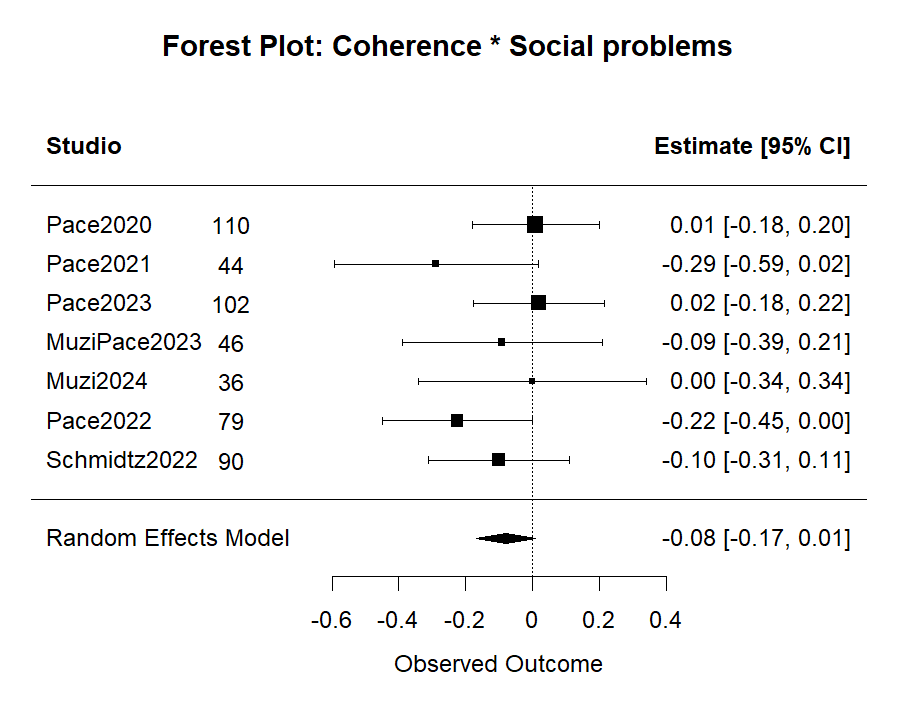

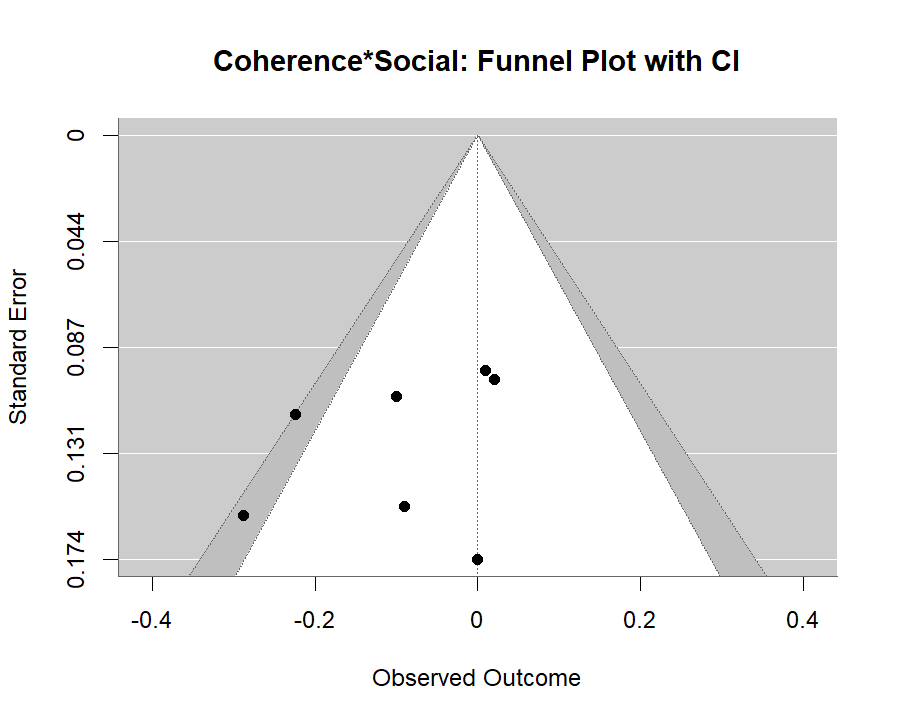


**IVb.6. SB/SH mother**:

The random-effects meta-analysis of seven studies revealed a small, non-significant negative association between maternal self-injury/substance use and children's social problems (r = –0.079, 95% CI: –0.187 to 0.032). Heterogeneity was low-to-moderate (τ² = 0.007, I² = 32.5%, Q(6) = 8.0, p = .24), and no evidence of publication bias was detected (Egger’s test p = .531). The Trim and Fill method suggested the imputation of two potentially missing studies, slightly attenuating the effect (r = –0.026, 95% CI: –0.134 to 0.084).
***Meta-regression*** showed no significant moderation by sample type, gender, age, or study quality (QM(4) = 0.87, p = .928), and residual heterogeneity was not significant (QE(2) = 5.38, p = .068), indicating other unexplained sources of variability.


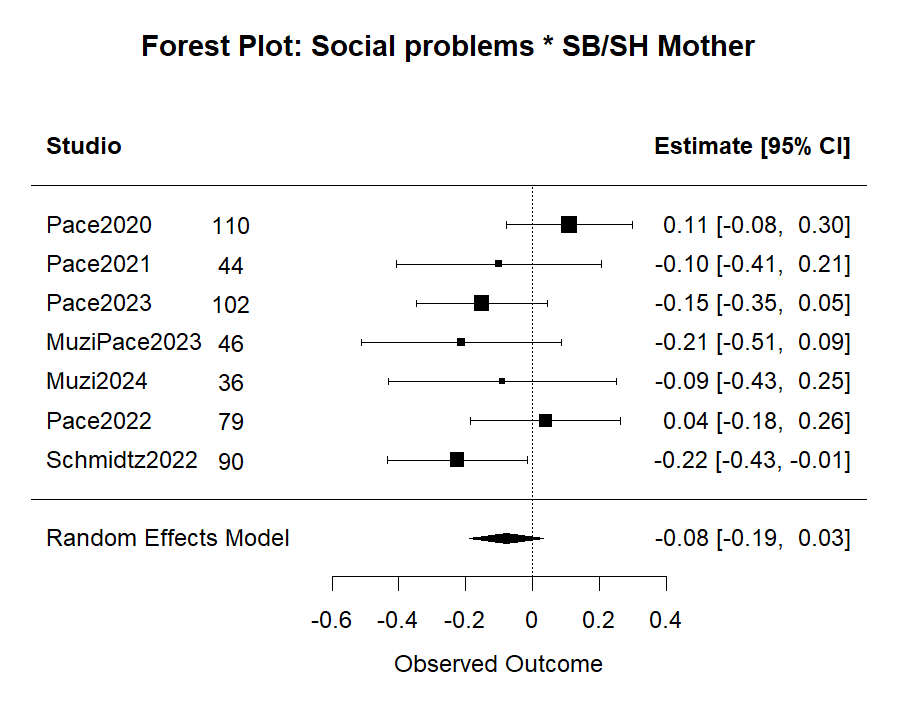

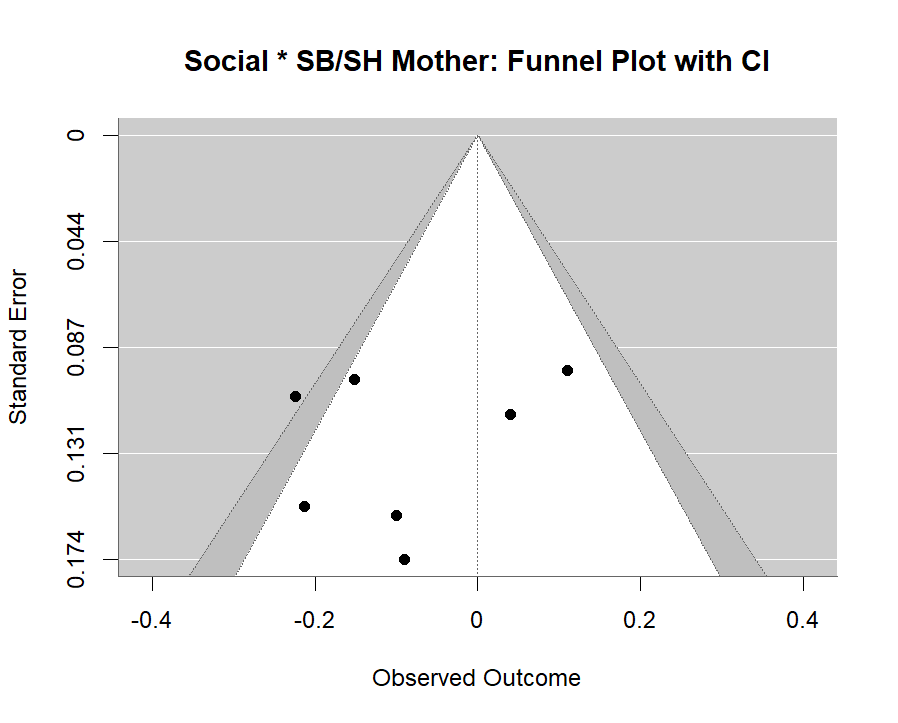


**IVb.7. SB/SH father:**

A random-effects meta-analysis of seven studies yelded a small, non-significant negative effect (r = –0.075, 95% CI: –0.188 to 0.039). Heterogeneity was low to moderate (τ² = 0.009, I² = 36.7%, Q(6) = 9.6, p = .14), suggesting some variability across studies, but not at a statistically significant level. No evidence of publication bias was detected (Egger’s test: z = 0.80, p = .426), and the Trim and Fill procedure did not impute any missing studies, reinforcing the robustness of the findings.
***Meta-regression*** on moderators approached significance (QM(4) = 9.23, p = .055), revealing gender, B = 0.01, p = .019 (.003, .029), and risk status, B = -0.25, p = .047 (-.499, -.003) as significant moderators. The model explained all heterogeneity (R² = 100%). Residual heterogeneity was not statistically significant (QE(2) = 0.38, p = .834), indicating acceptable model fit.


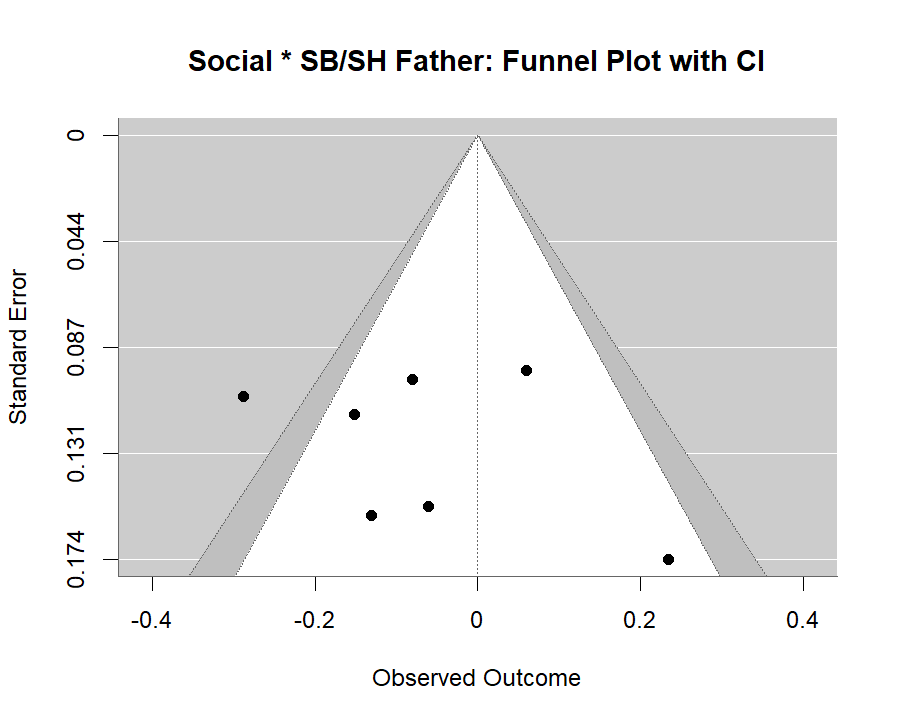
**
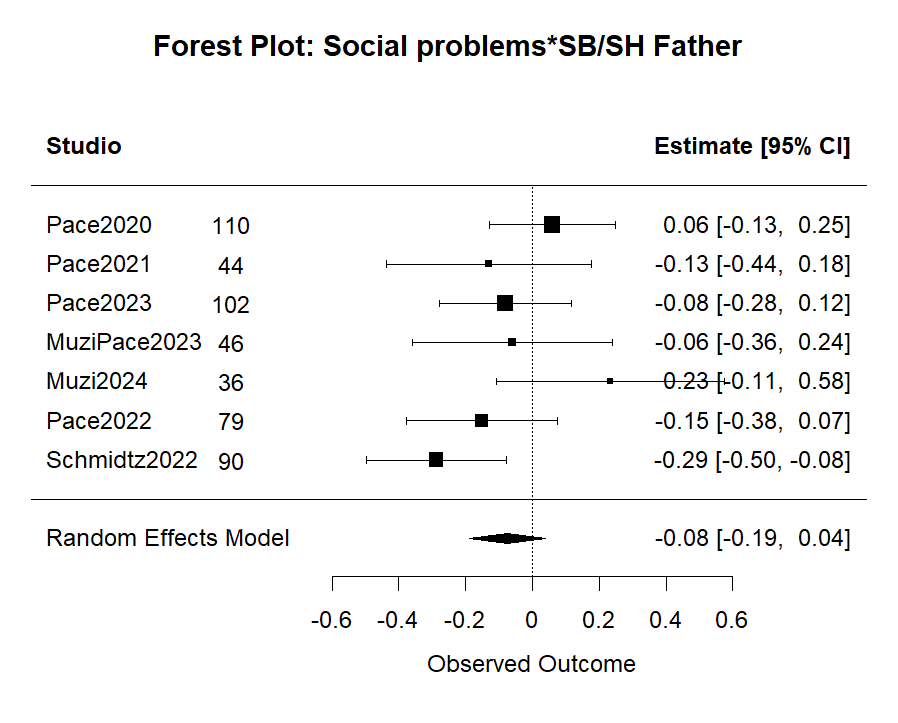
**

**IVc. Thought problems.**

**IVc.1. Security:**

The aggregated correlation indicated non-significant negative effect (*r* = –0.07, 95% CI [–0.16, 0.01]). There was no heterogeneity (*τ²* = 0, *I²* = 0%), with a non-significant Q-test, Q(6) = 5, *p* = .550, suggesting no between-study variability. Egger’s test indicated no potential publication bias (*z* = –1.94, *p* = .053). The trim-and-fill procedure imputed three missing samples, adjusting the effect to lower *r* = -0.04 (95% CI [–0.12, 0.04]).
***Meta-regression*** (*k* = 7) showed no residual heterogeneity to explain, *τ²* = 0, *R*² = 0.0%, *QE*(2) = 0.68, *p* = .711). None of the moderators—sample risk, gender, age, or quality—were significant predictors of the effect size (*QM*(4) = 4.23, *p* = .370).


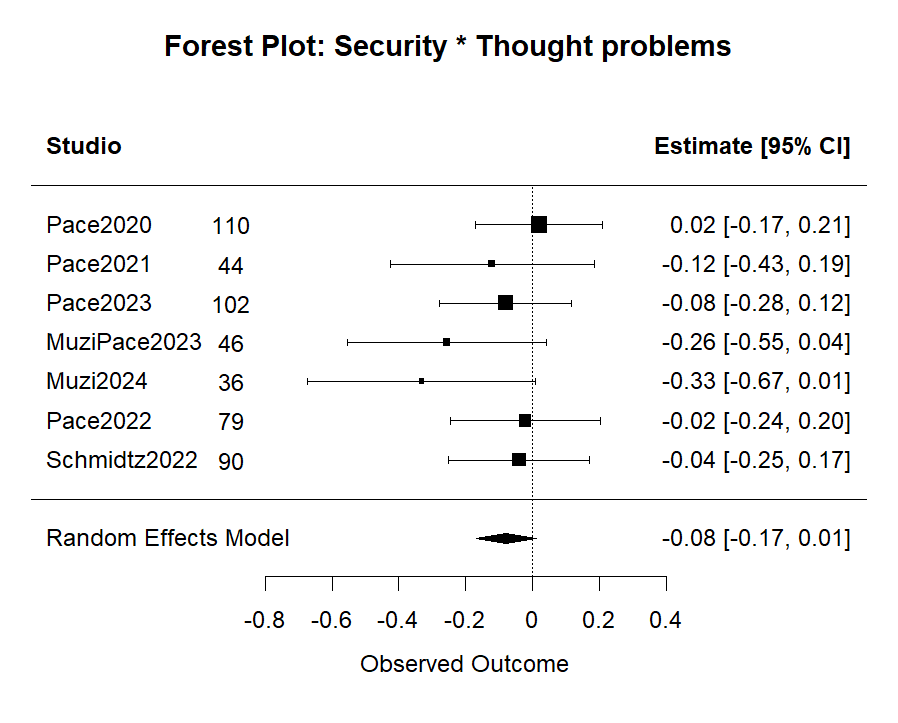
**
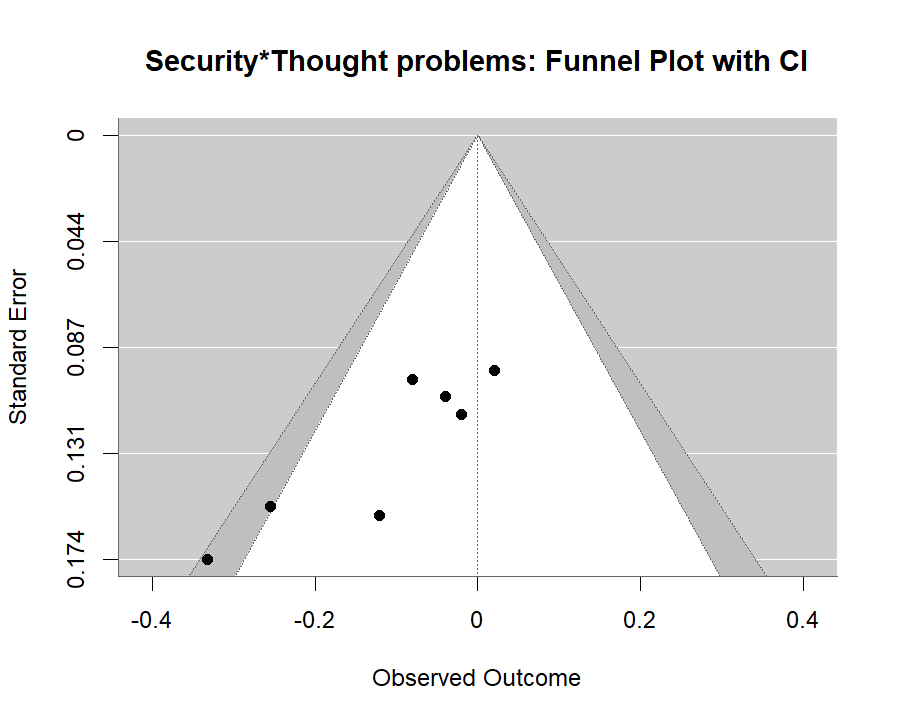
**

**IVc.2. Dismissing:**

The aggregated correlation showed a small, non-significant negative effect (r = –0.11, 95% CI [–0.38, 0.17]). There was substantial heterogeneity across studies (τ² = 0.132, I² = 89.9%), with a significant Q-test, Q(6) = 75.2, p < .001, indicating considerable between-study variability. Egger’s test did not suggest publication bias (z = 1.13, p = .259), and the trim-and-fill procedure imputed 2 missing studies, yielding an adjusted effect of r = –0.23 (95% CI [–0.46, 0.03]). Meta-regression (k = 7) revealed high unexplained heterogeneity (τ² = 0.279, R² = 0.0%, QE(2) = 54.10, p < .001), and none of the moderators—sample risk, gender, age, or study quality—significantly predicted effect sizes (QM(4) = 0.87, p = .929).


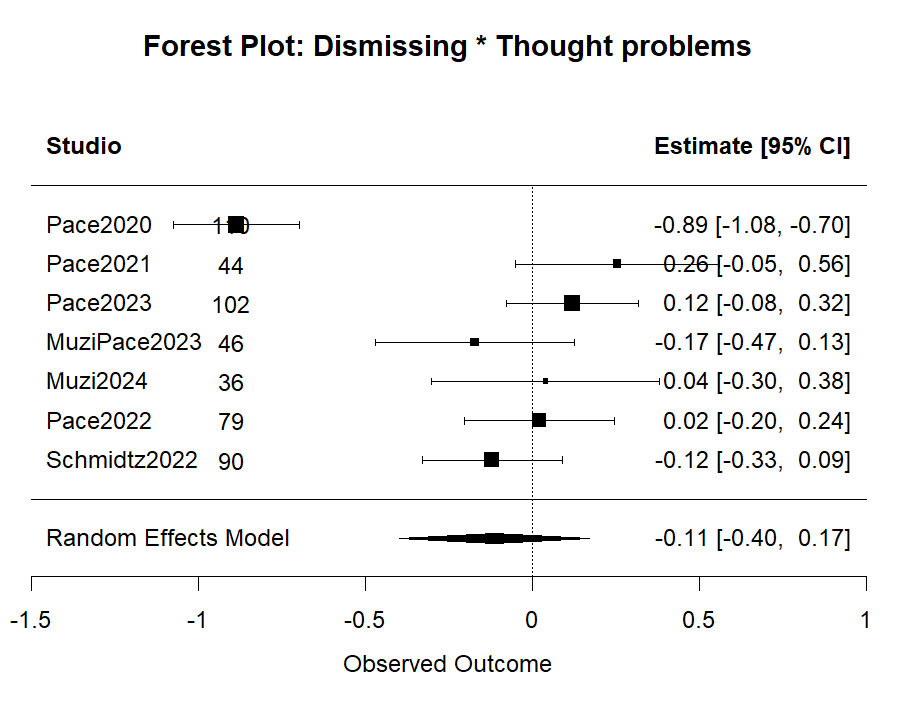

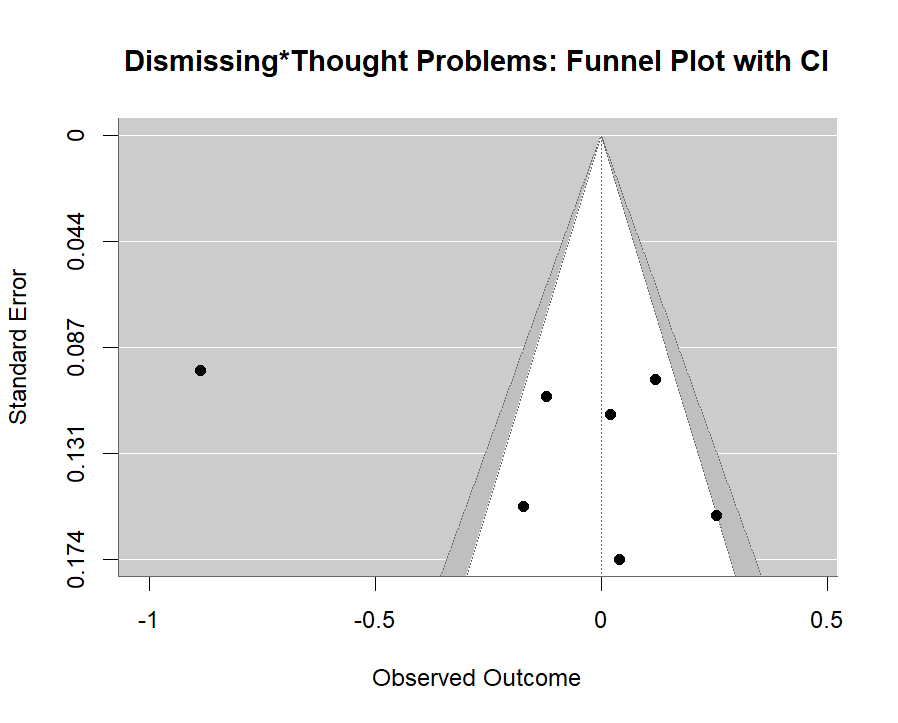


**IVc.3. Preoccupation:**

The meta-analysis yielded a small, non-significant positive effect (r = 0.08, 95% CI [–0.04, 0.21]). There was moderate heterogeneity (τ² = 0.012, I² = 45.6%), with a non-significant Q-test, Q(6) = 11.2, p = .082, indicating some but not statistically significant between-study variability. Egger’s test showed no evidence of publication bias (z = 0.33, p = .742), and the trim-and-fill procedure imputed one missing study, adjusting the effect size slightly to r = 0.06 (95% CI [–0.07, 0.18]). Meta-regression (k = 7) suggested significant high unexplained residual heterogeneity (τ² = 0.035, I² =73.67%, QE(2) = 2.72, p = .021). None of the moderators was statistically significant, QM(4) = 1.53, p = .821.

**IVc.4. Disorganization:**

The meta-analysis revealed a small but statistically significant positive effect (r = 0.14, 95% CI [0.04, 0.24]). There was no evidence of heterogeneity across studies (τ² = 0, I² = 0%), confirmed by a non-significant Q-test, Q(6) = 0.5, *p* = .99. Egger’s test indicated no publication bias (z = 0.18, p = .856), and the trim-and-fill method did not impute any missing studies, leaving the effect size unchanged.
***Meta-regression*** (k = 6) showed no residual heterogeneity (τ² = 0, R² = 0.0%, QE(1) = 0.12, p = .727), and none of the examined moderators—sample risk, gender, age, or study quality—significantly predicted the effect size (QM(4) = 0.367, p = .985).


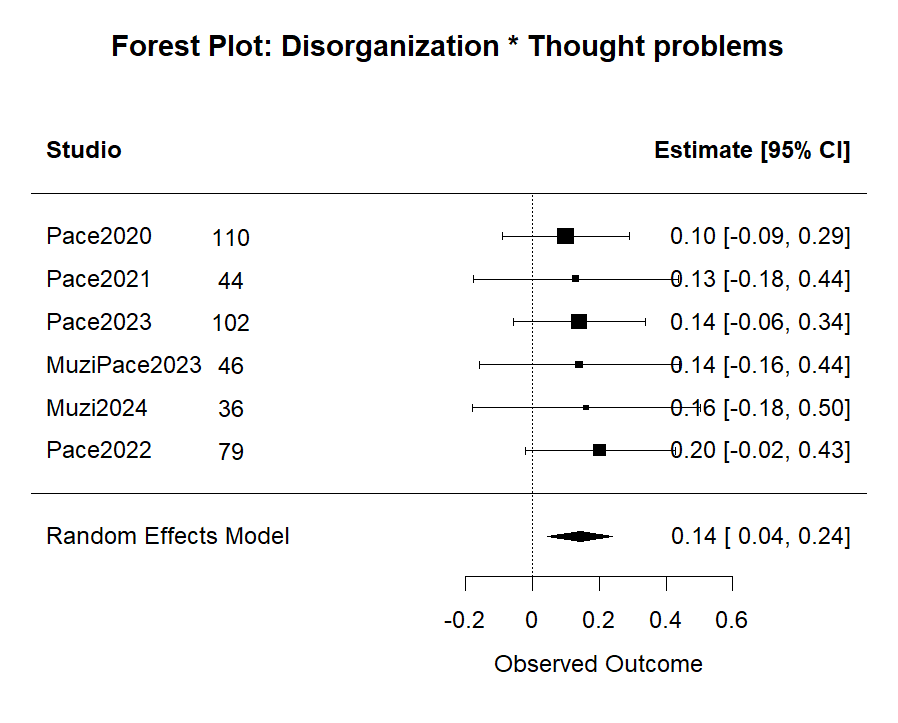

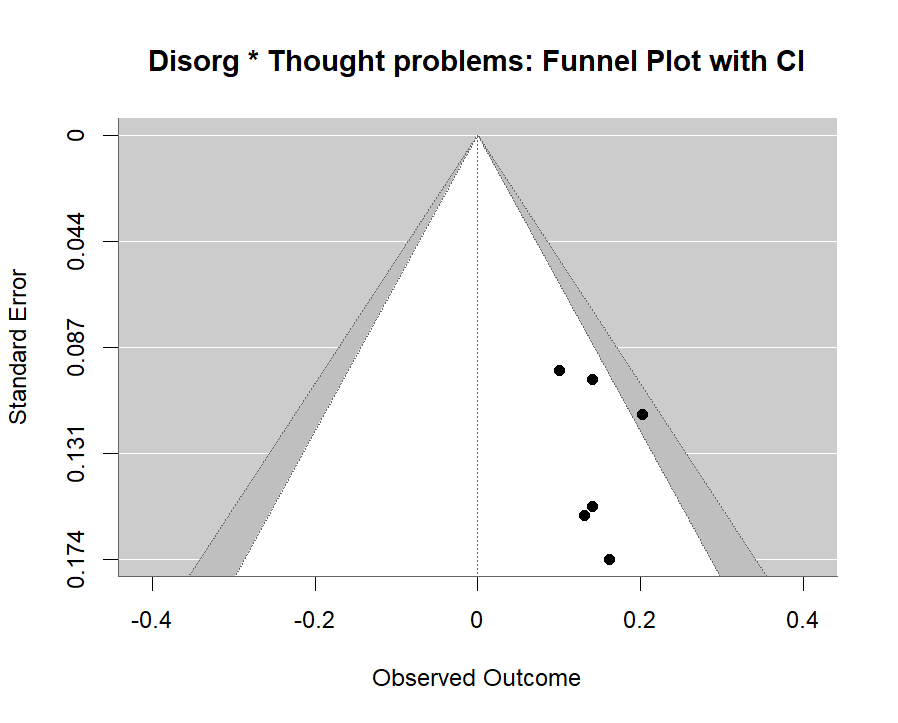


**IVc.5. Overall Coherence:**

The meta-analysis revealed a small, non-significant negative effect (r = -0.072, 95% CI [-0.160, 0.017]). There was no evidence of between-study heterogeneity (τ² = 0, I² = 0%), with a non-significant Q-test, Q(6) = 4.7, p = .59. Egger’s test indicated possible publication bias (z = -1.98, p = .048), although the trim-and-fill method imputed only one study, slightly adjusting the estimate to r = -0.053 (95% CI [-0.138, 0.032]).
***Meta-regression*** (k = 7) identified no significant moderators—sample type, gender, age, or study quality (QM(4) = 4.24, p = .375)—and explained no additional heterogeneity (τ² = 0, R² = 0%).

***
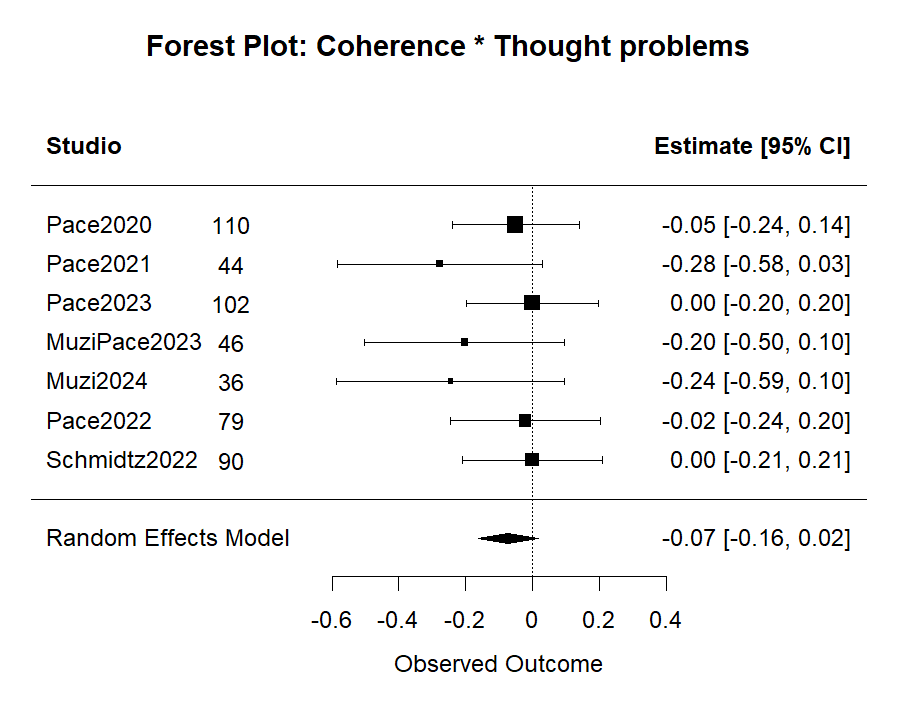
***
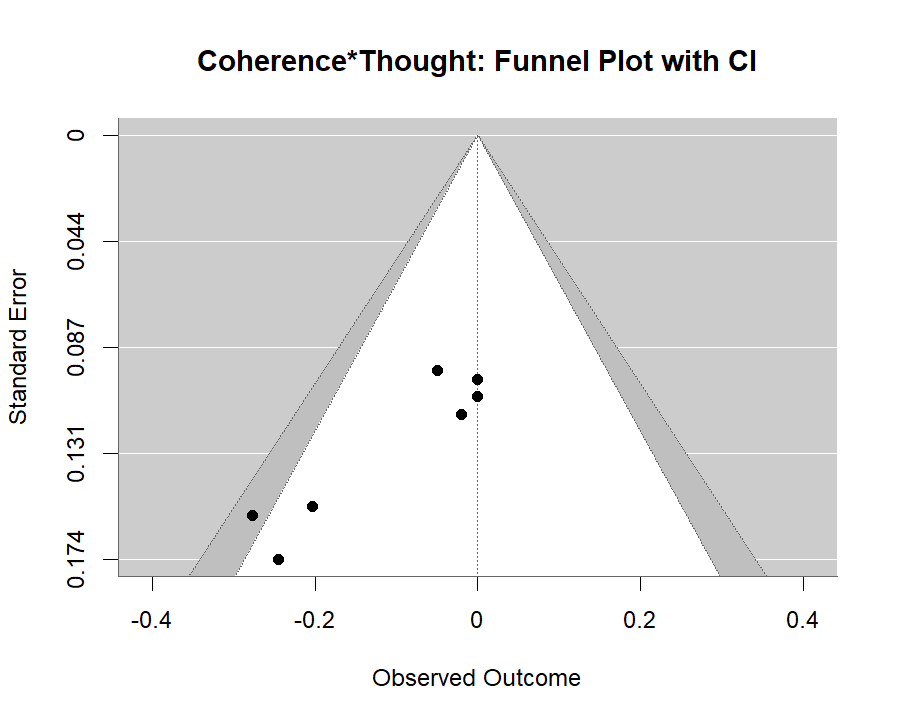


**IVc.6. SB/SH mother**:

The meta-analysis found a small, non-significant negative effect (r = -0.056, 95% CI [-0.155, 0.044]). Heterogeneity was low (τ² = 0.003, I² = 18%), and the Q-test for heterogeneity was not significant (Q(6) = 6.7, p = .35). Egger’s test did not indicate significant publication bias (z = -1.04, p = .299). However, the trim-and-fill procedure imputed two missing studies, adjusting the effect size toward null (r = -0.002, 95% CI [-0.107, 0.104]).
***Meta-regression*** (k = 7) including sample type, gender, age, and study quality as moderators revealed no significant effects (QM(4) = 1.74, p = .783), and explained no heterogeneity (R² = 0%). Residual heterogeneity remained moderate (τ² = 0.006, I² = 34%), suggesting that other unmeasured factors may influence the relationship.


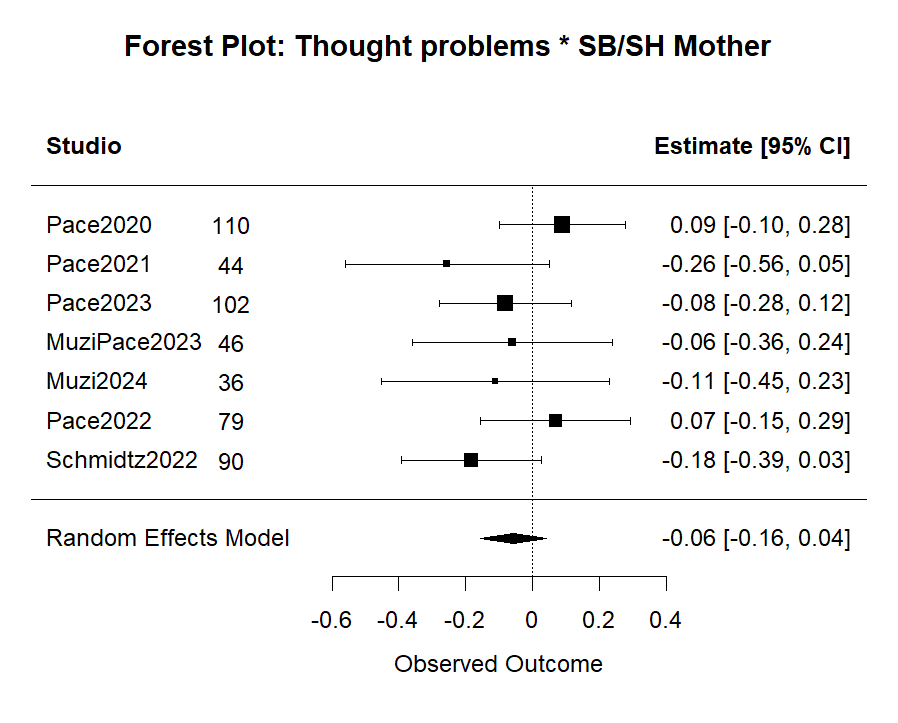

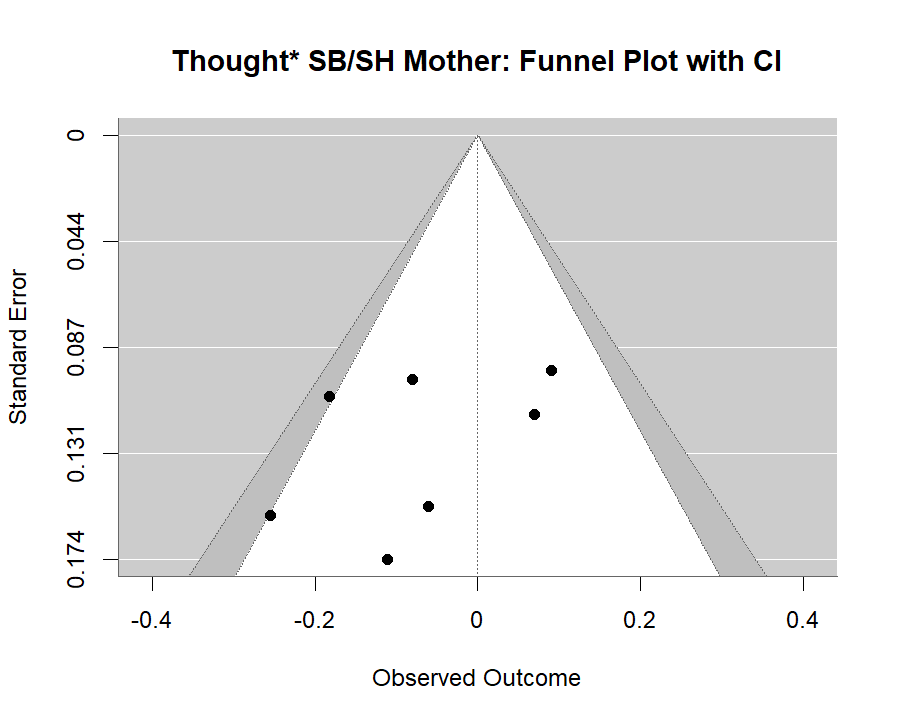


**IVc.7. SB/SH father:**

The meta-analysis revealed a small, non-significant negative association (r = -0.058, 95% CI [-0.159, 0.045]). Between-study heterogeneity was low (τ² = 0.004, I² = 22.4%), and the Q-test was not significant (Q(6) = 7.8, p = .25), indicating limited variability beyond chance.

Egger’s test showed no evidence of publication bias (z = 0.57, p = .568), and the trim-and-fill procedure did not impute additional studies (k = 0), suggesting that the effect estimate is stable.

A ***meta-regression*** (k = 7) indicated no residual heterogeneity (R² = 100%), QE(2) = 1.70, p = .427, and the analysis incorporating sample type, gender, age, and study quality did not identify any significant moderators (QM(4) = 6.10, p = .191).


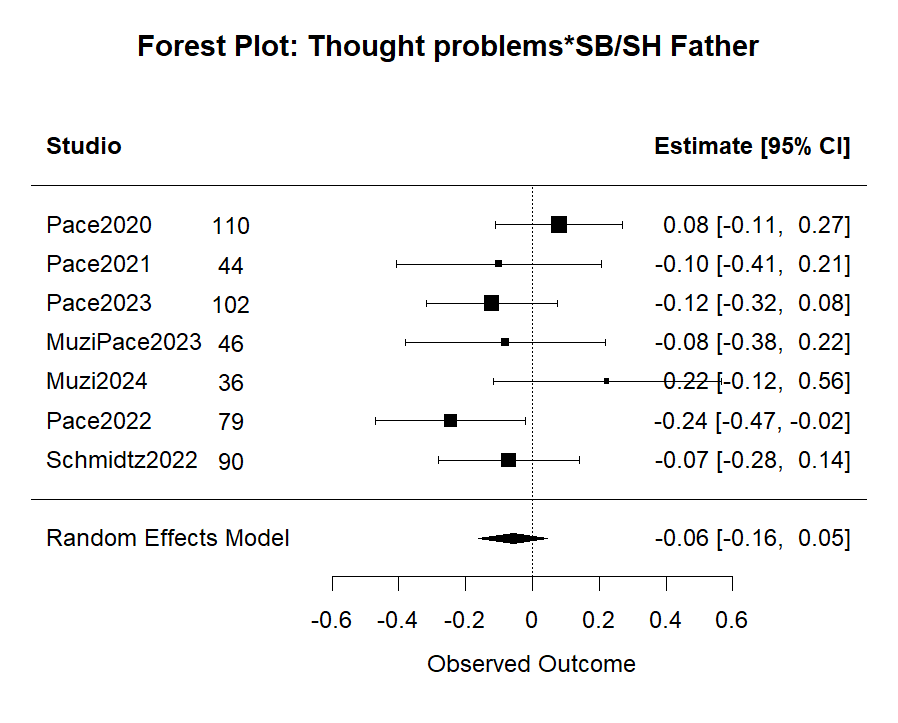

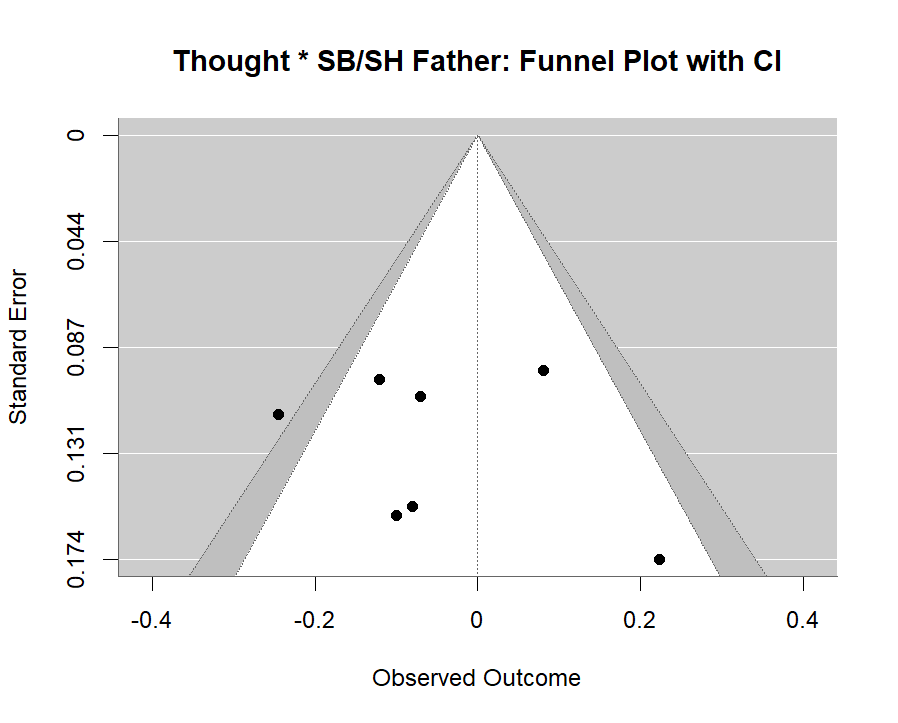


**V. Verbal IQ**

**V.1. Security:**

The aggregated correlation with security indicated small, significant effect (*r* = 0.25, 95% CI [0.06, 0.42]). There was high heterogeneity (*τ²* = 0.05, *I²* = 76.4%), with a significant Q-test, Q(6) = 24.3, *p* <.004, suggesting between-study variability. Egger’s test indicated no potential publication bias (*z* = –0.78, *p* = .434), and the effect remained significant and slightly raise after being adjusted with the trim-and-fill procedure, *r* = 0.31 (95% CI [0.1, 0.49]).
***Meta-regression*** (*k* = 7) explained all residual heterogeneity (*R*² = 100%), with no significant residual heterogeneity left (*QE*(2) = 2.53, *p* = .282). Moderators investigated were significant (*QM*(4) = 21.79, *p* < .002), specifically age (*r* = -0.17, *p* <.001 [-0.27, -0.08]), gender (*r* = -0.04, *p* <.001 [-0.07, -0.02]), and sample risk status (*r* = 0.59, *p* <.001 [0.29, 0.90]).


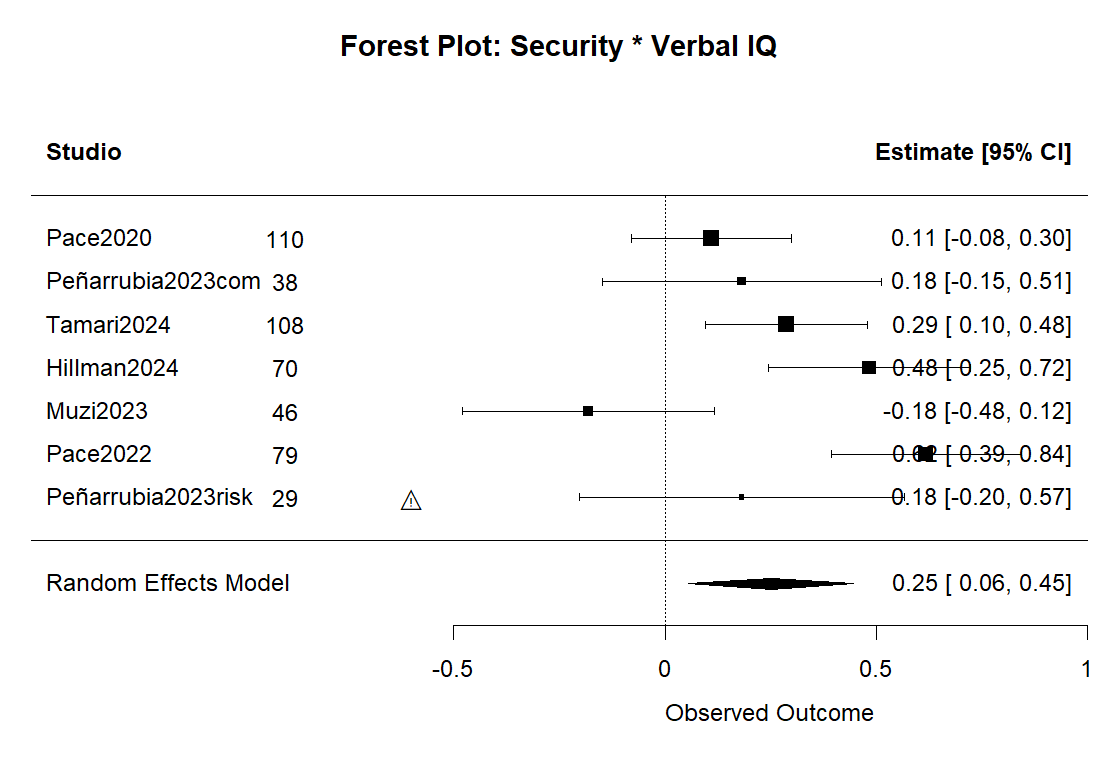

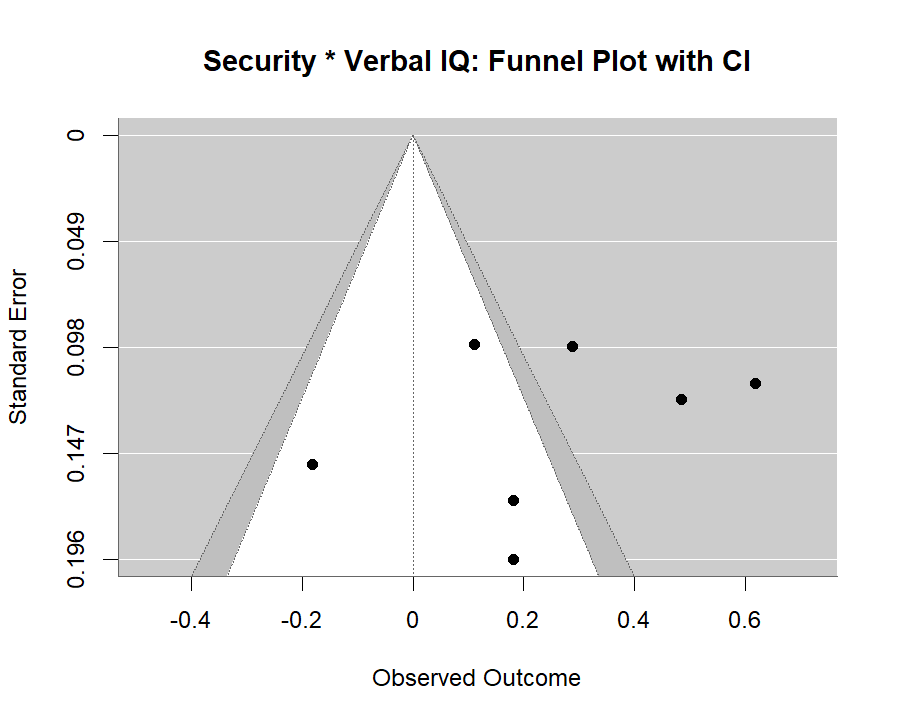


**V.2. Dismissing:**

The aggregated correlation with dismissing indicated small, significant negative effect (r = -0.26, 95% CI [-0.36, -0.16]). There was moderate heterogeneity (τ² = 0.01, I² = 25.3%), with a not-significant Q-test, Q(6) = 7.7, p =.260, suggesting no between-study variability. Egger’s test indicated no potential publication bias (z = 0.56, p = .576), and the effect remained similar and significant after being adjusted with the trim-and-fill procedure (r = -0.28 (95% CI [–0.38, -0.18]).

***Meta-regression*** (k = 7) explained all residual heterogeneity (R² = 100%), with no significant residual heterogeneity left (QE(2) = 0.46, p =.792), but none of the investigated moderators was significant (QM(4) = 7.24, p =.124).


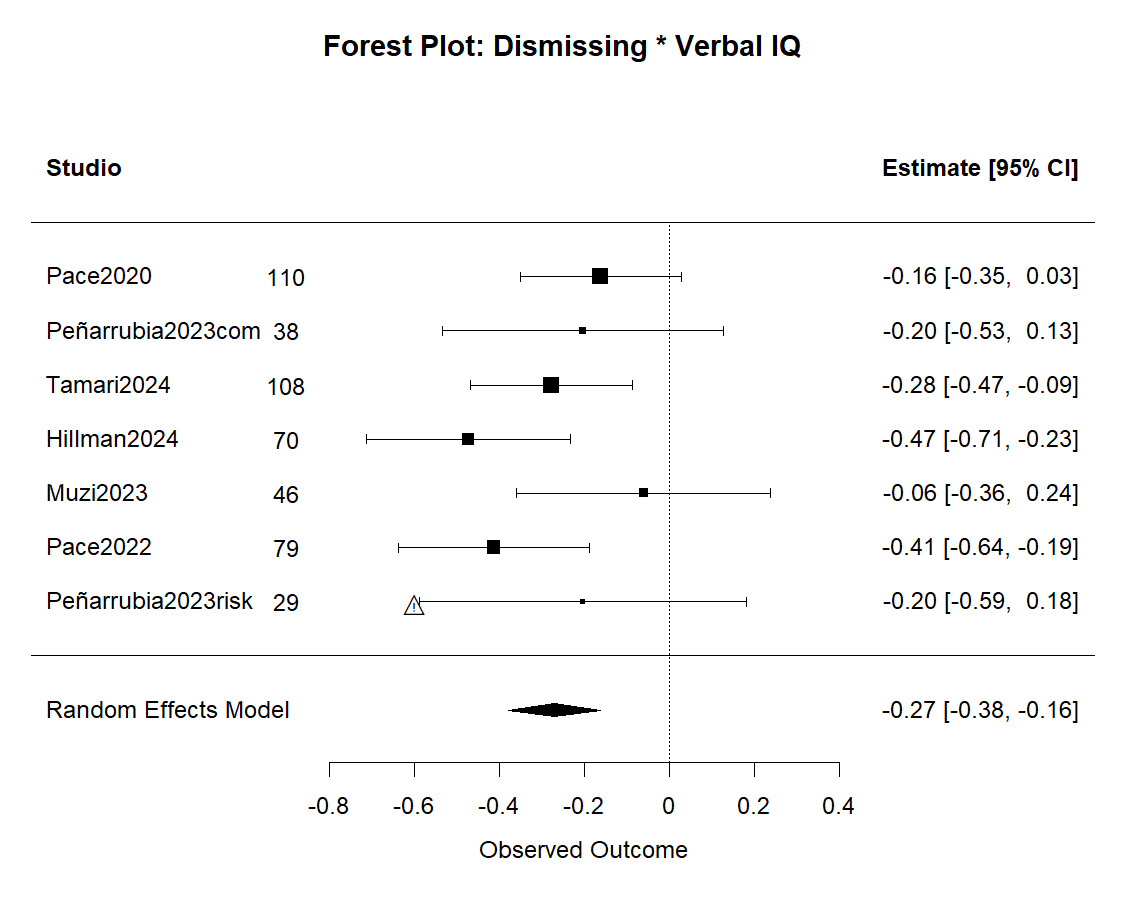

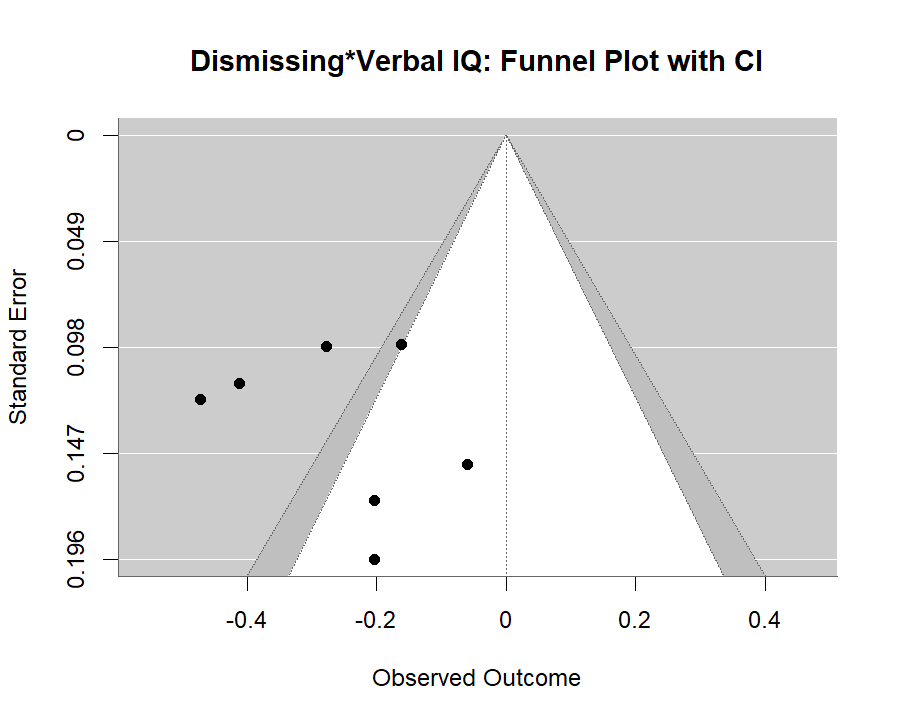


**V.3. Preoccupation:**

The meta-analysis yielded a almost null, non-significant positive effect (r = -0.02, 95% CI [–0.09, 0.14]). Heterogeneity was moderate (τ² = 0.01, I² = 34.3%), but the Q-test was not significant (Q(6) = 8.6, *p* = .200), indicating no between-study variability. Egger’s test did not detect funnel plot asymmetry (z = 0.63, p = .525). The trim-and-fill method did not impute additional studies, and the ES remains similar, r = 0.03, 95% CI [–0.09, 0.14], which remained non-significant.

***Meta-regression*** (*k* = 7) explained all residual heterogeneity (R² = 100%), with no significant residual heterogeneity left (QE(2) = 1.53, p =.464), and none of the investigated moderators was significant (QM(4) = 7.03, p =.134).


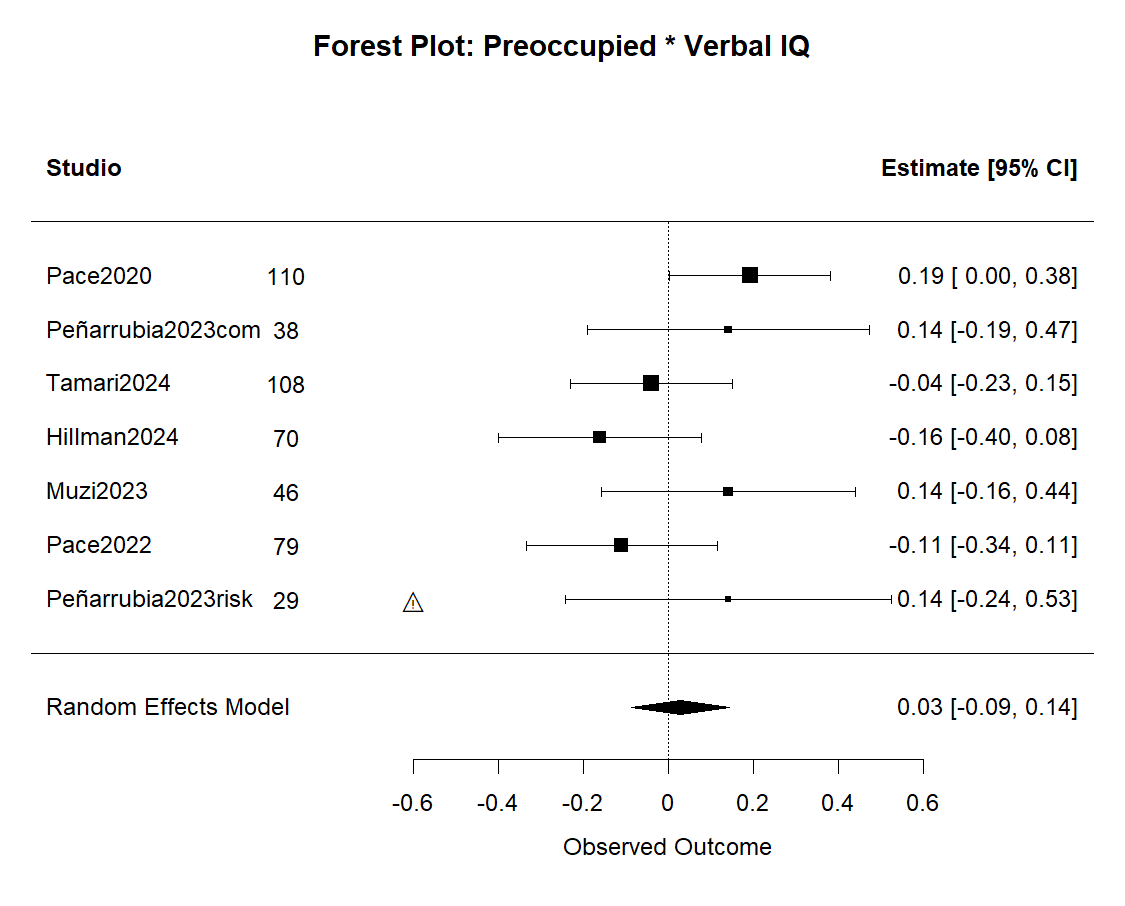

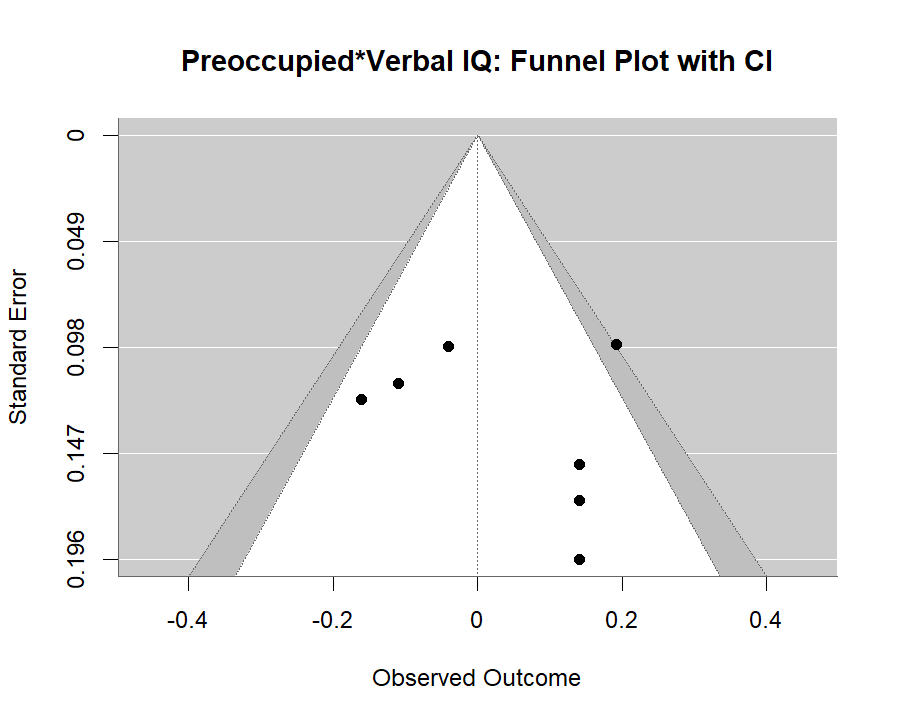


**V.4. Disorganization:**

The aggregated correlation with security indicated almost null not-significant effect (r = -0.06, 95% CI [-0.23, 0.11]). There was high heterogeneity (τ² = 0.03, I² = 60%), with a significant Q-test, Q(6) = 13.3, p =.021, suggesting between-study variability. Egger’s test indicated no potential publication bias (z = 0.14, p = .888), and the effect remained non- significant and the same after the trim-and-fill procedure (r = -0.06 (95% CI [–0.23, 0.11])

***Meta-regression*** (k = 7) explained all residual heterogeneity (R² = 100%), with no significant residual heterogeneity left (QE(1) = 0.05, p = .821). Moderatora analysis wassignificant (QM(4) = 13.26, p = .010), revealing sample risk status (r = -0.36, p =.023 [-0.67, -0.05]) as moderator.


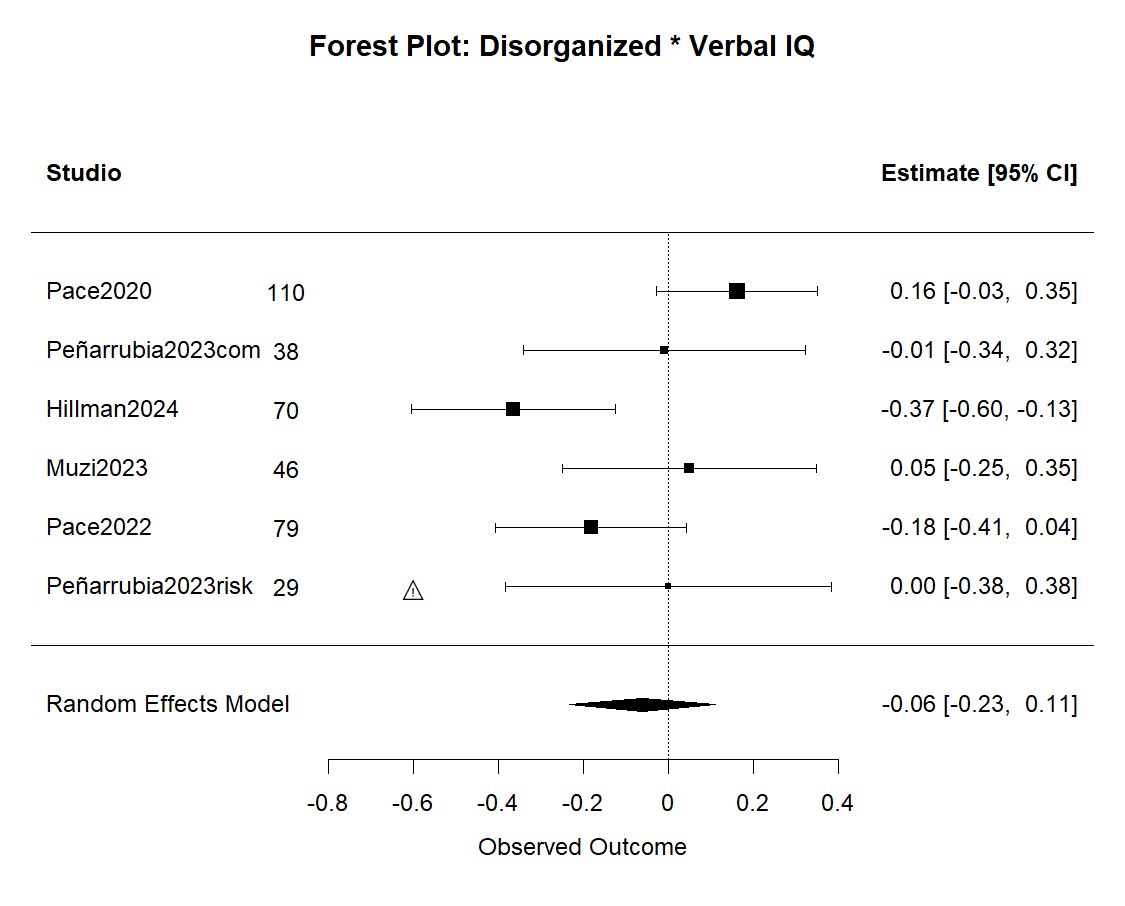

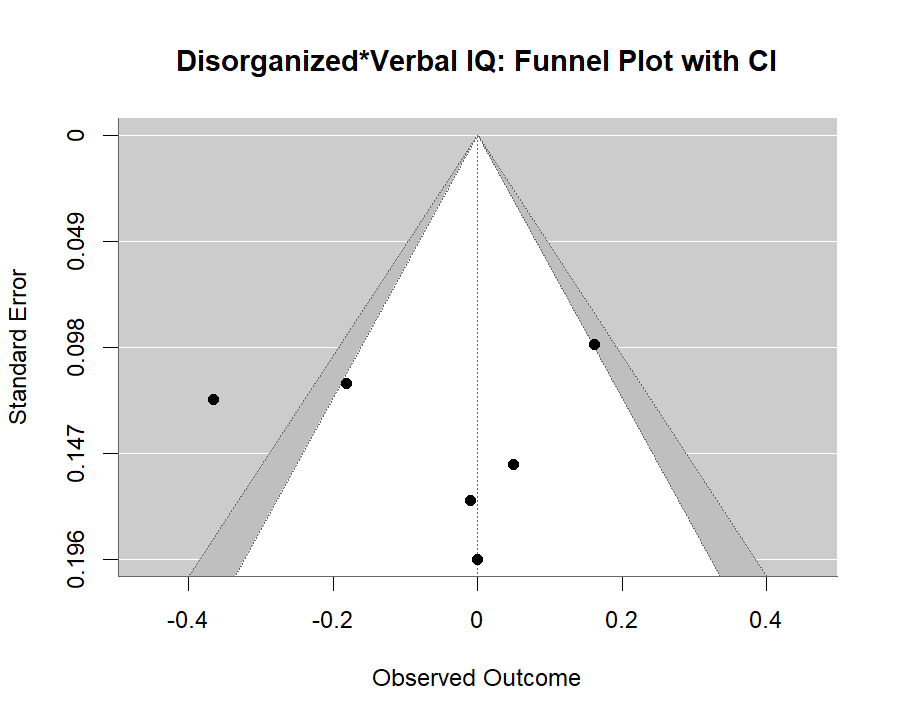


**V.5. Overall Coherence**

The aggregated correlation with coherence indicated small, significant effect (r = 0.27, 95% CI [0.14, 0.42]). There was high heterogeneity (τ² = 0.03, I² = 60%), with a significant Q-test, Q(6) = 15.4, p =.017, suggesting between-study variability. Egger’s test indicated no potential publication bias (z = –0.20, p = .843), and the effect remained similar and significant after being adjusted with the trim-and-fill procedure (r = 0.29 (95% CI [0.14, 0.42]).

***Meta-regression*** (k = 7) explained all residual heterogeneity (R² = 100%), with no significant residual heterogeneity left (QE(2) = 0.01, p = .999). Moderators investigated were significant (QM(4) = 15.38, p = .004), specifically gender (r = -0.03, p =.025 [-.050, -.003]), and sample risk status (r = 0.51, p =.001 [.207, .822]).


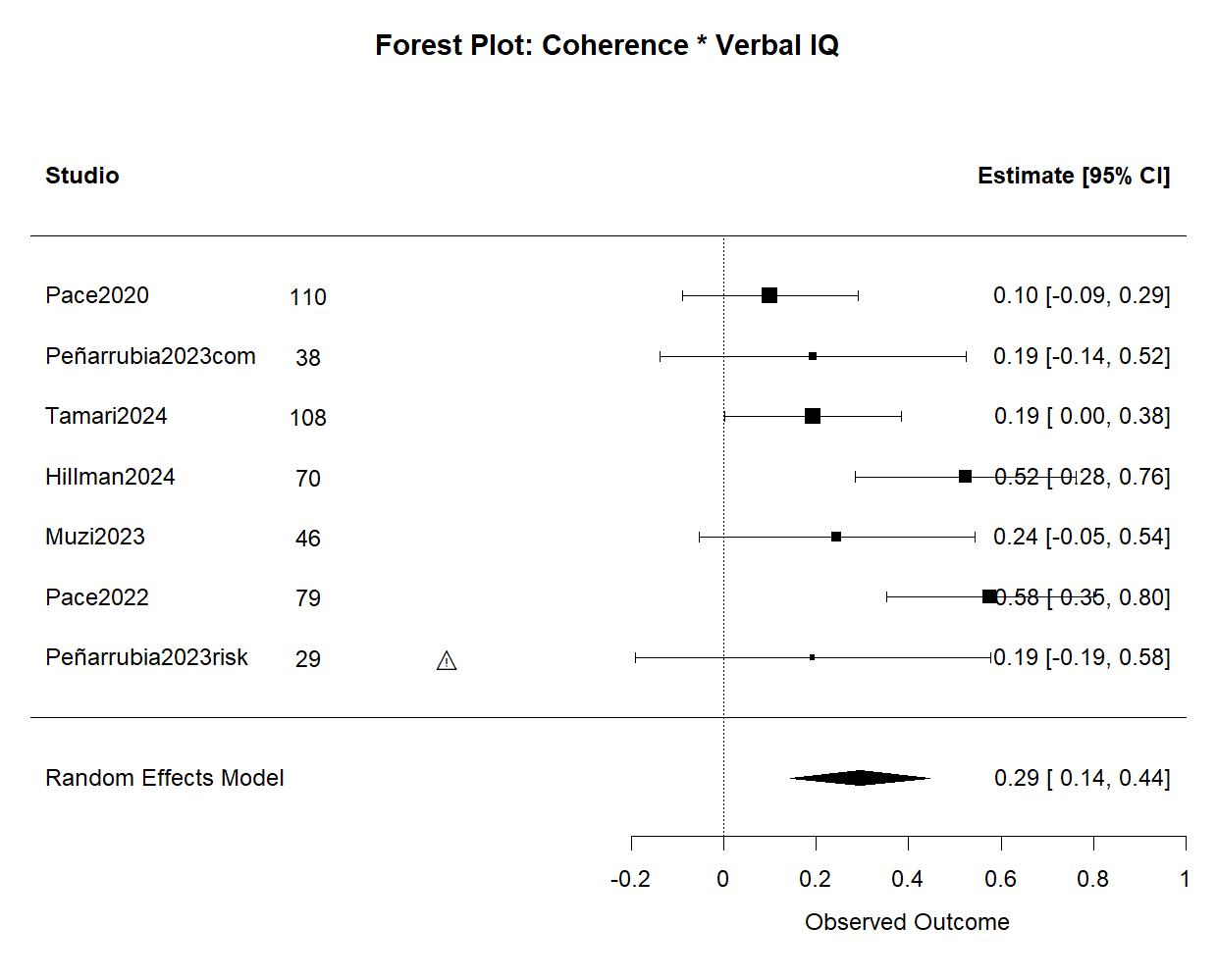

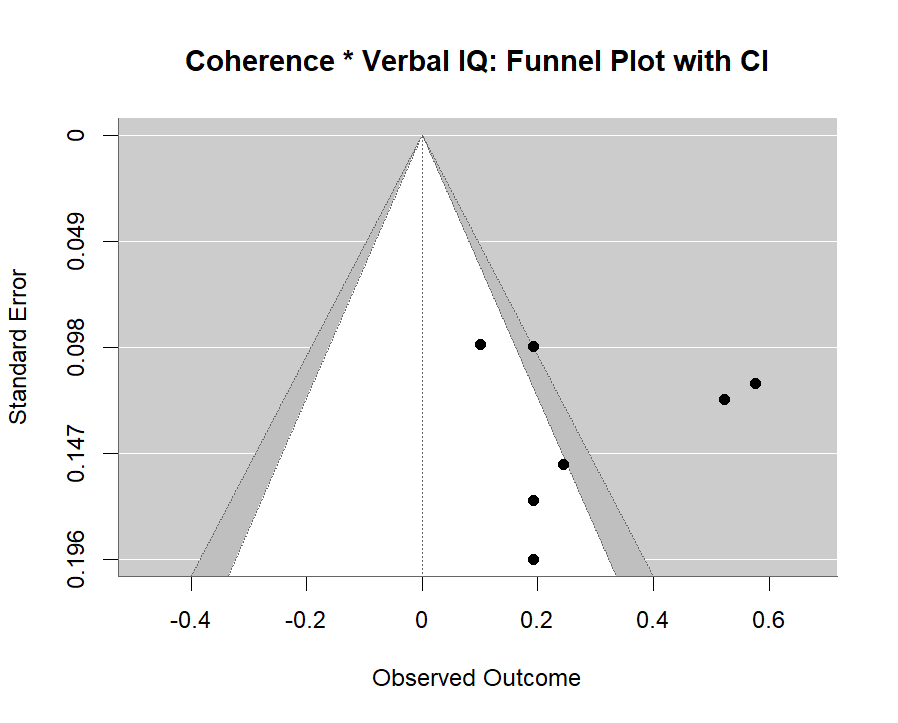


**V.6. SB/SH mother**:

The aggregated correlation indicated a negligible not significant positive effect (r = 0.18, 95% CI [-0.04, 0.32]). There was moderate-to-high heterogeneity (τ² = 0.03, I² = 59.2%), with significant Q-test, Q(6) = 14.1, p =.029, suggesting no between-study variability. Egger’s test indicated no potential publication bias (z = -0.55, p = .581), and the effect remained similar and significant after being adjusted with the trim-and-fill procedure (r = 0.18 (95% CI [0.04, 0.32]).

***Meta-regression*** (k = 7) not explained heterogeneity (R² = 0%), with no significant residual heterogeneity left (QE(2) = 5.17, p =.075) explained by the investigated moderators (QM(4) = 3.82, p =.431).


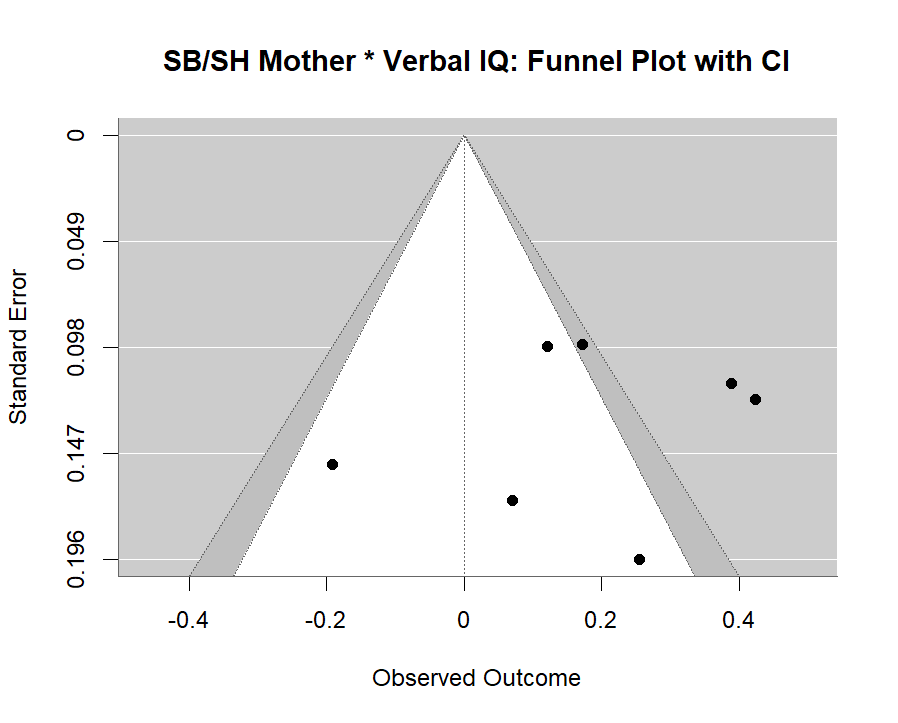

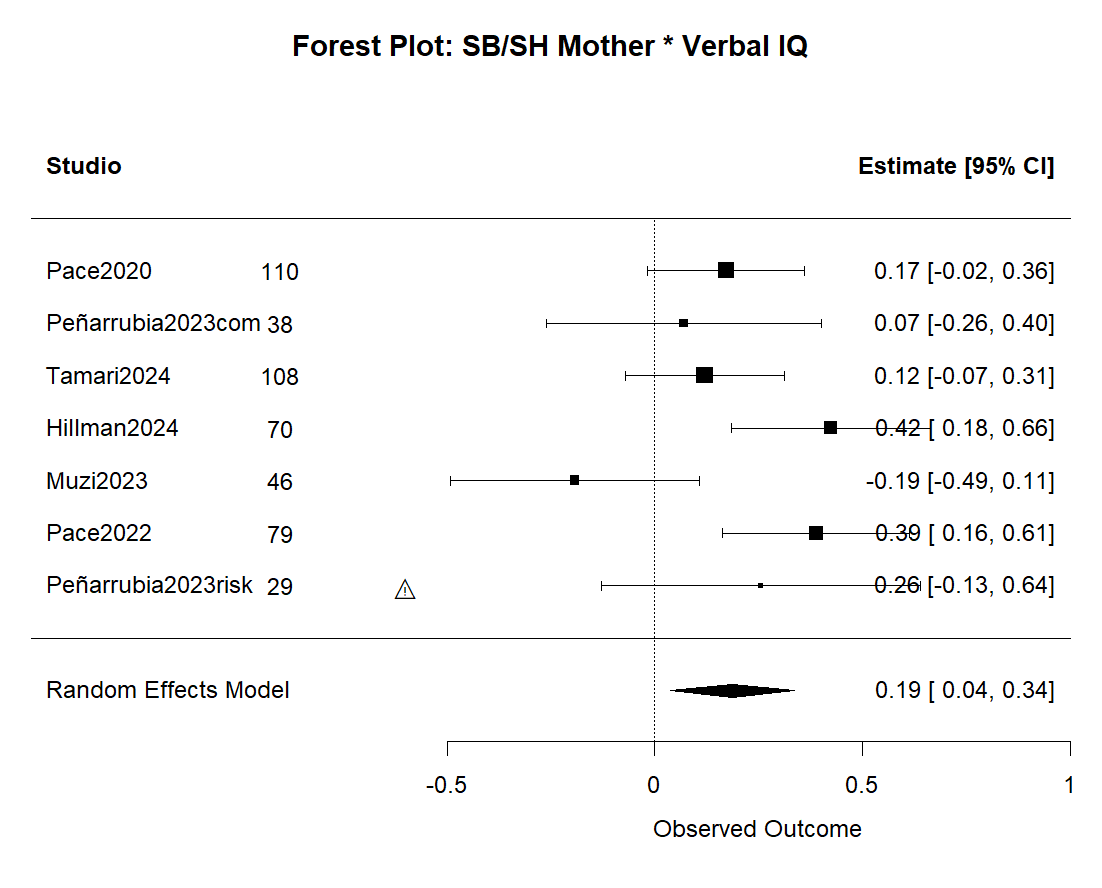


**V.7. SB/SH father:**

The aggregated correlation indicated small, significant effect (*r* = 0.21, 95% CI [0.05, 0.36]). There was high heterogeneity (*τ²* = 0.03, *I²* = 67%), with a significant Q-test, Q(6) = 18.3, *p* =.005, suggesting between-study variability. Egger’s test indicated no potential publication bias (*z* = –0.35, *p* = .730), and the effect remained significant and slightly raise after being adjusted with the trim-and-fill procedure (*r* = 0.21 (95% CI [0.05, 0.36]).
***Meta-regression*** (*k* = 7) explained all residual heterogeneity (*R*² = 100%), with no significant residual heterogeneity left (*QE*(2) = 2.64, *p* = .266). Moderators investigated were significant (*QM*(4) = 15.67, *p* = .005), specifically age (*r* = -0.12, *p* =.012 [-0.22, -0.03]), gender (*r* = -0.04, *p* <.001 [-0.06, -0.02]), and sample risk status (*r* = 0.55, *p* <.001 [0.25, 0.86]).


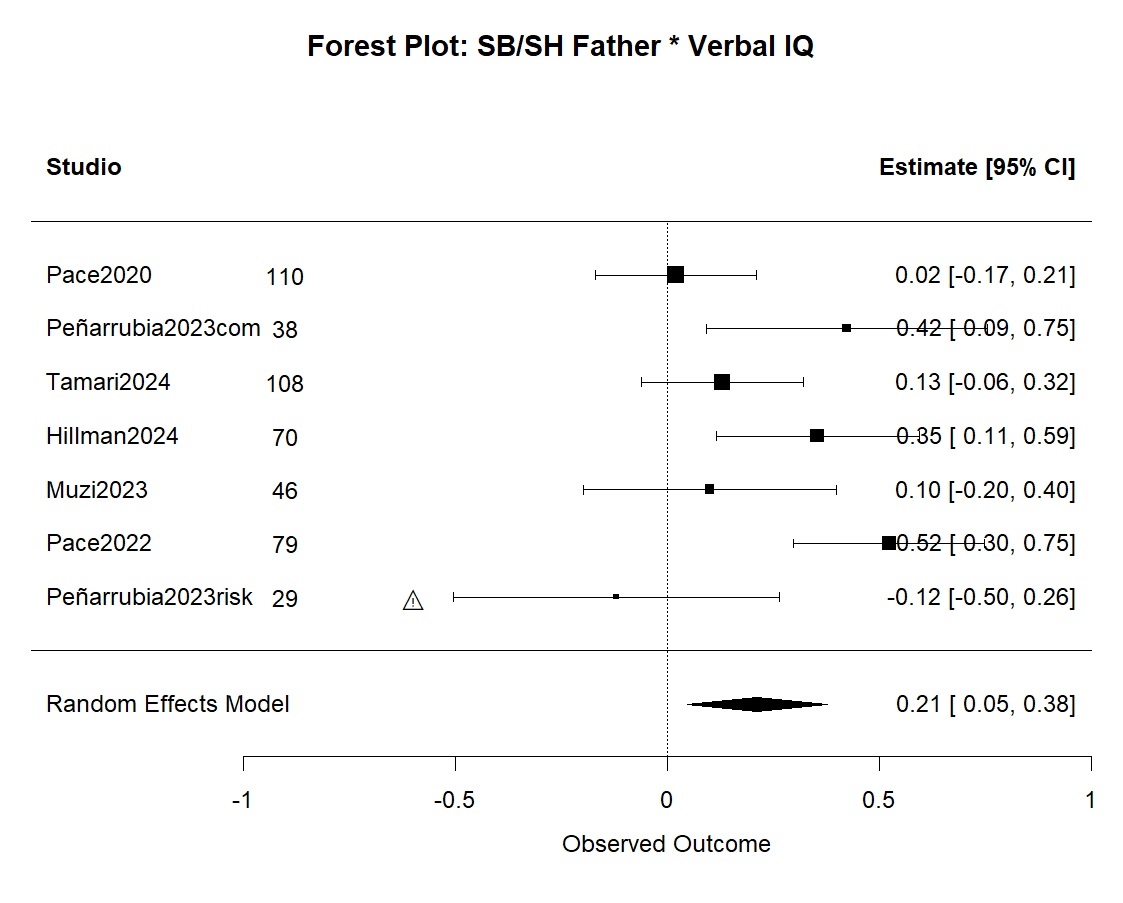

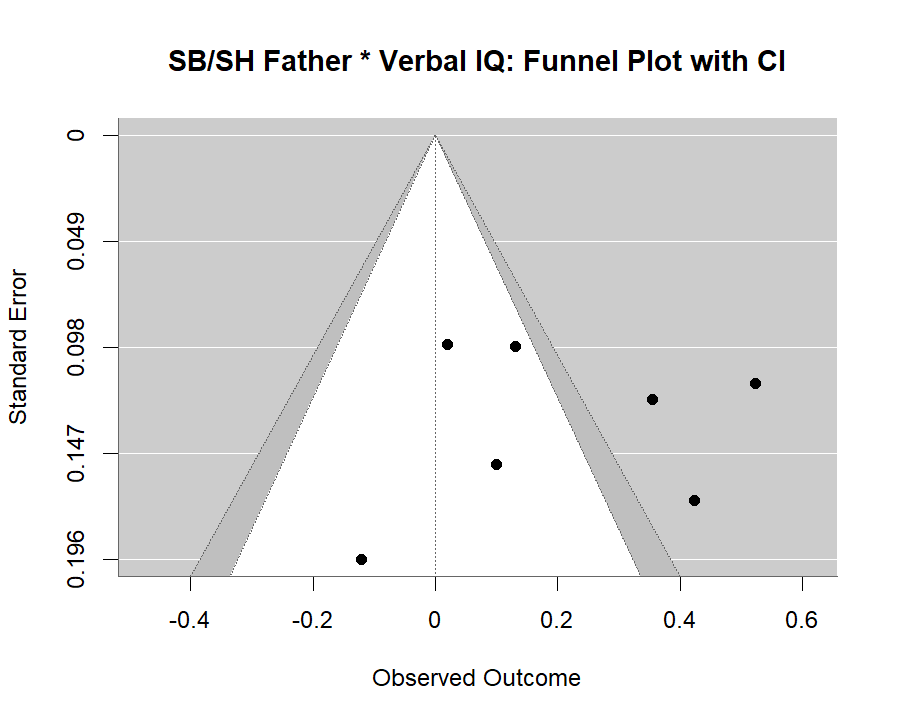


1. In the Funnel plots, grey triangle defines 90% Confidence Intervals, white triangle limits correspond to 95% CI [↑](#footnote-ref-1)
